# Phosphoramidite building blocks with protected nitroxides for the synthesis of spin-labeled DNA and RNA

**DOI:** 10.3762/bjoc.14.133

**Published:** 2018-06-26

**Authors:** Timo Weinrich, Eva A Jaumann, Ute M Scheffer, Thomas F Prisner, Michael W Göbel

**Affiliations:** 1 Institute of Organic Chemistry and Chemical Biology, Goethe University Frankfurt, Max-von-Laue-Str. 7, D-60438 Frankfurt am Main, Germany; 2 Institute of Physical and Theoretical Chemistry, Goethe University Frankfurt, Max-von-Laue-Str. 7, D-60438 Frankfurt am Main, Germany

**Keywords:** EPR, oligonucleotide, PELDOR, photolabile protection, TEMPO

## Abstract

TEMPO spin labels protected with 2-nitrobenzyloxymethyl groups were attached to the amino residues of three different nucleosides: deoxycytidine, deoxyadenosine, and adenosine. The corresponding phosphoramidites could be incorporated by unmodified standard procedures into four different self-complementary DNA and two RNA oligonucleotides. After photochemical removal of the protective group, elimination of formic aldehyde and spontaneous air oxidation, the nitroxide radicals were regenerated in high yield. The resulting spin-labeled palindromic duplexes could be directly investigated by PELDOR spectroscopy without further purification steps. Spin–spin distances measured by PELDOR correspond well to the values obtained from molecular models.

## Introduction

**Figure 1:**
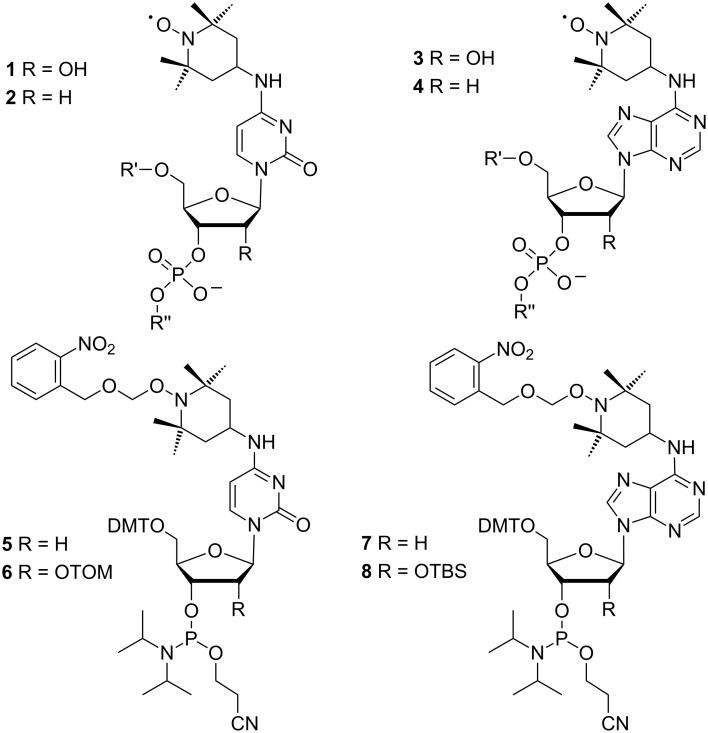
Structures of TEMPO-labeled oligonucleotides and of phosphoramidites **5**–**8**.

EPR spectroscopy is well established to study the structure and dynamics of nucleic acids [1–8]. Although the information attainable by EPR is less detailed when compared to NMR, it is often complementary. While local conformations are normally obtained from NMR data, EPR can measure long distances that are hardly accessible by NMR [9–10]. Furthermore, spin labeling of biopolymers can support NMR studies by paramagnetic relaxation enhancement [11–12]. For nucleic acids, spin labeling is most often achieved by covalent attachment of nitroxides. Unfortunately, the conditions required to assemble oligonucleotides by phosphoramidite chemistry and to ligate them enzymatically, are known to partially degrade nitroxides. This problem can be reduced by postsynthetic introduction of the spin label [13–24]. Starting from convertible nucleotides, for example, nucleophilic displacement by 4-amino-TEMPO has been used to prepare RNA strands containing the cytidine derivative **1** and its adenosine analog **3** [8,25–26] (Figure 1). Alternatively, by adapting the standard synthetic procedures, nitroxides can be directly incorporated into oligonucleotides by phosphoramidite building blocks [27–40]. However, some degradation of the spin labels inevitably will occur. Nitroxide labeled DNA strands of the general structures **2** and **4** have been prepared by this way [30,34,37].

In recent years, we have therefore developed a third strategy based on photolabile protective groups [41–43] finally leading to phosphoramidite **6**. This building block behaves in solid-phase RNA synthesis as any normal TOM protected amidite. It is completely stable against the conditions used for strand assembly, RNA deprotection and enzymatic ligation. Amidite **6** has been applied to synthesize a full-length TAR RNA labeled with two nitroxide precursors. In the last step, the 2-nitrobenzyloxymethyl groups were removed by irradiation at 365 nm. After elimination of formic aldehyde induced by gentle heating, the resulting hydroxylamines spontaneously reacted with air to form the nitroxide radicals in high yield [43]. Although the rigid spin labels introduced by Sigurdsson [31–33], Hopkins [27,29] and Engels [20–21] are generally considered advantageous for EPR studies, TEMPO-modified RNAs according to structures **1** and **3** have been successfully used in PELDOR experiments to determine the distance between two spins [8,25,43]. The apparent usefulness of such building blocks has prompted us in the present study to extend the protection strategy from amidite **6** to its DNA analog **5** and to adenosine derivatives **7** and **8**.

## Results and Discussion

**Scheme 1:**
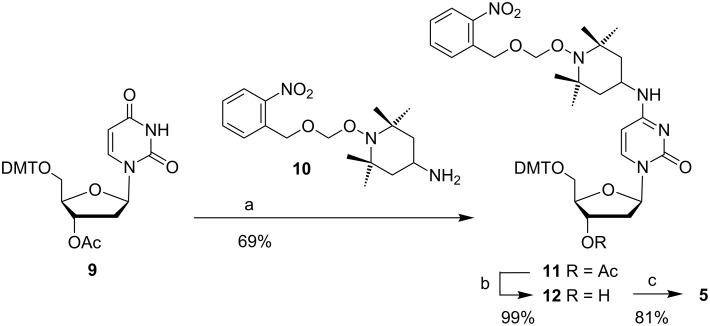
Synthesis of phosphoramidite **5**. Reagents and conditions: (a) 1. 2,4,6-triisopropylbenzenesulfonyl chloride, Et_3_N, 4-dimethylaminopyridine, CH_2_Cl_2_, 0 °C → rt, 19 h; 2. addition of **10**, diisopropylethylamine, DMF, 90 °C, 24 h; (b) MeOH, NaHCO_3_, rt, 2.5 h; (c) *N*,*N*-diisopropylamino(2-cyanoethyl)phosphoramidic chloride, Et_3_N, CH_2_Cl_2_, rt, 20 h.

To prepare compound **5**, the deoxyuridine derivative **9** [44] was activated by sulfonylation and then treated with the protected TEMPO building block **10** [43] to provide **11** (Scheme 1). After deacetylation, phosphitylation of **12** straightforwardly led to amidite **5** in multigram amounts. In contrast to the corresponding nitroxide, all NMR spectra of **5** are well resolved indicating the absence of any paramagnetic moiety.

**Scheme 2:**
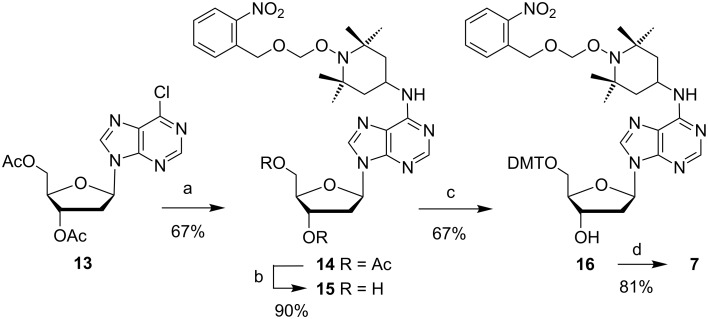
Synthesis of phosphoramidite **7**. Reagents and conditions: (a) Addition of **10**, diisopropylethylamine, 1-propanol, 75 °C, 8 h, rt, 14 h; (b) 7 N NH_3_ in MeOH, 0 °C → rt, 3 h; (c) dimethoxytrityl chloride, pyridine, rt, 23 h; (d) *N*,*N*-diisopropylamino(2-cyanoethyl)phosphoramidic chloride, Et_3_N, CH_2_Cl_2_, rt, 4.5 h.

Unlike compound **9**, the corresponding inosine derivative is known to produce a mixture upon sulfonylation [30], a reaction giving poor yields in our hands. We therefore used the 6-chloro derivative **13** as starting material, easily accessible from deoxyadenosine via enzymatic deamination, acetylation [37] and chlorination. This compound reacted cleanly and yielded 67% of the TEMPO conjugate **14**. After deacetylation (**15**) and tritylation (**16**), amidite building block **7** was obtained by phosphitylation in ample quantity (Scheme 2).

**Scheme 3:**
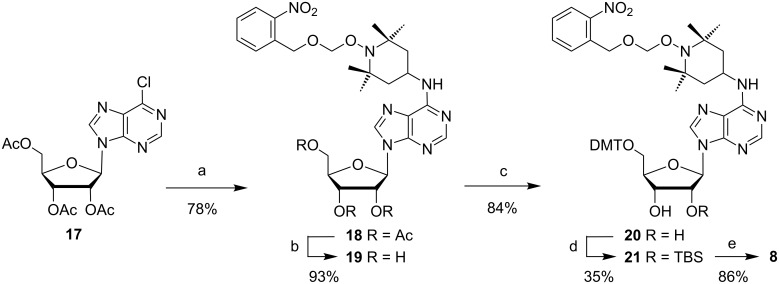
Synthesis of phosphoramidite **8**. Reagents and conditions: (a) Addition of **10**, diisopropylethylamine, 1-propanol, 75 °C, 7 h, rt, 14 h; (b) 7 N NH_3_ in MeOH, 0 °C → rt, 3 h; (c) dimethoxytrityl chloride, pyridine, rt, 20 h; (d) *tert-*butyldimethylsilyl chloride, imidazole, DMF, rt, 22 h; (e) *N*,*N*-diisopropylamino(2-cyanoethyl)phosphoramidic chloride, Et_3_N, CH_2_Cl_2_, rt, 23 h.

Inosine derivatives protected with TOM in 2’-position are accessible but only by a multistep procedure [45]. We therefore decided to synthesize the corresponding 2’-*O*-TBS building block instead (Scheme 3). Starting compound **17** was prepared in a similar way as **13** by enzymatic deamination of adenosine, acetylation [46] and chlorination. A clean reaction with amino-TEMPO compound **10** then produced 78% of compound **18**. Ester hydrolysis (**19**) and tritylation afforded **20** which was silylated with 1.8 equiv of TBS chloride. Although the 3’-silylated and bis-silylated side products could be easily removed and converted back to **20**, the 35% yield of compound **21** obtained in this step limited the availability of amidite **8** compared to its analogs **5** and **7**. Nevertheless, yields of **8** are sufficient to incorporate this type of spin label into RNA strands.

**Figure 2:**
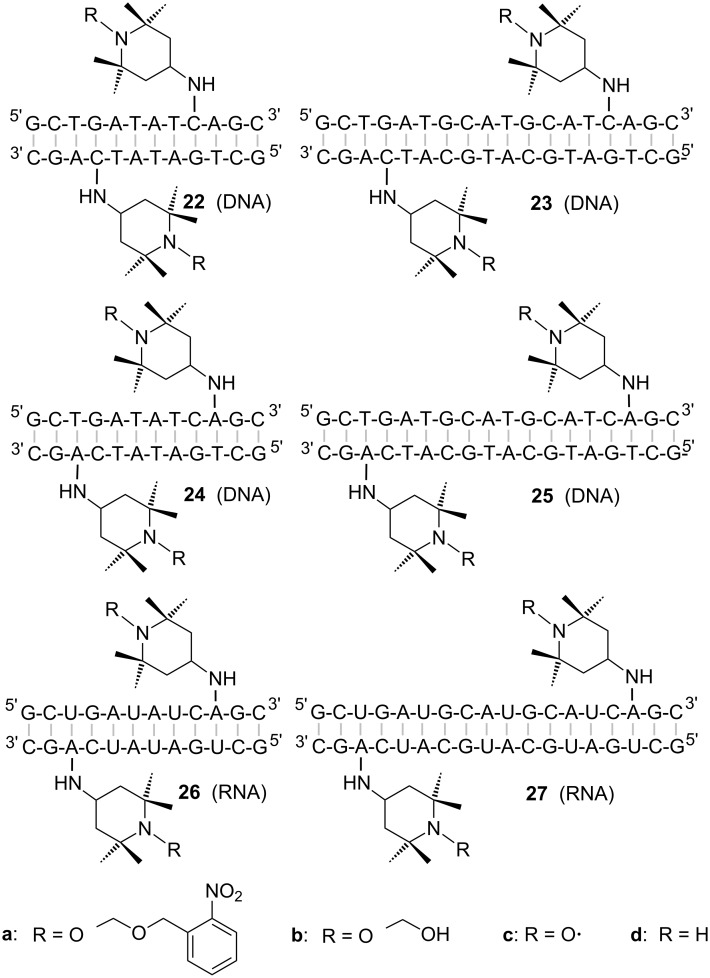
Structures of palindromic oligonucleotides prepared from amidites **5** (**22a**, **23a**), **7** (**24a**, **25a**), and **8** (**26a**, **27a**). Hemiacetals **22b**–**27b** were obtained after photochemical deprotection (custom built apparatus with three LEDs Nichia NCCU033, 365 nm, each with 100 mW optical output power [41]. 20 min irradiation in 10 mM phosphate buffer, pH 7.4). Subsequent elimination of formic aldehyde and air oxidation transformed these intermediates into nitroxides **22c**–**27c**. Amines **22d**–**27d**, typical degradation products of nitroxides, could not be detected.

The phosphoramidite building blocks **5**, **7**, and **8** have been used to prepare a series of self-complementary DNA (**22a**–**25a**) and RNA oligonucleotides (**26a**, **27a**), each containing one protected TEMPO label (Figure 2). Chain assembly and deprotection proceeded uneventfully (Supporting Information File 1, Figures S1–S3). All oligonucleotides were purified by anion exchange and reversed-phase HPLC and characterized by mass spectrometry (Supporting Information File 1, Figures S4–S9). Upon photochemical deprotection (365 nm), the primary products are the hemiacetals **22b**–**27b** (Supporting Information File 1, Figure S10). They still prevent air oxidation of the hydroxylamines and are removed by gentle heating. In case of the 12mers, clean analytical separation of hemiacetals (**22b**, **24b**, **26b**) from nitroxides (**22c**, **24c**, **26c**) was possible by RP-HPLC (Supporting Information File 1, Figure S10). The detection of hemiacetals by HPLC became more troublesome for the 18mers. An indirect clue about their existence comes from cw-EPR spectra measured directly after irradiation indicating imperfect reoxidation of the nitroxides (Supporting Information File 1, Figure S20). To form the palindromic duplexes, samples were submitted to an annealing procedure by heating to 90 °C. This step also completes the conversion of hemiacetals into nitroxide radicals **22c**–**27c** in all cases. It should be noted, however, that palindromic duplexes may coexist in equilibrium with monomeric hairpin structures (see below). HPLC measurements gave no evidence for the formation of reduction products **22d**–**27d**. Hydrolytic degradation of the oligonucleotides was not observed.

**Figure 3:**
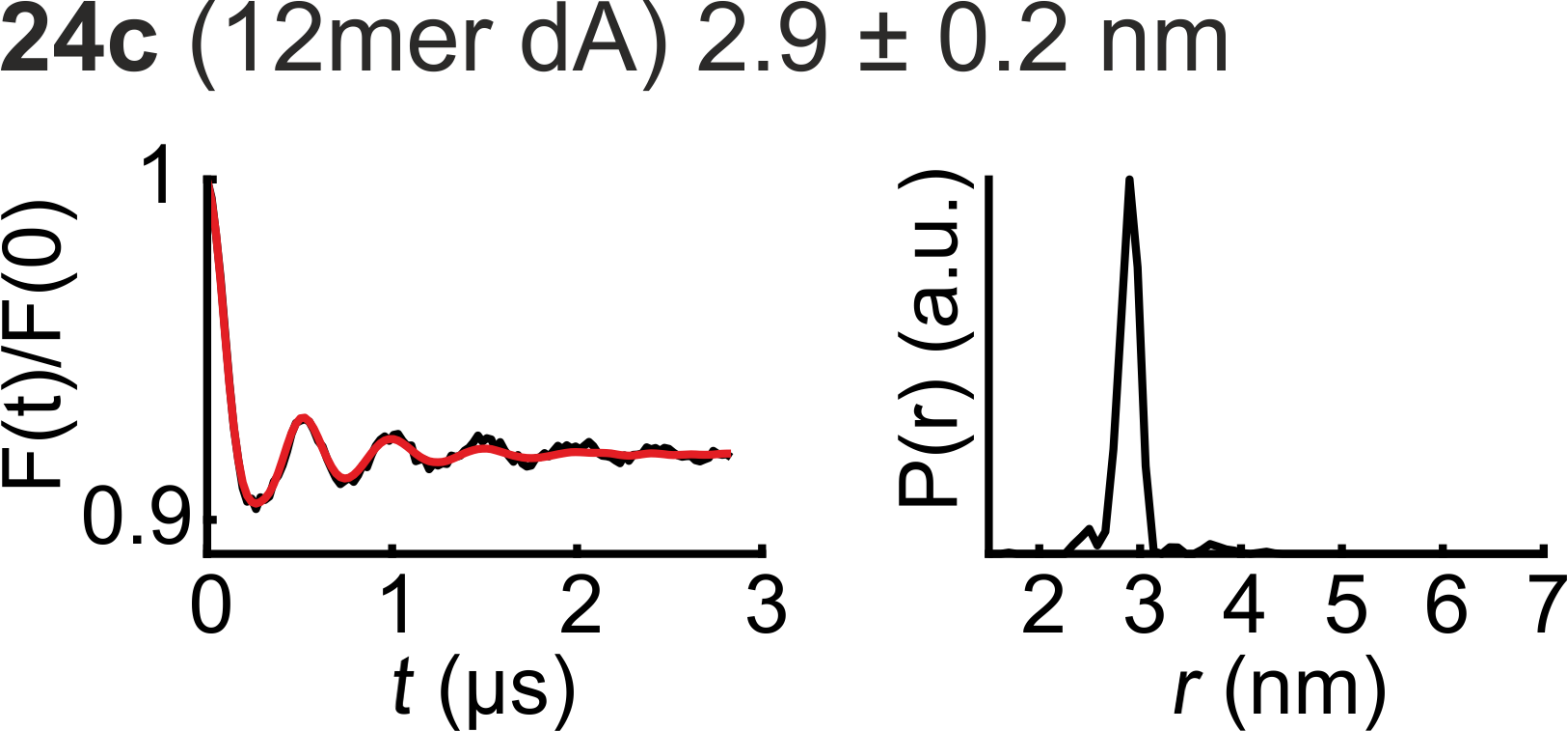
PELDOR measurement of **24c** (12mer dA). The background-corrected time trace (original time trace in Supporting Information File 1, Figure S21) was fitted with Tikhonov regularization (red) with DeerAnalysis15 [48]. The distance distribution shows a well-defined distance of 2.9 ± 0.2 nm.

For successful PELDOR experiments the quality of spin labeled samples is essential. We have shown previously that RNA strands synthesized from amidite **6** are ready for PELDOR studies directly after photochemical deprotection and annealing [43]. No further purification is required. In the same way we have now treated all oligonucleotides **22a**–**27a**. Each strand was irradiated in buffer solution at 365 nm and then heated to 90 °C for 70 min and slowly cooled down for duplex formation and decay of hemiacetals (Supporting Information File 1, Table S1). The samples were subsequently measured without additional treatment. Figure 3 shows an example for the PELDOR distance measurements conducted for all doubly labeled palindromes **22c**–**27c**. Like for all samples, **24c** shows a clear oscillation in the time trace and a well-defined distance. The results for all PELDOR measurements are shown in Figure S21 (Supporting Information File 1). The experimental distances are summarized in Table 1 and coincide well with values predicted from modelling. However, although the degree of spin labeling was very high in all cases, large variations in modulation depth are visible (Supporting Information File 1, Figure S21). This effect can be explained by conformational equilibria between palindromic dimers and monomeric hairpin structures lacking a second TEMPO label. Analysis by NUPACK [47] predicts RNA 18mer **27c** to exist exclusively in form of the duplex while DNA 18mers **23c** and **25c** should also form the monomeric hairpin structures. This prediction can be verified by native gel electrophoresis (Supporting Information File 1, Figure S19). Electrophoresis also shows the 12mer RNA **26c** to form mainly duplexes while high amounts of monomers are seen with DNA analogs **22c** and **24c**, in full accord with NUPACK predictions and the experimentally observed levels of modulation depth.

**Table 1:** Spin–spin distances in oligonucleotides **22c–27c** determined by PELDOR and by molecular modelling. The predicted distance is an average of N–N, N–O, O–N, and O–O distances.

sample	PELDOR	simulation

**22c**	2.5 ± 0.2 nm	2.6 nm
**24c**	2.9 ± 0.2 nm	3.0 nm
**26c**	2.5 ± 0.2 nm	2.5 nm

**23c**	4.1 ± 0.2 nm	4.3 nm
**25c**	4.9 ± 0.2 nm	5.0 nm
**27c**	4.1 ± 0.2 nm	4.0 nm

## Conclusion

All three phosphoramidite building blocks **5**, **7**, and **8** are applicable for automated solid-phase synthesis using standard reaction cycles. As in the case of amidite **6**, the 2-nitrobenzyloxymethyl group is stable and does not interfere with work-up and purification. Thus, all oligonucleotides **22a**–**27a** were obtained in good yield and high purity. While photochemical removal of the 2-nitrobenzyl part is fast, care has to be taken to complete the conversion of hemiacetals. After the annealing procedure, mean spin labeling efficiencies of 96% have been found. Samples of similar quality including TEMPO labeled cytidines are also accessible by postsynthetic modification of convertible nucleotides [8,25–26]. However, the analogous reaction forming TEMPO-labeled adenosine tends to be sluggish and incomplete [25]. Furthermore, the protection group present in **22a**–**27a** also shields the nitroxide precursor if subsequent enzymatic ligation steps in thiol containing buffers are required [43]. A single case has been reported earlier of an *O*-acetyl protected TEMPO moiety attached to a deoxyuridine phosphoramidite by a more flexible chain [49]. This building block also enabled the synthesis of spin labeled DNA strands but required incubation with 0.5 M aqueous NaOH for 24 h for complete release of the nitroxide. Such conditions are not compatible with RNA. Therefore, the use of photolabile protective groups for nitroxides currently provides the most general approach for synthesizing spin labeled DNA and RNA. This strategy looks promising not only for TEMPO but also for other types of nitroxides such as TPA [20–21,27,29] or Ç [31–33].

## Supporting Information

File 1 Synthesis, purification and photochemical deprotection of oligonucleotides, mass spectra and HPLC plots. ^1^H and ^13^C NMR spectra.
