# Supplementary material for: Phosphoramidite building blocks with protected nitroxides for the synthesis of spin-labeled DNA and RNA
Source: Beilstein J Org Chem. 2018 Jun 26;14:1563–9. doi: 10.3762/bjoc.14.133 (PMC6036967; doi:10.3762/bjoc.14.133)

Supporting Information

for

Phosphoramidite building blocks with protected nitroxides for the synthesis of spin-labeled DNA and RNA

Timo Weinrich[1], Eva A. Jaumann[2], Ute M. Scheffer[1], Thomas F. Prisner[2] and Michael W. Göbel*[1]

Address: [1]Institute of Organic Chemistry and Chemical Biology, Goethe University Frankfurt, Max-von-Laue-Str. 7, D-60438 Frankfurt am Main, Germany and [2]Institute of Physical and Theoretical Chemistry, Goethe University Frankfurt, Max-von-Laue-Str. 7, D-60438 Frankfurt am Main, Germany

Email: Michael W. Göbel* - m.goebel@chemie.uni-frankfurt.de

*Corresponding author

Synthesis, purification and photochemical deprotection of oligonucleotides, mass spectra and HPLC plots. ^{1}H and ^{13}C NMR spectra

Table of contents:

General information..S2

Synthesis of phosphoramidites **5**, **7**, and **8**...S2

Synthesis, purification, and quantification of oligonucleotides...S10

Photochemical deprotection of spin labeled DNAs and RNAs...................................... S15

EPR method part...S21

Spin-spin-distances in palindromic duplexes...S24

References..S25

Copies of ^{1}H and ^{13}C NMR spectra of new compounds..S26

General information

Compounds containing 2-nitrobenzyl groups should be handled in dim light only! Anhydrous pyridine, dichloromethane and methanol were purchased from Sigma-Aldrich. Flash column chromatography: silica gel (60 Å pore size, 0.04–0.063 mm particle size). Analytical thin layer chromatography: aluminum plates pre-coated with silica gel (0.2 mm, 60 Å pore size, Merck) impregnated with a fluorescent indicator (254 nm). TLC plates were visualized by exposure to ultraviolet light (UV). After purification via silica gel chromatograpgy every compound was lyophilized with benzene. Proton nuclear magnetic resonance (^{1}H NMR) spectra, carbon nuclear magnetic resonance (^{13}C NMR) and phosphorus nuclear magnetic resonance (^{31}P NMR) were recorded at 300 K with Bruker AV 300 (^{1}H: 300 MHz; ^{13}C: 75.5 MHz; ^{31}P: 121.5 MHz) or Bruker AV 500 (^{1}H: 500 MHz; ^{13}C: 125.8 MHz) NMR spectrometers. Chemical shifts for protons are reported in parts per million (δ scale) and internally referenced to the proton resonances of the solvent ($CDCl_3$: δ 7.26, DMSO-d_6: δ 2.50). Chemical shifts for carbon are reported in parts per million (δ scale) and referenced to the carbon resonances of the solvent ($CDCl_3$: δ 77.00, DMSO-d_6: δ 39.51). Data are represented as follows: chemical shift, multiplicity (s = singlet, bs = broad singlet, d = doublet, bd = broad doublet, dd = doublet of doublets, ddd = doublet of doublet of doublets t = triplet, dt = doublet of triplets, bt = broad triplet, q = quartet, quin = quintet, m = multiplet), coupling constants in Hz, and integration. ESIMS spectra were obtained on a Fisons VG Plattform II. HRMS spectra were recorded on a MALDI LTQ Orbitrap mass spectrometer from Thermo Scientific.

Synthesis of phosphoramidites

1-(3’-*O*-Acetyl-5’-*O*-DMT-2’-deoxyribofuranosyl)-4-(2,2,6,6-tetramethyl-1-((2-nitrobenzyl-oxy)methoxy)piperidin-4-ylamino)pyrimidin-2(1*H*)-one (11): A solution of 3´-*O*-acetyl-5´-*O*-DMT-deoxyuridine **9** [1] (4.86 g, 8.48 mmol, 1.00 equiv), 4-dimethylaminopyridine (0.16 g, 1.27 mmol, 0.15 equiv) and Et_3N (10.7 mL, 9.75 mmol, 9.00 equiv) in 80 mL CH_2Cl_2 was cooled to 0 °C, treated with 2,4,6-triisopropylbenzenesulfonyl chloride (2.95 g, 9.75 mmol, 1.15 equiv) and stirred for 10 min at 0 °C. The solution was allowed to warm up and was stirred for 19 h at ambient temperature. Subsequently, the reaction mixture was quenched with conc. $NaHCO_3$ solution, the organic layer was separated and the aqueous layer was extracted with CH_2Cl_2. The combined organic layers were dried with $MgSO_4$ and the solvent was evaporated under reduced pressure (waterbath 30 °C). The *O*-sulfonylated intermediate was resolved in 40 mL DMF and diisopropylethylamine (2.8 mL, 16.53 mmol, 2.60 equiv) and **10** [2] (2.79 g, 8.26 mmol, 1.30 equiv) was added. The reaction mixture was heated to 90 °C and stirred for 24 h at the same temperature. Afterwards the solvent was removed under reduced pressure (waterbath at 60 °C to remove DMF). Purification by silica gel

chromatography (EtOAc/Et_3N 100:1) gave nucleoside **11** as a colourless foam (5.18 g, 69%). R_f = 0.60 (EtOAc). ^{1}H-NMR (500 MHz, d_6-DMSO): 8.08 (d, *J* = 8.5 Hz, 1 H, Ar-H), 7.78-7.77 (m, 2 H, Ar-H), 7.59-7.55 (m, 3 H, Ar-H, N*H*, H-6), 7.36 (d, *J* = 7.0 Hz, 2 H, Ar-H), 7.31 (t, *J* = 7.6 Hz, 2 H, Ar-H), 7.25-7.22 (m, 5 H, Ar-H), 6.90-6.88 (m, 4 H, Ar-H), 6.17 (t, *J* = 7.1 Hz, 1 H, 1´H), 5.55 (d, *J* = 7.8 Hz, 1 H, H-5), 5.22-5.21 (m, 1 H, 3´H), 5.00 (s, 2 H, OCH_2O), 4.97 (s, 2 H, $ArCH_2O$), 4.24-4.17 (m, 1 H, C*H*NH), 4.06 (q, *J* = 4.5 Hz, 1 H, 4´H), 3.74 (s, 6 H, OCH_3), 3.31-3.29 (m, 1 H, 5´H), 3.22 (dd, *J* = 10.0, 3.0 Hz, 1 H, 5´´H), 2.34-2.22 (m, 2 H, 2´H, 2´´H), 2.04 (s, 3 H, $COCH_3$), 1.77 (d, *J* = 11.5 Hz, 2 H, CH*H*CH), 1.32 (t, *J* = 11.5 Hz, 2 H, C*H*HCH), 1.13-1.12 (m, 12 H, CH_3) ppm. ^{13}C-NMR: (125.8 MHz, d_6-DMSO): 170.0, 162.6, 158.1, 154.8, 147.3, 144.6, 139.5, 135.3, 135.2, 133.9, 133.7, 129.7, 128.9, 128.7, 127.9, 127.7, 126.8, 124.6, 113.2, 101.1, 94.9, 85.9, 84.9, 82.8, 74.2, 67.2, 63.4, 59.2, 55.1, 45.7, 44.7, 40.8, 37.2, 32.8, 20.8, 20.5 ppm. MS (ESI): *m/z* = 892.70 [M + H^+]. HRMS (MALDI): calcd. for $C_{49}H_{57}N_5O_{11}Na$ [M + Na^+]: 914.39468 found 914.39641.

1-(5’-*O*-DMT-2’-Deoxyribofuranosyl)-4-(2,2,6,6-tetramethyl-1-((2-nitrobenzyloxy)methoxy)-piperidin-4-ylamino)pyrimidin-2(1*H*)-one (12): Compound **11** (5.06 g, 5.67 mmol, 1.00 equiv) was dissolved in 100 mL MeOH and $NaHCO_3$ (5.95 g, 70.90 mmol, 12.50 equiv) was added. After stirring for 2.5 h at ambient temperature, 200 mL of a CH_2Cl_2/MeOH/Et_3N-mixture (96:4:1) was added and the reaction mixture was filtered through silica gel (silica gel was deactivated with Et_3N before). After evaporation of solvents under reduced pressure, **12** was obtained as a colourless foam (4.81 g, quant.). R_f = 0.14 (CH_2Cl_2/MeOH 19:1). ^{1}H-NMR (500 MHz, d_6-DMSO): 8.08 (d, *J* = 7.8 Hz, 1 H, Ar-H), 7.78-7.77 (m, 2 H, Ar-H), 7.60-7.57 (m, 2 H, Ar-H, N*H*), 7.52 (d, *J* = 7.5 Hz, 1 H, H-6), 7.38-7.36 (m, 2 H, Ar-H), 7.31 (t, *J* = 7.5 Hz, 2 H, Ar-H), 7.26-7.23 (m, 5 H, Ar-H), 6.90-6.88 (m, 4 H, Ar-H), 6.15 (t, *J* = 6.5 Hz, 1 H, 1´H), 5.52 (d, *J* = 7.5 Hz, 1 H, H-5), 5.28 (d, *J* = 4.5 Hz, 1 H, 3´H), 5.00 (s, 2 H, OCH_2O), 4.97 (s, 2 H, $ArCH_2O$), 4.26-4.17 (m, 2 H, C*H*NH, 3´-OH), 3.86 (q, *J* = 3.5 Hz, 1 H, 4´H), 3.74 (s, 6 H, OCH_3), 3.22-3.16 (m, 2 H, 5´H, 5´´H), 2.19-2.14 (m, 1 H, 2´H), 2.05-1.99 (m, 1 H, 2´´H), 1.77 (d, *J* = 10.0 Hz, 2 H, CH*H*CH), 1.32 (t, *J* = 12.5 Hz, 2 H, C*H*HCH), 1.13-1.12 (m, 12 H, CH_3) ppm. ^{13}C-NMR: (125.8 MHz, d_6-DMSO): 162.6, 158.1, 154.9, 147.3, 144.7, 139.5, 135.4, 135.3, 133.9, 133.7, 129.7, 128.9, 128.7, 127.9, 127.7, 126.7, 124.6, 113.2, 101.1, 94.5, 85.7, 85.1, 84.7, 70.0, 67.2, 63.4, 59.2, 55.0, 45.7, 44.7, 40.7, 40.4, 32.8, 20.5 ppm. MS (ESI): *m/z* = 850.64 [M + H^+]. HRMS (MALDI): calcd. for $C_{47}H_{56}N_5O_{10}$ [M + H^+]: 850.40217 found 850.40268.

Deoxycytidine phosphoramidite with protected spin label (5): To a solution of **12** (4.81 g, 5.67 mmol, 1.00 equiv) and Et_3N (4.0 mL, 28.29 mmol, 5.00 equiv) in 50 mL CH_2Cl_2 *N*,*N*-diisopropylamino(2-cyanoethyl)phosphoramidic chloride (2.68 g, 11.31 mmol, 2.00 equiv) was added. The reaction mixture was stirred for 20 h at ambient temperature. Subsequently conc. $NaHCO_3$ solution was added, the organic layer was separated and the aqueous layer was extracted with CH_2Cl_2. The combined organic layers were dried with $MgSO_4$ and the solvent was removed under reduced pressure. Purification by silica gel chromatography (EtOAc/Et_3N 100:1) **5** was obtained as a

colourless foam (4.83 g, 81%). R_f = 0.75, 0.60 (EtOAc (mixture of 2 diasteromers)). ^{1}H-NMR (300 MHz, d_6-DMSO): 8.08 (d, J = 8.7 Hz, 1 H, Ar-H), 7.78-7.77 (m, 2 H, Ar-H), 7.64-7.52 (m, 3 H, Ar-H, H-6, N*H*), 7.40-7.36 (m, 2 H, Ar-H), 7.34-7.22 (m, 7 H, Ar-H), 6.90-6.85 (m, 4 H, Ar-H), 6.19-6.13 (m, 1 H, 1´H), 5.56-5.53 (m, 1 H, H-5), 5.00 (s, 2 H, OCH_2O), 4.97 (s, 2 H, $ArCH_2O$), 4.52-4.44 (m, 1 H, C*H*NH), 4.26-4.15 (m, 1 H, 3´H), 4.02-3.96 (m, 1 H, 4´H), 3.74, 3.73 (2 x s, 6 H, OCH_3), 3.67-3.43 (m, 4 H, $POCH_2$, $NCH(CH_3)_2$), 3.29-3.19 (m, 2 H, 5´H, 5´´H), 2.75 (t, J = 5.7 Hz, 1 H, CH*H*CN), 2.64 (t, J = 6.3 Hz, 1 H, C*H*HCN), 2.36-2.15 (m, 2 H, 2´H, 2´´H), 1.77 (dd, J = 12.6, 3.0 Hz, 2 H, CH*H*CH), 1.32 (t, J = 12.6 Hz, 2 H, C*H*HCH), 1.15-1.08 (m, 21 H, $NCH(CH_3)_2$, CH_3), 0.98 (d, J = 6.9 Hz, 3 H, $NCH(CH_3)_2$) ppm. (mixture of 2 diastereomers). ^{13}C-NMR (75.5 MHz, d_6-DMSO): 162.6, 158.1, 154.8, 147.3, 144.6, 139.8, 139.5, 135.3, 135.2, 133.8, 133.7, 129.7, 128.9, 128.6, 127.8, 127.7, 126.8, 124.5, 118.9, 118.7, 113.2, 101.1, 94.74, 94.65, 85.9, 85.8, 84.9, 72.9, 72.6, 67.2, 59.2, 58.5, 58.3, 58.2, 58.1, 55.0, 44.7, 42.6, 42.5, 40.8, 32.8, 24.4, 24.3, 24.2, 24.1, 20.5, 19.81, 19.76, 19.72, 19.67, 14.0 ppm. ^{31}P-NMR (121.5 MHz, d_6-DMSO): 147.7, 147.3 ppm. MS (ESI): *m/z* = 1050.88 [M + H$^+$]; calcd. for $C_{56}H_{73}N_7O_{11}P$ [M + H$^+$]: 1050.51.

9-(3’,5’-Di-*O*-acetyl-2’-deoxyribofuranosyl)-6-chloropurine (13): 3´,5´-Di-*O*-acetyldeoxyinosine [3] (1.52 g, 4.52 mmol, 1.00 equiv), benzyltriethylammonium chloride (2.06 g, 9.04 mmol, 2.00 equiv) and *N,N*-dimethylaniline (0.6 mL, 4.97 mmol, 1.10 equiv) were dissolved in 18 mL dry acetonitrile. The flask was placed on a preheated oil bath (70 °C), $POCl_3$ (2.1 mL, 22.60 mmol, 5.00 equiv) was added slowly and the reaction mixture was stirred for 1 h at the same temperature. After that, the solvent and excess $POCl_3$ were removed under reduced pressure (high vacuum, 70 °C). The residue was poured on a $CHCl_3$/ice-mixture and the solution was stirred for 20 min. The organic phase was separated and the aqueous phase was extracted 3 times with $CHCl_3$. Organic phases were combined and washed with a 5% $NaHCO_3$ solution until the aqueous layer showed a slightly basic reaction. Subsequently the organic phase was separated, dried with $MgSO_4$ and the solvent was removed under reduced pressure. Purification by silica gel chromatography (EtOAc) gave compound **13** as a yellow oil (1.39 g, 87%). R_f = 0.49 (EtOAc). ^{1}H-NMR (500 MHz, d_6-DMSO): 8.88 (s, 1 H, H-2), 8.80 (s, 1 H, H-8), 6.51 (t, J = 7.0 Hz, 1 H, 1´H), 5.46-5.44 (m, 1 H, 3´H), 4.33-4.29 (m, 2 H, 4´H, 5´H), 4.23 (dd, J = 12.5, 7.7 Hz, 1 H, 5´´H), 3.19 (quin, J = 7.0 Hz,1 H, 2´H), 2.63 (ddd, J = 14.3, 6.4, 3.0 Hz, 1 H, 2´´H), 2.10 (s, 3 H, $COCH_3$), 1.99 (s, 3 H, $COCH_3$) ppm. ^{13}C-NMR (125.8 MHz, d_6-DMSO): 170.10, 170.05, 151.7, 151.4, 149.4, 146.0, 131.5, 84.3, 82.0, 74.1, 63.4, 35.5, 20.8, 20.5 ppm. MS (ESI): *m/z* = 355.14 [M + H$^+$]. HRMS (MALDI): calcd. for $C_{14}H_{16}ClN_4O_5$ [M + H$^+$]: 355.08037 found 355.08062.

9-(3’,5’-Di-*O*-acetyl-2’-deoxyribofuranosyl)-6-(2,2,6,6-tetramethyl-1-((2-nitrobenzyloxy)-methoxy)piperidin-4-ylamino)purine (14): Compound **13** (2.40 g, 6.76 mmol, 1.00 equiv) and diisopropylethylamine (2.3 mL, 13.53 mmol, 2.00 equiv) were dissolved in 40 mL 1-propanol. After that **10** [2] (2.51 g, 7.44 mmol, 1.10 equiv) was added and the reaction mixture was stirred for 8 h at

75 °C, cooled down to ambient temperature and stirred for another 14 h. The reaction was quenched with conc. $NaHCO_3$ solution. After extraction with CH_2Cl_2, the combined organic layers were dried with $MgSO_4$ and the solvent was removed under reduced pressure. Purification by silica gel chromatography (CH_2Cl_2/EtOAc 9:1 → 0:1) gave title compound **14** as a light yellow foam (2.96 g, 67%). R_f = 0.16 (CH_2Cl_2/EtOAc 1:1). ^{1}H-NMR (500 MHz, d_6-DMSO): 8.38 (bs, 0.30 H, H-2), 8.35 (bs, 0.70 H, H-2), 8.25 (bs, 0.70 H, H-8), 8.12 (bs, 0.30 H, H-8), 8.08 (d, *J* = 8.0 Hz, 1 H, Ar-H), 7.78 (d, *J* = 4.5 Hz, 2 H, Ar-H), 7.70-7.64 (m, 0.70 H, N*H*), 7.62-7.56 (m, 1.30 H, Ar-H, N*H*), 6.37 (dd, *J* = 8.5, 6.2 Hz, 1 H, 1´H), 5.41-5.38 (m, 1 H, 3´H), 5.23 (bs, 0.30 H, C*H*NH), 5.00 (s, 2 H, OC*H₂*O), 4.98 (s, 2 H, ArC*H₂*O), 4.57 (bs, 0.70 H, C*H*NH), 4.31 (dd, *J* = 10.8, 3.8 Hz, 1 H, 5´H), 4.25-4.18 (m, 2 H, 4´H, 5´H), 3.21-3.11 (m, 1 H, 2´H), 2.53 (dd, *J* = 6.3, 2.5 Hz, 1 H, 2´´H), 2.09 (s, 3 H, COC*H₃*), 2.01 (s, 3 H, COC*H₃*), 1.74 (d, *J* = 11.0 Hz, 2 H, CH*H*CH), 1.66-1.53 (m, 2 H, C*H*HCH), 1.17 (s, 6 H, C*H₃*), 1.12 (s, 6 H, C*H₃*) ppm (mixture of 2 rotamers at CN-bonds). ^{13}C-NMR (125.8 MHz, d_6-DMSO): 170.14, 170.05, 152.8, 148.5, 147.2, 139.3, 133.9, 133.8, 128.9, 128.7, 124.6, 101.0, 83.5, 81.6, 74.4, 67.2, 63.6, 59.4, 44.5, 40.8, 35.2, 32.8, 20.8, 20.6, 20.5 ppm. MS (ESI): *m/z* = 656.52 [M + H^+]. HRMS (MALDI): calcd. for $C_{31}H_{42}N_7O_9$ [M + H^+]: 656.30385 found 656.30339.

9-(2’-Deoxyribofuranosyl)-6-(2,2,6,6-tetramethyl-1-((2-nitrobenzyloxy)-methoxy)piperidin-4-ylamino)purine (15): Compound **14** (3.12 g, 4.76 mmol, 1.00 equiv) was dissolved in 50 mL MeOH at 0 °C and 7 N NH_3 in MeOH (35 mL) was added. After stirring for 15 min at 0 °C the reaction mixture was allowed to warm up to ambient temperature and stirred for another 3 h. Afterwards the solution was cooled down again to 0 °C and neutralized with an ice-cold 6 M HCl-solution. Subsequently conc. $NaHCO_3$ solution was added and the mixture was extracted with CH_2Cl_2. The combined organic layers were dried with $MgSO_4$ and the solvent was evaporated under reduced pressure. After purification by silica gel chromatography (CH_2Cl_2/MeOH 9:1) **15** could be obtained as a light yellow foam (2.45 g, 90%). R_f = 0.46 (CH_2Cl_2/MeOH 9:1). ^{1}H-NMR (500 MHz, d_6-DMSO): 8.37 (bs, 0.30 H, H-2), 8.34 (bs, 0.70 H, H-2), 8.22 (bs, 0.70 H, H-8), 8.14 (bs, 0.30 H, H-8), 8.08 (d, *J* = 8.2 Hz, 1 H, Ar-H), 7.78 (d, *J* = 4.5 Hz, 2 H, Ar-H), 7.70-7.63 (m, 0.70 H, N*H*), 7.61-7.56 (m, 1.30 H, Ar-H, N*H*), 6.34 (dd, *J* = 8.5, 6.5 Hz, 1 H, 1´H), 5.43-5.05 (m, 2.30 H, 3´H, 5´-OH, C*H*NH), 5.00 (s, 2 H, OC*H₂*O), 4.98 (s, 2 H, ArC*H₂*O), 4.56 (bs, 0.70 H, C*H*NH), 4.42-4.38 (m, 1 H, 3´-OH), 3.89-3.86 (m, 1 H, 4´H), 3.62 (dd, *J* = 11.8, 4.2 Hz, 1 H, 5´H) 3.51 (dd, *J* = 11.8, 4.3 Hz, 1 H, 5´´H), 2.75-2.66 (m, 1 H, 2´H), 2.25 (ddd, *J* = 13.0, 6.5, 2.5 Hz, 1 H, 2´´H), 1.74 (d, *J* = 10.5 Hz, 2 H, CH*H*CH), 1.66-1.51 (m, 2 H, C*H*HCH), 1.17 (s, 6 H, C*H₃*), 1.12 (s, 6 H, C*H₃*) ppm (mixture of 2 rotamers at CN-bonds). ^{13}C-NMR (125.8 MHz, d_6-DMSO): 154.0, 152.5, 148.2, 147.2, 139.3, 133.9, 133.8, 128.9, 128.7, 124.6, 119.6, 101.1, 88.0, 83.9, 70.9, 67.2, 61.9, 59.4, 44.5, 40.8, 32.8, 20.6 ppm. MS (ESI): *m/z* = 572.30 [M + H^+]. HRMS (MALDI): calcd. for $C_{27}H_{38}N_7O_7$ [M + H^+]: 572.28272 found 572.28157.

9-(5'-*O*-DMT-2'-Deoxyribofuranosyl)-6-(2,2,6,6-tetramethyl-1-((2-nitrobenzyloxy)-methoxy)piperidin-4-ylamino)purine (16): To an ice-cold solution of **15** (2.36 g, 4.12 mmol, 1.00 equiv) in 100 mL dry pyridine dimethoxytrityl chloride (1.67 g, 4.94 mmol, 1.20 equiv) was added. After the reaction mixture was allowed to warm up to ambient temperature, it was stirred for 23 h and cooled down to 0 °C again. The reaction was quenched with MeOH and stirred for 15 min at 0 °C. Subsequently the solvent was removed under reduced pressure and the residue was coevaporated with toluene. Purification by silica gel chromatography (1st CH_2Cl_2/MeOH/Et_3N 96:4:1; 2nd EtOAc/MeOH 100:0 → 90:10) gave title compound **16** as a light yellow foam (2.40 g, 67%). R_f = 0.38 (EtOAc). ^{1}H-NMR (500 MHz, d_6-DMSO): 8.25 (s, 1 H, H-2), 8.17 (bs, 0.70 H, H-8), 8.08 (d, *J* = 8.5 Hz, 1 H, Ar-H), 8.06 (bs, 0.30 H, H-8), 7.78 (d, *J* = 4.0 Hz, 2 H, Ar-H), 7.64-7.51 (m, 2 H, Ar-H, N*H*), 7.33-7.31 (m, 2 H, Ar-H), 7.23-7.17 (m, 7 H, Ar-H), 6.81-6.76 (m, 4 H, Ar-H), 6.36 (t, *J* = 6.5 Hz, 1 H, 1´H), 5.35 (d, *J* = 4.5 Hz, 1 H, 3´H), 5.21 (bs, 0.30 H, C*H*NH), 5.00 (s, 2 H, OCH_2O), 4.98 (s, 2 H, ArCH_2O), 4.56 (bs, 0.70 H, C*H*NH), 4.50 (bs, 1 H, 3´-OH), 3.98-3.95 (m, 1 H, 4´H), 3.715 (s, 3 H, OCH_3), 3.705 (s, 3 H, OCH_3), 3.19-3.12 (m, 2 H, 5´H, 5´´H), 2.93-2.81 (m, 1 H, 2´H), 2.35-2.30 (m, 1 H, 2´´H), 1.74 (d, *J* = 10.5 Hz, 2 H, CH*H*CH), 1.66-1.50 (m, 2 H, C*H*HCH), 1.17 (s, 6 H, CH_3), 1.12 (s, 6 H, CH_3) ppm (mixture of 2 rotamers at CN-bonds). ^{13}C-NMR (125.8 MHz, d_6-DMSO): 157.98, 157.95, 153.9, 152.6, 147.2, 144.9, 139.2, 135.6, 135.5, 133.9, 133.8, 129.7, 129.6, 128.9, 128.7, 127.71, 127.65, 126.5, 124.6, 113.05, 113.03, 101.1, 85.8, 85.4, 70.6, 67.2, 64.0, 59.4, 55.0, 44.5, 38.7, 32.8, 20.6 ppm. MS (ESI): *m/z* = 874.46 [M + H^+]. HRMS (MALDI): calcd. for $C_{48}H_{55}N_7O_9K$ [M + K^+]: 912.36928 found 912.37126.

Deoxyadenosine phosphoramidite with protected spin label (7): To a solution of **16** (2.03 g, 2.32 mmol, 1.00 equiv) and Et_3N (1.6 mL, 11.61 mmol, 5.00 equiv) in 40 mL CH_2Cl_2 *N*,*N*-diisopropylamino(2-cyanoethyl)phosphoramidic chloride (1.10 g, 4.65 mmol, 2.00 equiv) was added dropwise. After stirring for 4.5 h at ambient temperature, conc. $NaHCO_3$ solution was added and the organic layer was separated. The aqueous layer was extracted with CH_2Cl_2 and the combined organic layers were dried with $MgSO_4$. The solvent was removed under reduced pressure. Purification by silica gel chromatography (CH_2Cl_2/EtOAc/Et_3N 50:50:1) gave amidite **7** as a colourless foam (2.02 g, 81%). R_f = 0.82, 0.70 (CH_2Cl_2/EtOAc 1:1 (mixture of 2 diastereomers)). ^{1}H-NMR (500 MHz, d_6-DMSO): 8.27 (s, 1 H, H-2), 8.15 (bs, 0.70 H, H-8), 8.08 (d, *J* = 8.2 Hz, 1 H, Ar-H), 8.04 (bs, 0.30 H, H-8), 7.81-7.75 (m, 2 H, Ar-H), 7.67-7.52 (m, 2 H, Ar-H, N*H*), 7.34-7.29 (m, 2 H, Ar-H), 7.24-7.17 (m, 7 H, Ar-H), 6.81-6.74 (m, 4 H, Ar-H), 6.42-6.34 (m, 1 H, 1´H), 5.20 (bs, 0.30 H, C*H*NH), 5.00 (s, 2 H, OCH_2O), 4.98 (s, 2 H, ArCH_2O), 4.80 (bs, 1 H, 3´H), 4.56 (bs, 0.70 H, C*H*NH), 4.15-4.06 (m, 1 H, 4´H), 3.80-3.73 (m, 1 H, POCH*H*), 3.71 (s, 3 H, OCH_3), 3.70 (s, 3 H, OCH_3), 3.67-3.63 (m, 1 H, POC*H*H), 3.59-3.50 (m, 2 H, NC*H*(CH_3)$_2$), 3.27-3.17 (m, 2 H, 5´H, 5´´H), 3.13-3.02 (m, 1 H, 2´H), 2.77 (t, *J* = 6.0 Hz, 1 H, CH*H*CN), 2.66 (t, *J* = 6.0 Hz, 1 H, C*H*HCN), 1.74 (d, *J* = 10.5 Hz, 2 H, CH*H*CH), 1.66-1.50 (m, 2 H, C*H*HCH), 1.18-1.07 (m, 21 H, NCH(CH_3)$_2$, CH_3), 1.03 (d, *J* = 6.8 Hz, 3

H, NCH(C*H*$_3$)$_2$) ppm (mixture of 2 rotamers at CN-bonds and 2 diastereomers). ^{13}C-NMR (125.8 MHz, d$_6$-DMSO): 157.98, 157.95, 153.9, 152.6, 148.3, 147.2, 144.8, 139.5, 135.6, 135.5, 133.9, 133.8, 129.7, 129.6, 128.9, 128.7, 127.71, 127.65, 126.5, 124.6, 119.6, 119.0, 118.8, 113.03, 113.0, 101.0, 85.5, 84.7, 84.5, 83.5, 73.3, 73.2, 72.7, 67.2, 63.4, 63.3, 59.4, 58.4, 55.0, 44.5, 42.6, 42.5, 40.7, 37.5, 37.2, 32.8, 24.4, 24.3, 24.2, 24.1, 20.6, 19.8, 19.7 ppm. ^{31}P-NMR (202.5 MHz, d$_6$-DMSO): 147.6, 147.0 ppm. MS (ESI): *m/z* = 1074.69 [M + H$^+$]; calcd. for $C_{57}H_{73}N_9O_{10}P$ [M + H$^+$]: 1074.52.

9-(2’,3’,5’-Tri-*O*-acetylribofuranosyl)-6-chloropurine (17): 2´,3´,5´-Tri-*O*-acetylinosine [4] (5.00 g, 12.68 mmol, 1.00 equiv), benzyltriethylammonium chloride (5.77 g, 25.36 mmol, 2.00 equiv) and *N*,*N*-dimethylaniline (1.8 mL, 13.94 mmol, 1.10 equiv) were dissolved in 50 mL dry acetonitrile. The flask was placed on a preheated oil bath (70 °C), $POCl_3$ (5.9 mL, 63.40 mmol, 5.00 equiv) was added slowly and the reaction mixture was stirred for 2 h at the same temperature. After that, the solvent and excess $POCl_3$ were removed under reduced pressure (high vacuum, 70 °C). The residue was poured on a $CHCl_3$/ice-mixture and the solution was stirred for 20 min. The organic phase was separated and the aqueous phase was extracted 3 times with $CHCl_3$. The organic phases were combined and washed with a 5 % $NaHCO_3$ solution until the aqueous layer showed a slightly basic reaction. Subsequently the organic phase was separated, dried with $MgSO_4$ and the solvent was removed under reduced pressure. Purification by silica gel chromatography (EtOAc) gave compound **17** as a yellow oil (5.23 g, quant.). R_f = 0.65 (EtOAc). ^{1}H-NMR (500 MHz, d$_6$-DMSO): 8.90 (s, 1 H, H-2), 8.85 (s, 1 H, H-8), 6.37 (d, *J* = 5.0 Hz, 1 H, 1´H), 6.03 (t, *J* = 5.5 Hz, 1 H, 2´H), 5.65 (t, *J* = 5.4 Hz, 1 H, 3´H), 4.45-4.40 (m, 2 H, 4´H, 5´H), 4.30-4.26 (m, 1 H, 5´´H), 2.12 (s, 3 H, COC*H*$_3$), 2.04 (s, 3 H, COC*H*$_3$), 2.01 (s, 3 H, COC*H*$_3$) ppm. ^{13}C-NMR (125.8 MHz, d$_6$-DMSO): 170.0, 169.4, 169.3, 152.0, 151.3, 149.7, 146.4, 131.6, 86.2, 79.7, 72.1, 69.9, 62.7, 20.5, 20.4, 20.2 ppm. MS (ESI): *m/z* = 413.11 [M + H$^+$]. HRMS (MALDI): calcd. for $C_{16}H_{17}ClN_4O_7Na$ [M + Na$^+$]: 435.06780 found 435.06727.

9-(2’,3’,5’-Tri-*O*-acetylribofuranosyl)-6-(2,2,6,6-tetramethyl-1-((2-nitrobenzyloxy)methoxy)-piperidin-4-ylamino)purine (18): A solution of **17** (0.55 g, 1.33 mmol, 1.00 equiv), diisopropylethylamine (0.5 mL, 2.93 mmol, 2.20 equiv) and **10** [2] (0.59 g, 1.74 mmol, 1.30 equiv) in 1-propanol was stirred for 7 h at 75 °C. The solution was cooled down to ambient temperature, stirred for another 14 h and the solvent was removed under reduced pressure. Purification by silica gel chromatography (EtOAc) gave the title compound **18** as a light yellow foam (0.74 g, 78%). R_f = 0.56 (EtOAc). ^{1}H-NMR (500 MHz, d$_6$-DMSO): 8.36 (bs, 1 H, H-2), 8.26 (bs, 0.70 H, H-8), 8.14 (bs, 0.30 H, H-8), 8.08 (d, *J* = 8.0 Hz, 1 H, Ar-H), 7.78 (d, *J* = 4.5 Hz, 2 H, Ar-H), 7.75-7.65 (m, 1 H, N*H*), 7.60-7.56 (m, 1 H, Ar-H), 6.21 (d, *J* = 5.8 Hz, 1 H, 1´H), 6.03 (t, *J* = 5.8 Hz, 1 H, 2´H), 5.62 (t, *J* = 5.0 Hz, 1 H, 3´H), 5.19 (bs, 0.30 H, C*H*NH), 5.00 (s, 2 H, OC*H*$_2$O), 4.98 (s, 2 H, ArC*H*$_2$O), 4.57 (bs, 0.70 H, C*H*NH), 4.41 (dd, *J* = 12.5, 3.0 Hz, 1 H, 5´H), 4.36 (q, *J* = 4.8 Hz, 1 H, 4´H), 4.24 (dd, *J* = 12.0, 5.5 Hz, 1 H, 5´´H), 2.12 (s, 3 H, COC*H*$_3$), 2.03 (s, 3 H, COC*H*$_3$), 2.01 (s, 3 H, COC*H*$_3$), 1.81-1.72 (m, 2 H, CH*H*CH), 1.67-1.53 (m, 2 H, C*H*HCH), 1.17 (s, 6 H, C*H*$_3$), 1.12 (s, 6 H, C*H*$_3$) ppm (mixture of 2

rotamers at CN-bonds). ^{13}C-NMR (125.8 MHz, d_6-DMSO): 170.0, 169.5, 169.3, 154.0, 153.0, 148.4, 147.2, 139.8, 133.9, 133.8, 128.9, 128.7, 124.6, 119.5, 101.1, 85.6, 79.4, 71.9, 70.1, 67.2, 62.8, 59.4, 44.5, 40.8, 32.8, 20.6, 20.5, 20.4, 20.2 ppm. MS (ESI): *m/z* = 714.47 [M + H$^+$]. HRMS (MALDI): calcd. for $C_{33}H_{44}N_7O_{11}$ [M + H$^+$]: 714.30933 found 714.30886.

9-(Ribofuranosyl)-6-(2,2,6,6-tetramethyl-1-((2-nitrobenzyloxy)methoxy)piperidin-4-ylamino)-purine (19): Compound **18** (1.54 g, 2.15 mmol, 1.00 equiv) was dissolved in 45 mL 7 N NH_3 in MeOH at 0 °C. After stirring for 10 min the reaction mixture was allowed to warm up to ambient temperature and stirred for another 3 h. Subsequently the reaction mixture was neutralized with an ice-cold 6 M HCl solution. After adding conc. $NaHCO_3$ solution, the reaction mixture was extracted with CH_2Cl_2. The combined organic layers were dried with $MgSO_4$ whereupon the solvent was removed under reduced pressure. After purification by silica gel chromatography (CH_2Cl_2/MeOH 9:1) **19** was obtained as a light yellow foam (1.26 g, 93%). R_f = 0.36 (CH_2Cl_2/MeOH 9:1). ^{1}H-NMR (300 MHz, d_6-DMSO): 8.36 (bs, 1 H, H-2), 8.22 (bs, 1 H, H-8), 8.08 (d, *J* = 7.8 Hz, 1 H, Ar-H), 7.78 (d, *J* = 4.1 Hz, 2 H, Ar-H), 7.70-7.54 (m, 2 H, Ar-H, N*H*), 5.89 (d, *J* = 6.0 Hz, 1 H, 1´H), 5.42 (d, *J* = 6.3 Hz, 1 H, 2´-OH), 5.36 (dd, *J* = 6.8, 4.8 Hz, 1 H, 5´-OH), 5.25 (bs, 0.30 H, C*H*NH), 5.18 (d, *J* = 4.7 Hz, 1 H, 3´-OH), 5.00 (s, 2 H, OC*H₂*O), 4.98 (s, 2 H, ArC*H₂*O), 4.65-4.47 (q, bs, *J* = 6.3 Hz, 1.70 H, 2´H, C*H*NH), 4.17-4.12 (m, 1 H, 3´H), 3.96 (q, *J* = 3.3 Hz, 1 H, 4´H), 3.67 (dt, *J* = 12.0, 3.9 Hz, 1 H, 5´H), 3.59-3.50 (m, 1 H, 5´´H), 1.76 (bd, *J* = 12.8 Hz, 2 H, CH*H*CH), 1.63 (bt, *J* = 10.8 Hz, 2 H, C*H*HCH), 1.18 (s, 6 H, C*H₃*), 1.13 (s, 6 H, C*H₃*) ppm (mixture of 2 rotamers at CN-bonds). ^{13}C-NMR (75.5 MHz, d_6-DMSO): 154.0, 152.5, 148.4, 147.2, 139.6, 133.9, 133.8, 128.9, 128.7, 124.6, 119.7, 101.1, 87.9, 85.8, 73.5, 70.6, 67.2, 61.6, 59.4, 45.6, 44.5, 40.8, 32.8, 20.6 pm. MS (ESI): *m/z* = 588.37 [M + H$^+$]. HRMS (MALDI): calcd. for $C_{37}H_{38}N_7O_8$ [M + H$^+$]: 588.27764 found 588.27681.

9-(5'-*O*-DMT-Ribofuranosyl)-6-(2,2,6,6-tetramethyl-1-((2-nitrobenzyloxy)methoxy)piperidin-4-ylamino)purine (20): To a solution of **19** (1.13 g, 1.92 mmol, 1.00 equiv) in 50 mL dry pyridine, dimethoxytrityl chloride (0.78 g, 2.30 mmol, 1.20 equiv) was added at 0 °C. After stirring for 10 min at 0 °C the reaction mixture was allowed to warm up at ambient temperature and was stirred for another 22 h. The reaction was quenched with MeOH at 0 °C, stirred for 10 min at the same temperature whereupon the solvent was removed under reduced pressure. After coevaporation with toluene the residue was purified by silica gel chromatography (1st CH_2Cl_2/MeOH/Et_3N 96:4:1; 2nd EtOAc/MeOH/Et_3N 100:0:1 → 90:10:1). Title compound **20** was obtained as a light yellow foam (1.43 g, 84%). R_f = 0.46 (CH_2Cl_2/MeOH 9:1). ^{1}H-NMR (500 MHz, d_6-DMSO): 8.26 (bs, 1 H, H-2), 8.20 (bs, 1 H, H-8), 8.08 (d, *J* = 8.5 Hz, 1 H, Ar-H), 7.78 (d, *J* = 4.0 Hz, 2 H, Ar-H), 7.68-7.54 (m, 2 H, Ar-H, N*H*), 7.35-7.33 (m, 2 H, Ar-H), 7.26-7.17 (m, 7 H, Ar-H), 6.83-6.78 (m, 4 H, Ar-H), 5.93 (d, *J* = 4.0 Hz, 1 H, 1´H), 5.53 (d, *J* = 6.0 Hz, 1 H, 2´-OH), 5.25-5.17 (bs, d, *J* = 5.5 Hz, 1.30 H, C*H*NH, 3´-OH), 5.01 (s, 2 H, OC*H₂*O), 4.98 (s, 2 H, ArC*H₂*O), 4.73-4.21 (m, 1.70 H, 2´H, C*H*NH), 4.37-4.27 (m, 1 H, 3´H), 4.05 (q, *J* = 5.0 Hz, 1 H, 4´H), 3.72, 3.71 (2 x s, 6 H, OC*H₃*), 3.23-3.15 (m, 2 H, 5´H,

5´´H), 1.75 (bd, *J* = 10.5 Hz, 2 H, CH*H*CH), 1.67-1.52 (m, 2 H, C*H*HCH), 1.17 (s, 6 H, CH_3), 1.12 (s, 6 H, CH_3) ppm (mixture of 2 rotamers at CN-bonds). ^{13}C-NMR (125.8 MHz, d_6-DMSO): 158.01, 157.99, 152.7, 148.6, 147.2, 144.9, 139.4, 135.6, 135.5, 133.9, 133.8, 129.7, 128.9, 128.7, 127.8, 127.7, 126.6, 124.6, 119.5, 113.1, 101.0, 88.0, 85.4, 82.9, 73.1, 70.3, 67.2, 63.7, 59.4, 55.0, 44.5, 32.8, 20.7 ppm. MS (ESI): *m/z* = 890.61 [M + H^+]. HRMS (MALDI): calcd. for $C_{48}H_{55}N_7O_{10}Na$ [M + Na^+]: 912.39026 found 912.39168.

9-(2’-*O*-TBS-5’-*O*-DMT-Ribofuranosyl)-6-(2,2,6,6-tetramethyl-1-((2-nitrobenzyloxy)-methoxy)piperidin-4-ylamino)purine (21): Compound **20** (0.57 g, 0.64 mmol, 1.00 equiv) was dissolved in 15 mL DMF. After that, imidazole (0.35 g, 5.12 mmol, 8.00 equiv) and *tert*-butyldimethylsilyl chloride (0.17 g, 1.15 mmol, 1.80 equiv) were added and the reaction mixture was stirred for 20 h at ambient temperature. Conc. $NaHCO_3$ solution was added and the solution was extracted with CH_2Cl_2. The combined organic layers were dried with $MgSO_4$ and the solvent was removed under reduced pressure. Purification by silica gel chromatography (CH_2Cl_2/EtOAc/Et_3N 30:70:1) gave the title compound **21** as a colourless foam (0.20 g, 35%) (The 3´-*O*-TBDMS regioisomer and the bisilylated product could be deprotected with 1 M tetrabutylammonium fluoride solution (THF) and used again for the reaction after a silica gel chromatography (CH_2Cl_2/MeOH/Et_3N 90:10:1)). R_f = 0.82 (CH_2Cl_2/EtOAc 3:7). ^{1}H-NMR (500 MHz, d_6-DMSO): 8.27 (bs, 1 H, H-2), 8.17 (bs, 1 H, H-8), 8.08 (d, *J* = 8.0 Hz, 1 H, Ar-H), 7.78 (d, *J* = 4.5 Hz, 2 H, Ar-H), 7.70-7.52 (m, 2 H, Ar-H, N*H*), 7.38-7.37 (m, 2 H, Ar-H), 7.28-7.19 (m, 7 H, Ar-H), 6.85-6.82 (m, 4 H, Ar-H), 5.94 (d, *J* = 5.0 Hz, 1 H, 1´H), 5.25-5.10 (bs, d, *J* = 5.5 Hz, 1.30 H, C*H*NH, 3´-OH), 5.00 (s, 2 H, OCH_2O), 4.98 (s, 2 H, ArCH_2O), 4.87-4.75 (m, 1 H, 2´H), 4.61-4.51 (m, 0.70 H, C*H*NH), 4.31-4.21 (m, 1 H, 3´H), 4.08 (q, *J* = 4.5 Hz, 1 H, 4´H), 3.72 (s, 6 H, OCH_3), 3.27-3.22 (m, 2 H, 5´H, 5´´H), 1.75 (bd, *J* = 10.5 Hz, 2 H, CH*H*CH), 1.67-1.55 (m, 2 H, C*H*HCH), 1.17 (s, 6 H, CH_3), 1.12 (s, 6 H, CH_3), 0.76 (s, 9 H, SiC(CH_3)$_3$), (-0.03) – (-0.04) (m, 3 H, SiCH_3), (-0.13) (s, 3 H, SiCH_3) ppm (mixture of 2 rotamers at CN-bonds). ^{13}C-NMR (125.8 MHz, d_6-DMSO): 158.0, 153.9, 152.8, 148.6, 147.2, 144.9, 139.3, 135.5, 135.4, 133.9, 133.8, 129.7, 128.9, 128.7, 127.8, 127.7, 126.6, 124.6, 119.4, 113.1, 101.0, 87.9, 85.5, 83.2, 75.0, 70.2, 67.2, 63.4, 59.4, 55.0, 44.6, 40.8, 32.8, 25.8, 25.6, 25.5, 20.6, 17.9, -3.2, -4.8, -5.2 ppm. MS (ESI): *m/z* = 1004.70 [M + H^+]. HRMS (MALDI): calcd. for $C_{54}H_{70}N_7O_{10}Si$ [M + H^+]: 1004.49479 found 1004.49743.

Adenosine phosphoramidite with protected spin label (8): To a solution of **21** (0.54 g, 0.53 mmol, 1.00 equiv) and Et_3N (0.4 mL, 2.68 mmol, 5.00 equiv) in 20 mL CH_2Cl_2 *N,N*-diisopropylamino(2-cyanoethyl)phosphoramidic chloride (0.25 g, 1.07 mmol, 2.00 equiv) was added dropwise. After stirring for 23 h at ambient temperature conc. $NaHCO_3$ solution was added, the organic layer was separated and the aqueous phase was extracted with CH_2Cl_2. The combined organic layers were dried with $MgSO_4$ and the solvent was removed under reduced pressure. Purification by silica gel chromatography (CH_2Cl_2/EtOAc/Et_3N 80:20:1) gave amidite **8** as a colourless foam (0.54 g, 86%). R_f

= 0.85, 0.66 (CH_2Cl_2/EtOAc 4:1 (mixture of 2 diastereomers)). ^{1}H-NMR (300 MHz, d_6-DMSO): 8.32-8.26 (m, 1 H, H-2), 8.18-8.02 (m, 1 H, H-8), 8.08 (d, *J* = 8.7 Hz, 1 H, Ar-H), 7.80-7.76 (m, 2 H, Ar-H), 7.68-7.53 (m, 2 H, Ar-H, N*H*), 7.42-7.37 (m, 2 H, Ar-H), 7.30-7.20 (m, 7 H, Ar-H), 6.86-6.82 (m, 4 H, Ar-H), 5.96-5.88 (m, 1 H, 1´H), 5.17-5.09 (m, 1 H, 2´H), 5.00 (s, 2 H, OCH_2O), 4.98 (s, 2 H, ArCH_2O), 4.69-4.34 (m, 1.70 H, C*H*NH, 3´H), 4.32-4.19 (m, 1 H, 4´H), 3.89-3.76 (m, 1.30 H, POCH*H*, C*H*NH), 3.72 (s, 6 H, OCH_3), 3.69-3.50 (m, 3 H, POC*H*H, NC*H*(CH_3)$_2$), 3.42-3.37 (m, 1 H, 5´H), 3.29-3.20 (m, 1 H, 5´´H), 2.78 (t, *J* = 6.3 Hz, 1 H, CH*H*CN), 2.55 (t, *J* = 6.6 Hz, 1 H, C*H*HCN), 1.75 (bd, *J* = 10.8 Hz, 2 H, CH*H*CH), 1.68-1.52 (m, 2 H, C*H*HCH), 1.22-1.09 (m, 21 H, CH_3), 1.05-1.02 (m, 3 H, CH_3), 0.75-0.64 (m, 9 H, SiC(CH_3)$_3$), (-0.05) – (-0.10) (m, 3 H, SiCH_3), (-0.20) – (-0.28) (m, 3 H, SiCH_3) ppm (mixture of 2 rotamers at CN-bonds and 2 diastereomers). ^{13}C-NMR (75.5 MHz, d_6-DMSO): 158.1, 153.9, 152.7, 147.2, 144.8, 144.7, 139.8, 139.5, 135.4, 135.3, 135.2, 133.9, 133.8, 129.7, 128.9, 128.6, 127.7, 127.6, 126.7, 124.5, 118.8, 118.6, 113.1, 101.0, 87.5, 85.8, 85.7, 83.0, 82.8, 73.5, 72.3, 67.1, 63.2, 59.4, 58.9, 58.7, 57.7, 55.0, 44.4, 42.8, 42.7, 42.4, 42.3, 32.8, 29.0, 25.8, 25.4, 24.5, 24.3, 24.2, 24.1, 20.6, 20.0, 19.9, 19.8, 19.7, 17.6, -3.2, -5.0, -5.4 ppm. ^{31}P-NMR (121.5 MHz, d_6-DMSO): 149.5, 148.2 ppm. MS (ESI): *m/z* = 1204.37 [M + H^+]; calcd. for $C_{63}H_{87}N_9O_{11}PSi$ [M + H^+]: 1204.60.

Synthesis, purification and quantification of oligonucleotides

General. DNA and RNA synthesis was executed on an Expedite Nucleic Acid Synthesis System from PerSeptive Biosystems. Anion-exchange (AE) and Reversed Phase (RP) HPLC was performed on a Jasco LC-900 HPLC system mounted with a Jasco UV-975 detector (detection at 254 nm). For AE-HPLC a Dionex BioLC® DNAPac® PA-100 (250 × 9 mm) column and for RP-HPLC a preparative column Phenomenex Jupiter 4 µm Proteo 90 Å (250 × 10 mm) was used. All DNA and RNA samples were concentrated in a SpeedVac (Christ). Water was treated with DEPC and autoclaved.

Oligonucleotide synthesis. For DNA and RNA synthesis the standard synthesis protocols on a PerSeptive Expedite Synthezier at 1.0 µmol scale were used. Trichloroacetic acid in CH_2Cl_2 (deblock solution), acetic anhydride in THF (Cap A), *N*-methylimidazole in THF/pyridine (Cap B) and iodine in pyridine/H_2O/THF (oxidizer) were acquired from SAFC-Proligo (Sigma-Aldrich). Activator (0.35 M ETT in acetonitrile (molecular sieve)) was freshly prepared. Columns, which were purchased from Link Technologies, were self-packed with cpg-solid support. For DNA synthesis fast deprotecting amidites and for RNA synthesis fast deprotecting 2´-O-TBS amidites were used.

Isolation and purification. For deprotection and cleavage from solid support, cpg was removed from the column and treated for 20 h at 37 °C with 2 mL of a mixture of 32% aq ammonia/ethanol (3:1). Afterwards the supernatant was separated and the cpg material was washed two times with DEPC-H_2O. The combined fractions were evaporated to dryness. The crude DNA oligonucleotide was

purified by AE-HPLC. In case of RNA oligonucleotides, the residue was treated with 300 µL of a NMP/Et_3N/Et_3N·3HF (97%) mixture (6:3:4) for 90 min at 65 °C. For precipitation of the oligonucleotide, 1.2 mL *n*-butanol was added and the suspension was stored at −40 °C for 72 h. Afterwards it was centrifuged at 10000 rpm at 4 °C for 90 min. The supernatant was discarded and the crude RNA was purified by AE-HPLC. AE-HPLC conditions: (A: water, B: 1 M LiCl; gradient: 0–56% B within 32.00 min; flow: 5 mL/min) for 12mer DNAs (**22a**, **24a**) and 12mer RNA (**26a**) oligonucleotides; (A: water, B: 1 M LiCl; gradient: 0–10% from 0.00–2.50 min, 10–70% from 2.50–32.00 min; flow: 5 mL/min) for 18mer DNAs (**23a**, **25a**) and 18mer RNA (**27a**). An additional purification and desalting was done by RP-HPLC. RP-HPLC conditions: (A: 1 M TEAA buffer pH 7.0, B: acetonitrile, C: DEPC-H_2O; gradient: constant 10% A, 5% B from 0.00–3.00 min, 5–40% from 3.00–25.00 min; flow 4 mL/min) for all DNA (**22a**–**25a**) and RNA (**26a**, **27a**) oligonucleotides. The column was heated to 55 °C in most cases (55 °C and 20 °C for **27c**).

Quantification. Oligonucleotide concentrations were determined via UV spectrometry on a nanodrop2000 (Thermo Scientific) using Lambert-Beer´s law. Extinction coefficients were calculated by a nearest neighbor model according to literature [5]. For modified bases identical increments were used as for their natural counterparts.

Following sequences were prepared:

DNA:	dC TEMPO amidite **5**:	**22a** 5´-GCT GAT ATX AGC-3´
		**23a** 5´-GCT GAT GCA TGC ATX AGC-3´
	dA TEMPO amidite **7**:	**24a** 5´-GCT GAT ATC XGC-3´
		**25a** 5´-GCT GAT GCA TGC ATC XGC-3´
RNA:	A TEMPO amidite **8**:	**26a** 5´-GCU GAU AUC XGC-3´
		**27a** 5´-GCU GAU GCA UGC AUC XGC-3´

Good yields of all oligonucleotides were obtained after HPLC purification.

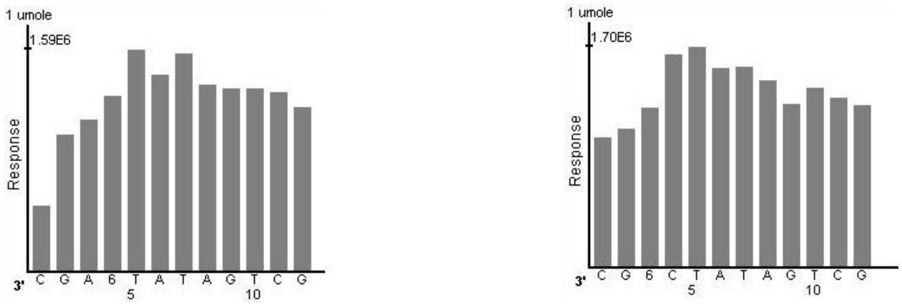

Figure S1: Trityl protocols of DNA synthesis: **22a** (left) and **24a** (right).

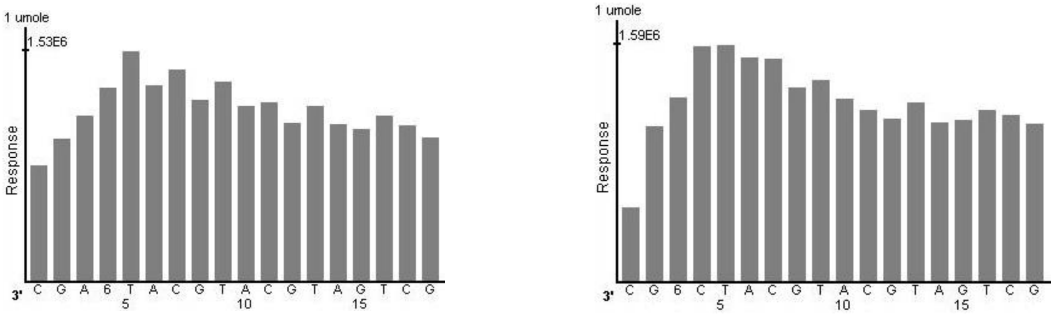

Figure S2: Trityl protocols of DNA synthesis: **23a** (left) and **25a** (right).

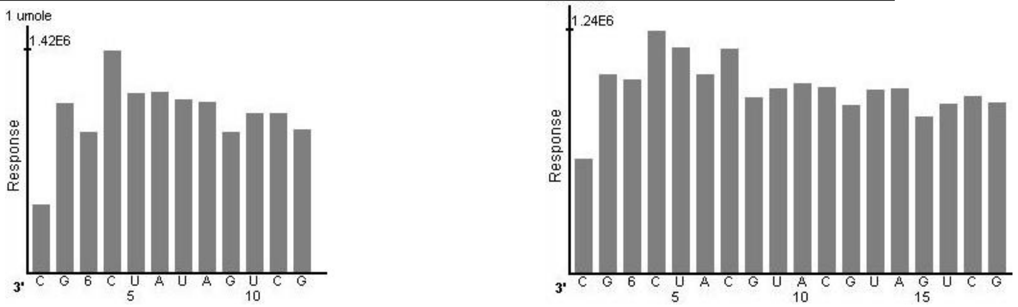

Figure S3: Trityl protocol of RNA synthesis **26a** (left) and **27a** (right).

Mass spectrometry. Oligonucleotides were analyzed via ESI mass spectrometry using a LC–MS instrument with microTOF-Q II analyser (Bruker). An Agilent 1200 Series HPLC using methanol/0.005 M TEAA buffer (gradient 0–60%) was applied as LC system.

DNA **22a** calculated exact mass: 3965.8; found 3965.6; 7930.6 (duplex)

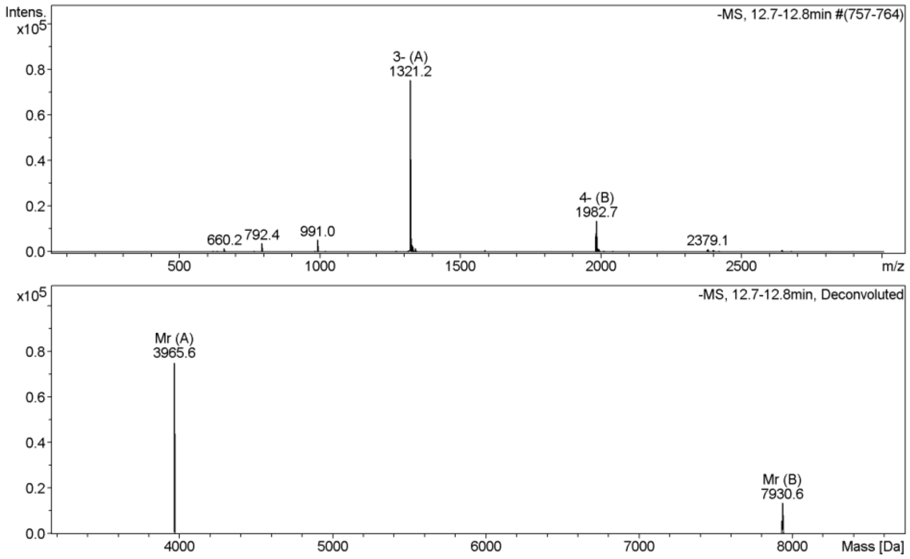

Figure S4: LC–MS analysis of protected DNA **22a**.

DNA **24a** calculated exact mass: 3965.8; found 3965.6; 7930.6 (duplex)

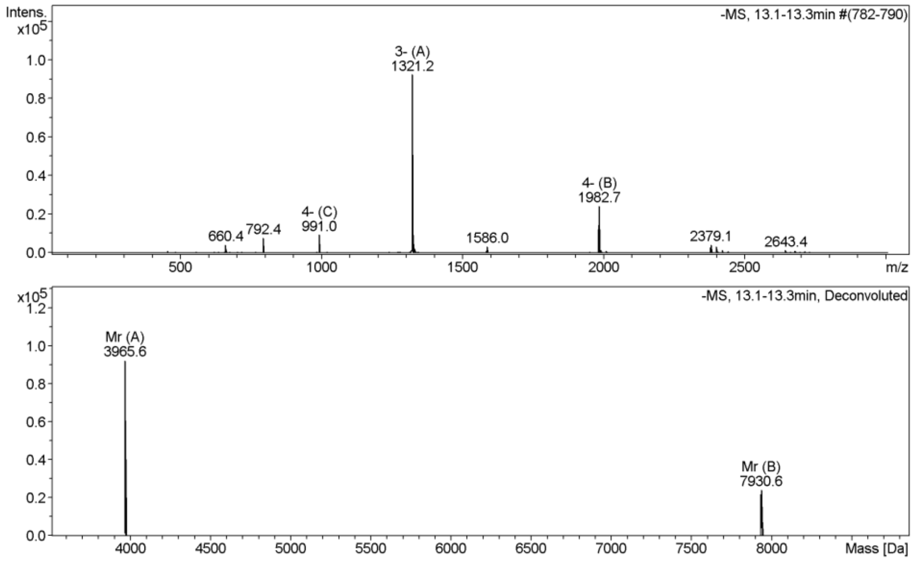

Figure S5: LC–MS analysis of protected DNA **24a**.

DNA **23a** calculated exact mass: 5819.9; found 5820.6

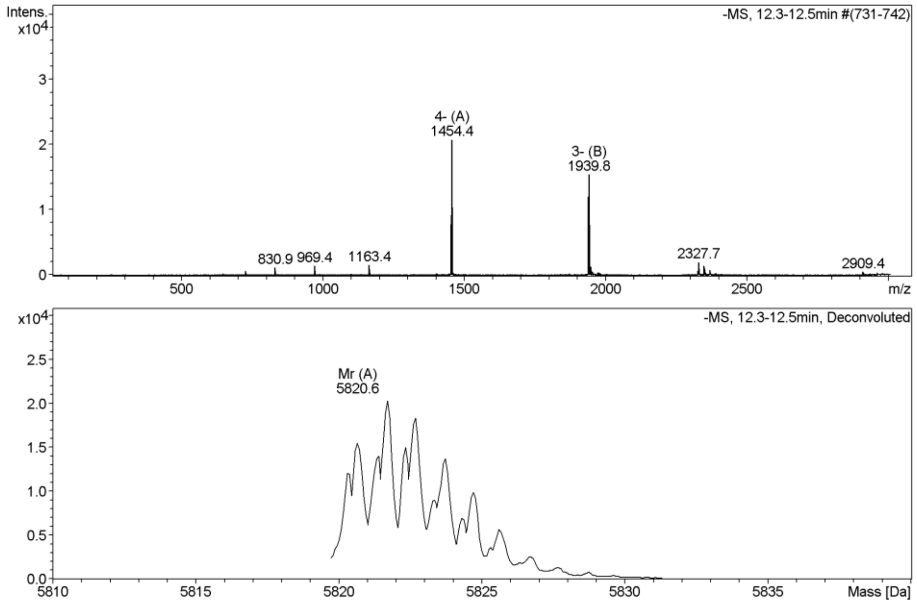

Figure S6: LC–MS analysis of protected DNA **23a**.

DNA **25a** calculated exact mass: 5819.9; found 5819.7

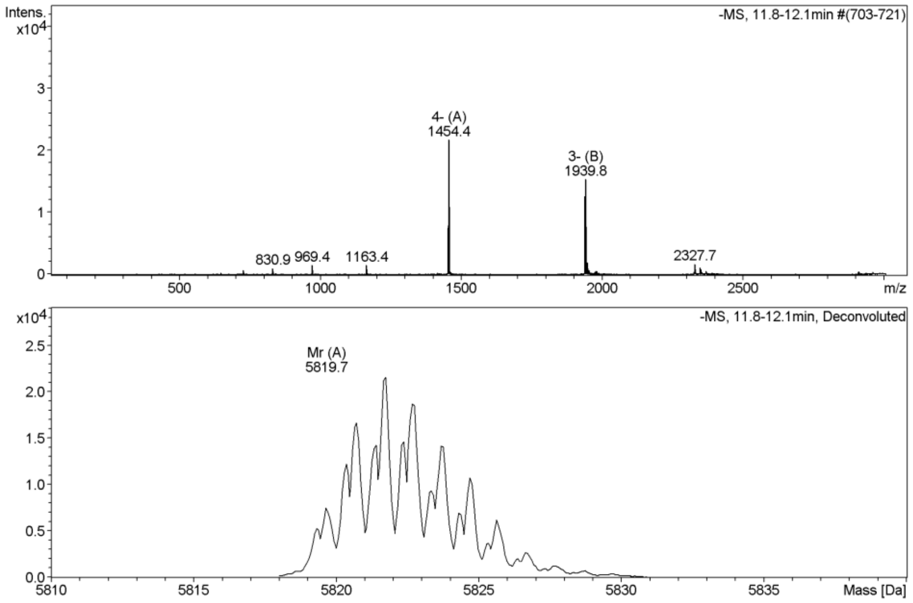

Figure S7: LC–MS analysis of protected DNA **25a**.

RNA **26a** calculated exact mass: 4115.7; found 4115.6, 8230.5 (duplex)

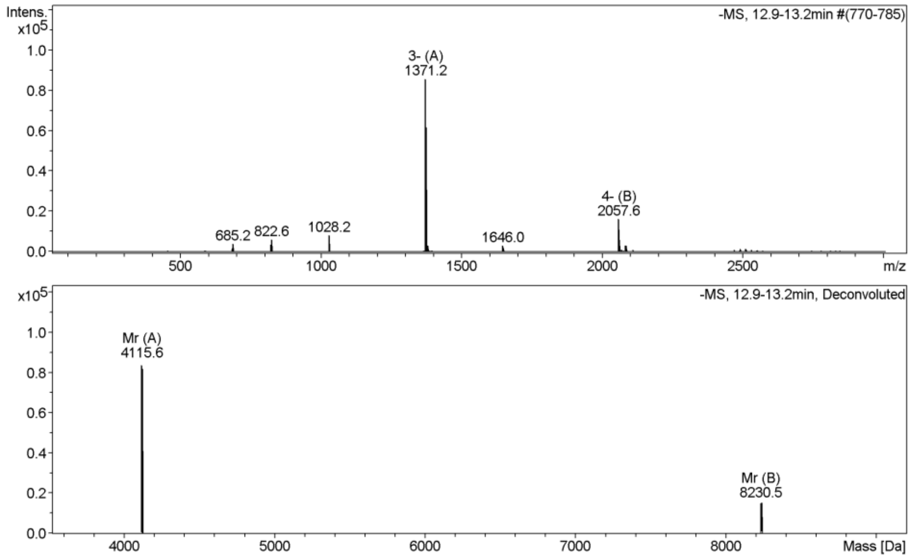

Figure S8: LC–MS analysis of protected RNA **26a**.

RNA **27a** calculated exact mass: 6051.6; found 6051.6, 6077.5 (+ Na^+)

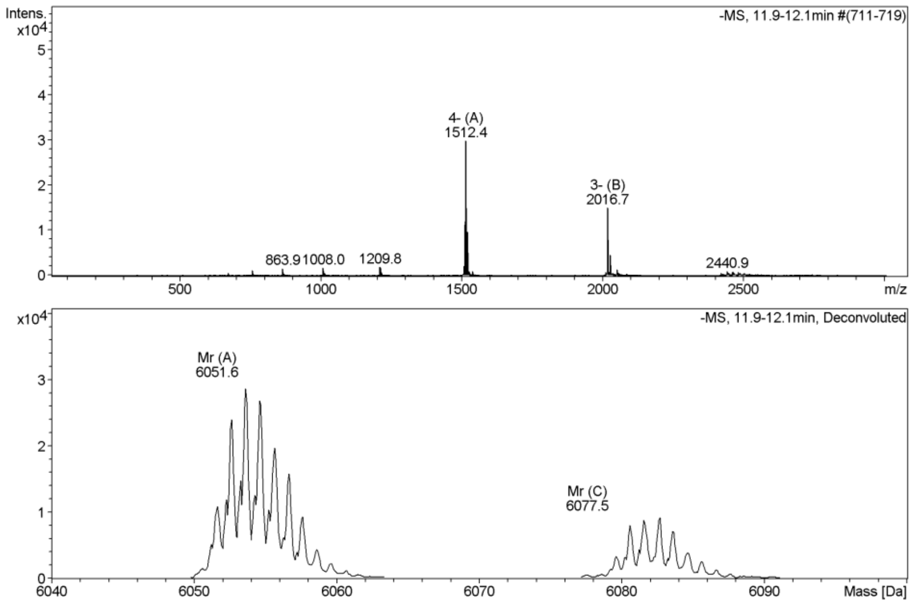

Figure S9: LC–MS analysis of protected RNA **27a**.

Photochemical deprotection of oligonucleotides

Conditions for deprotection. Sample (100 µL; 100 µM, 100 mM NaCl; 10 mM NaH_2PO_4/Na_2HPO_4 pH 7.4) was irradiated in a custom built apparatus containing LEDs (Nichia NCCU033, 365 nm, each with 100 mW optical output power) [6] for 20 min in a round glass cuvette (Carl Roth 50 × ø 10 mm) and subsequently annealed (see Table S1). Conditions for RP-HPLC: A: 1 M TEAA buffer pH 7.0, B: acetonitrile, C: DEPC-H_2O; gradient: constant 10% A, 5% B from 0.00–3.00 min, 5–20% from 3.00–25.00 min; flow 4 mL/min; column temperature 55 °C. A semipreparative Phenomenex Jupiter 4 µm Proteo 90 Å column (250 × 10.0 mm) was used.

RNA **26b** calculated mass: 3980.6; found 3950.4, 7900.2 (duplex) (Hemiacetal decomposes during measurement). RNA **26c** calculated mass: 3949.5; found 3949.4

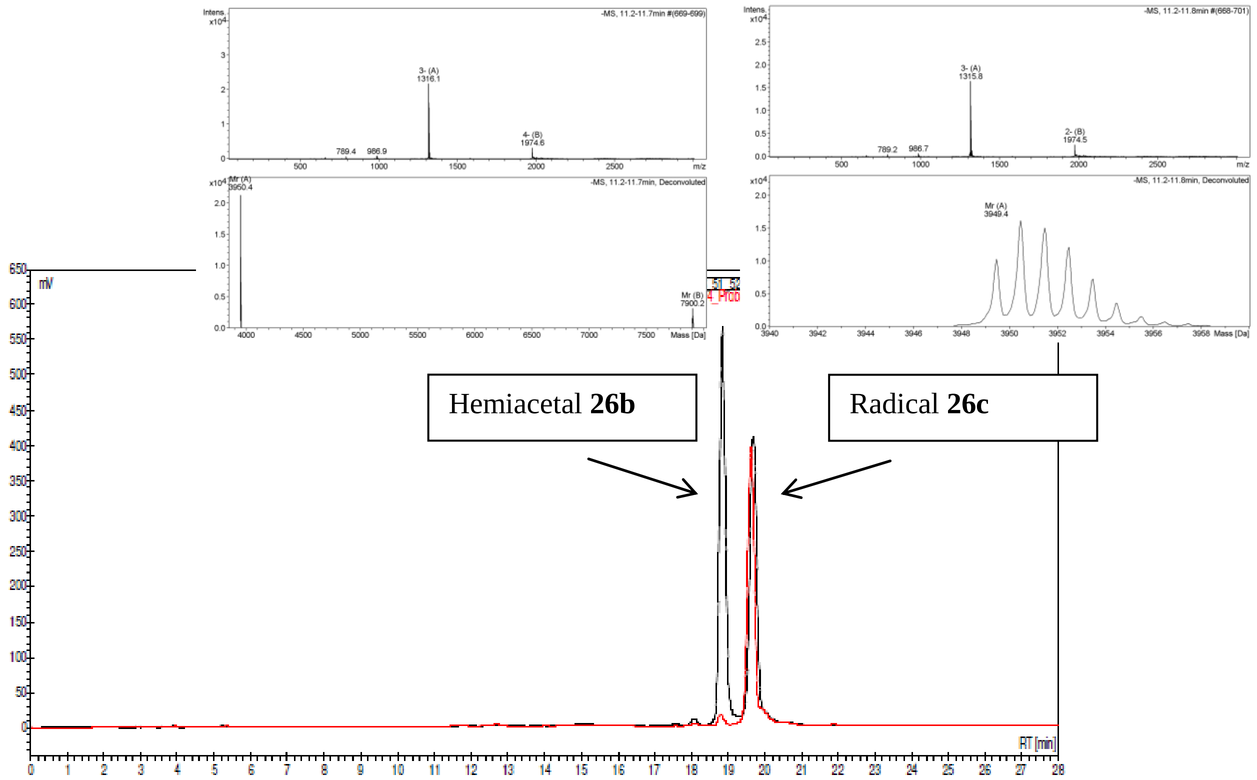

Figure S10: Deprotection of RNA **26a** with annealing (red) and without annealing after irradiation (black). Annealing procedure is shown in Table S1 below. This reaction is shown as an example. Insets: LC–MS analysis of hemiacetal **26b** and of spin-labeled RNA **26c**.

**t (min)**	**T (°C)**	**(°C/s)**
	20.0 → 90.0	3.0
70.00	90.0	
	90.0 → 20.0	0.1
1.00	20.00	

Table S1: Annealing procedure performed in a Biometra T-Personal Thermocycler.

DNA **22c** calculated mass: 3799.6; found 3799.5

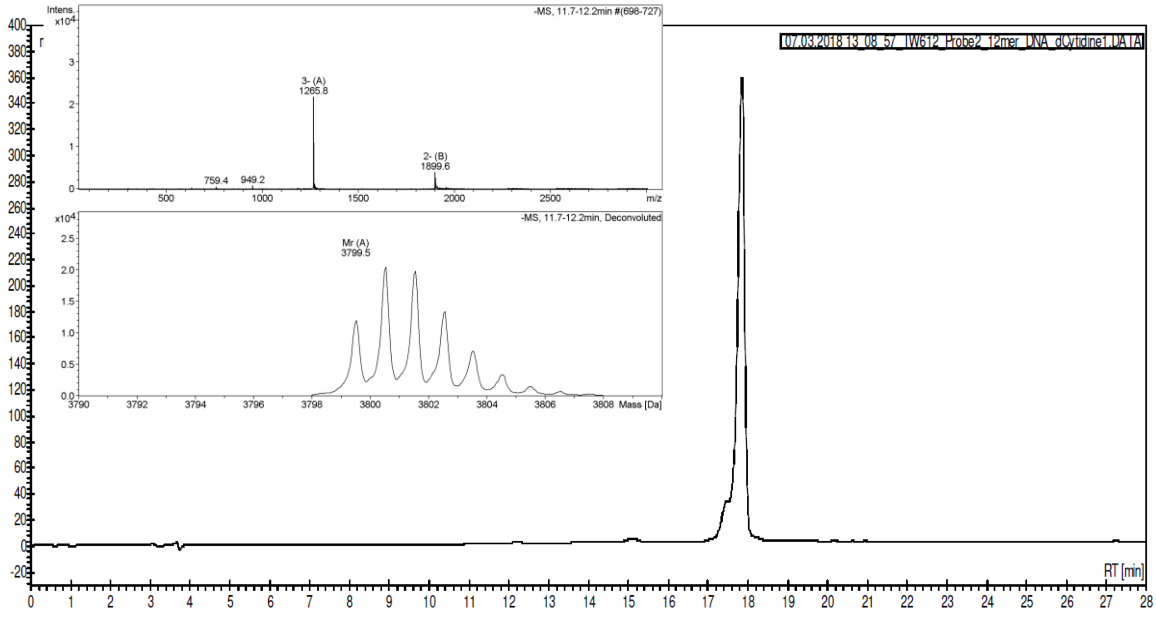

Figure S11: Deprotection of DNA **22a** after annealing procedure. Insets: LC–MS analysis of spin labeled DNA **22c**.

DNA **24c** calculated mass: 3799.6; found 3799.5

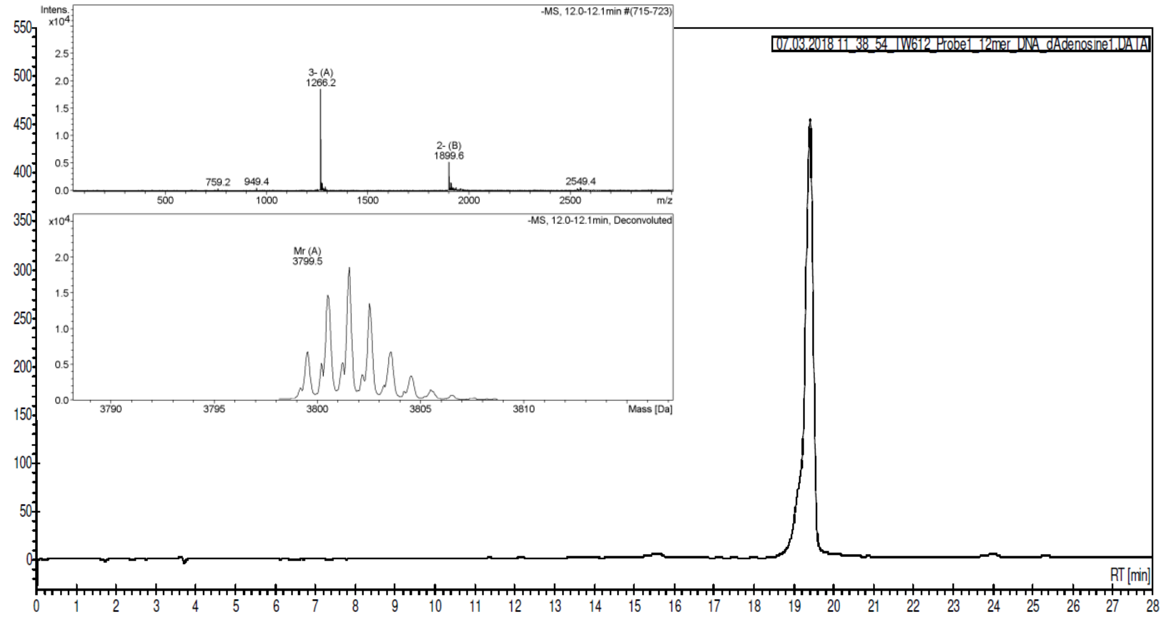

Figure S12: Deprotection of DNA **24a** after annealing procedure. Insets: LC–MS analysis of spin labeled DNA **24c**.

RNA **26c** calculated mass: 3949.5; found 3949.4

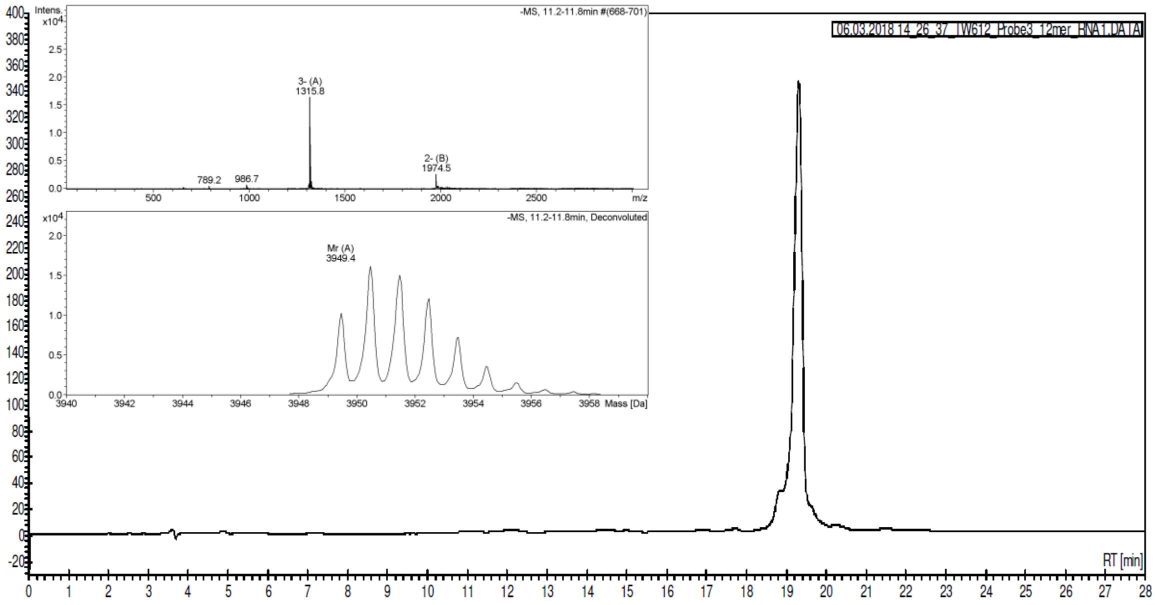

Figure S13: Deprotection of RNA **26a** after annealing procedure. Insets: LC–MS analysis of spin labeled RNA **26c**.

DNA **23c** calculated mass: 5653.8; found 5653.5

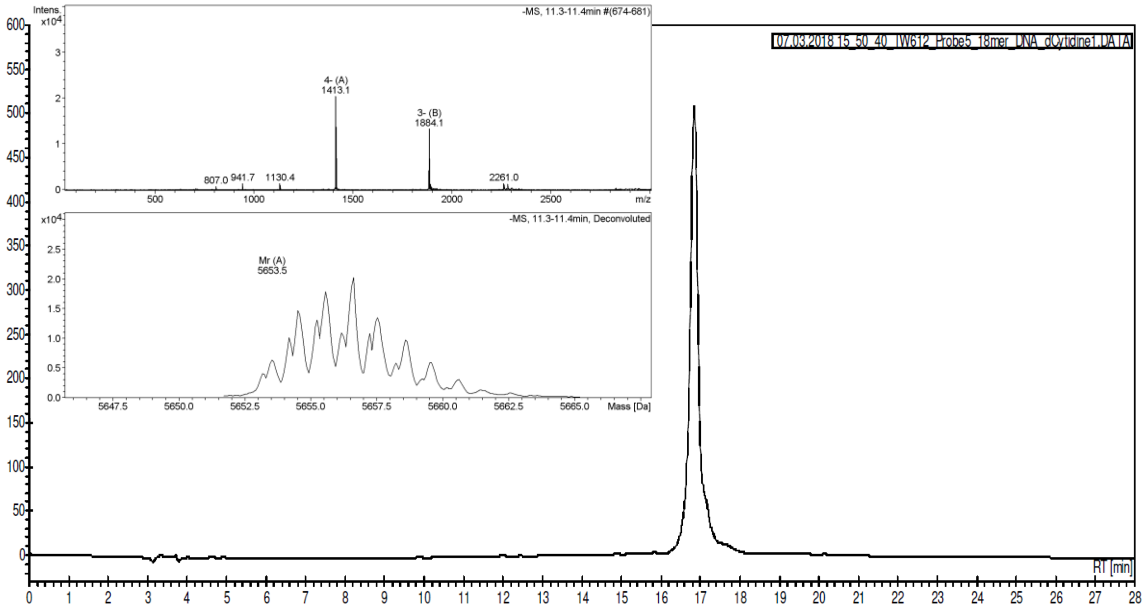

Figure S14: Deprotection of DNA **23a** after annealing procedure. Insets: LC–MS analysis of spin labeled DNA **23c**.

DNA **25c** calculated mass: 5653.8; found 5653.5

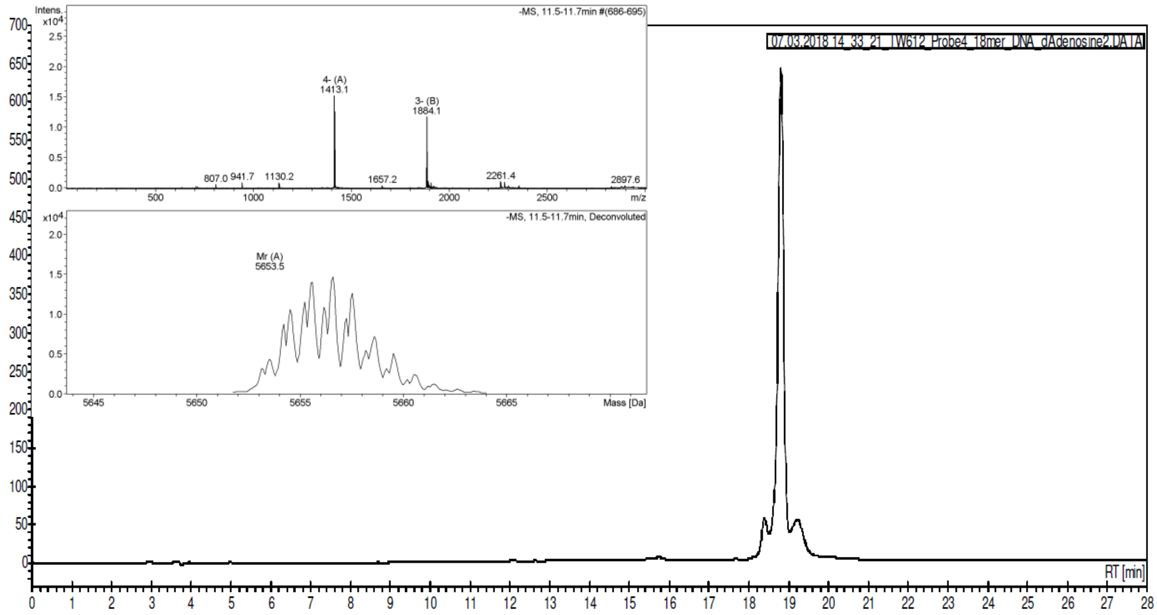

Figure S15: Deprotection of DNA **25a** after annealing procedure. Insets: LC–MS analysis of spin labeled DNA **25c**.

RNA **27c** calculated mass: 5885.7; found 5886.5, 5911.5 (+ Na^+)

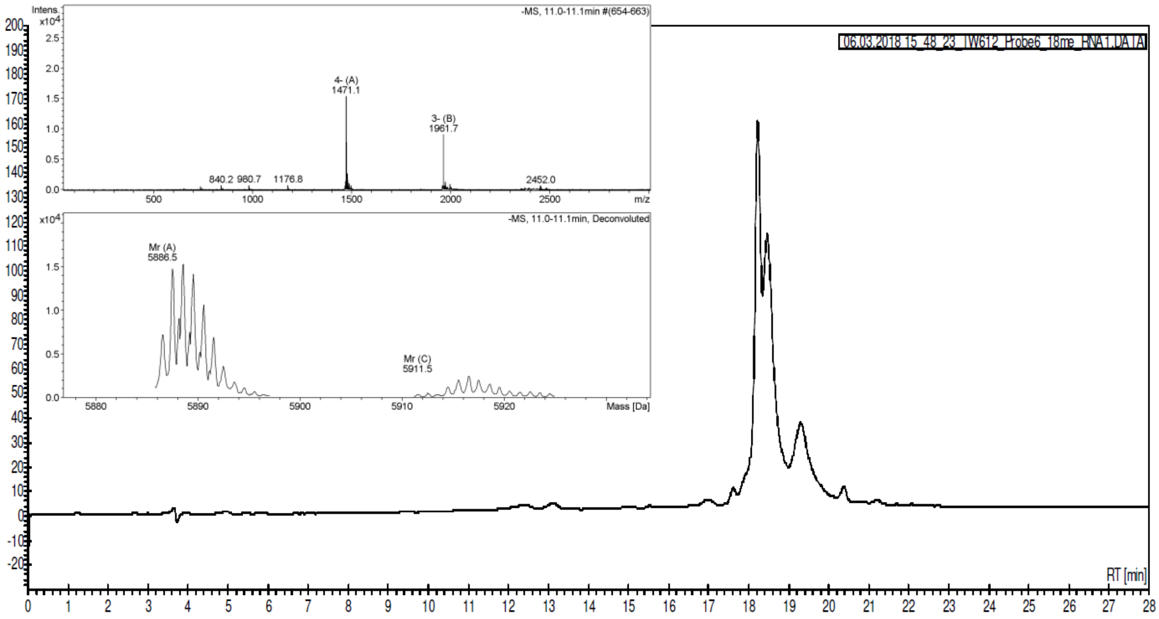

Figure S16: Deprotection of RNA **27a** after annealing procedure. Insets: LC–MS analysis of spin labeled RNA **27c**. The chromatogram shows three major peaks giving identical mass spectra. The sample also looks homogeneous in gel electrophoresis (Figure S19). This behaviour suggests the presence of different stable conformers under HPLC conditions. Strong support comes from HPLC separations at 20 °C: The main components are in equilibrium with each other (Figures S17, S18).

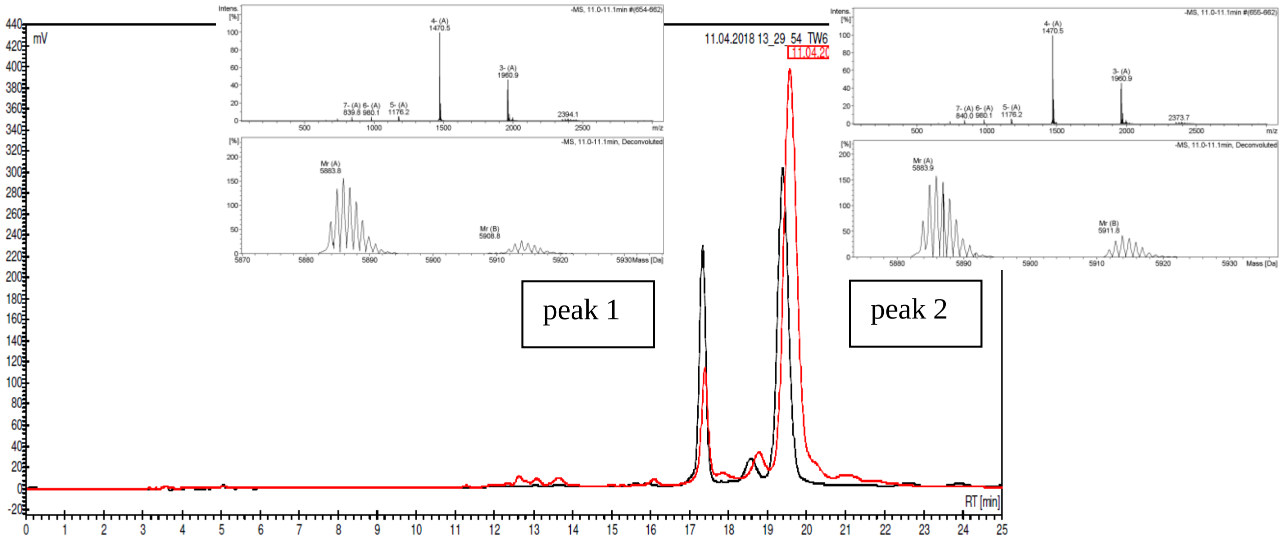

Figure S17: Deprotection of RNA **27a** with annealing (red) and without annealing (black). Column temperature 20 °C. Insets: LC–MS analysis of spin labeled RNA **27c**. Continued heating of the sample does not further change the ratio of peaks thus ruling out that peak 1 corresponds to the hemiacetal **27b**. After preparative separation, peak 1 and peak 2 form a mixture of both after standing or induced by a second annealing procedure (Figure S18).

Peak 1 and 2 were separated and solvent was removed under reduced pressure. Subsequently both samples were resolved (100 µL; 100 mM NaCl; 10 mM NaH_2PO_4/Na_2HPO_4 pH 7.4), annealed again and reinjected. HPLC conditions: see above. Column was cooled to 20 °C.

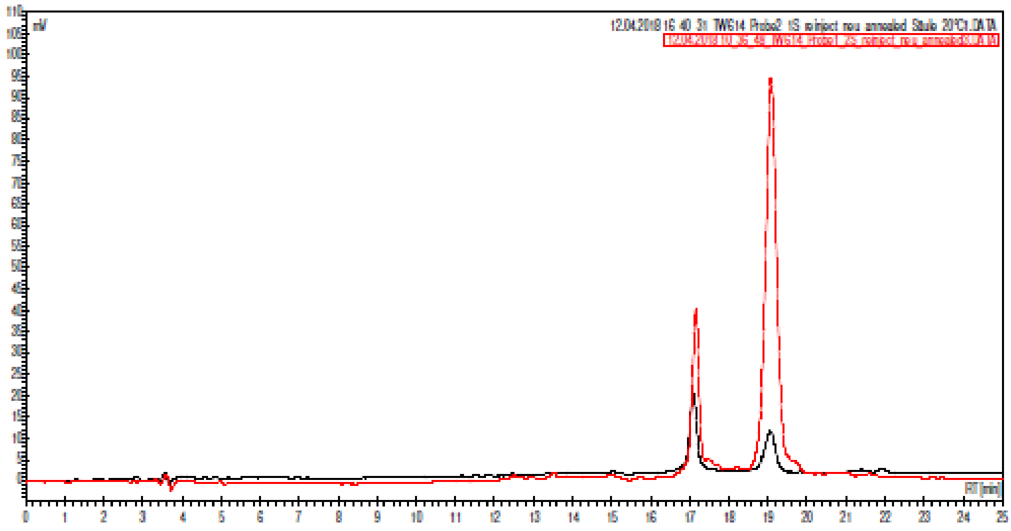

Figure S18: Overlay of peak 1 (black) and 2 (red) after clean separation and a second annealing step. The chromatograms show a conversion of peak 1 into peak 2 and vice versa suggesting a conformational equilibrium. If isolated peak 1 or 2 is kept at room temperature for several days, in both cases peak 1 dominates by far. A cautious interpretation is that the second peak might correspond to a stem-loop structure and peak 1 to the duplex, in accordance with PELDOR data (Figure S21).

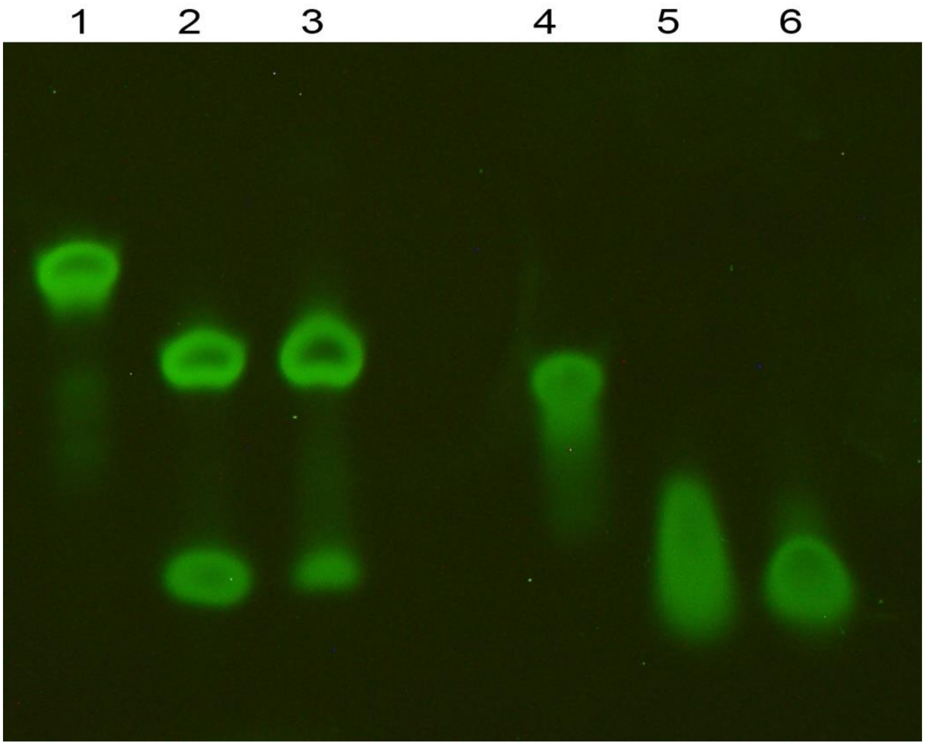

Figure S19: Analysis of deprotected palindromic oligonucleotides **22c**–**27c** by native 16% polyacrylamide gel electrophoresis conducted at 15 °C. Lane 1: RNA 18mer **27c** runs entirely in form of a duplex. Lane 2. DNA 18mer **23c** forms a palindromic duplex (top) and a monomeric hairpin structure (bottom). Lane 3. DNA 18mer **25c** again is a mixture of duplex (top) and hairpin (bottom). Lane 4. RNA 12mer **26c** forms mainly a duplex but the smear indicates beginning strand dissociation. Lane 5. DNA 12mer **22c** is in equilibrium between palindromic duplex (top) and single strands or monomeric hairpins (bottom). Lane 6. DNA 12mer **24c** forms mainly single strands or monomeric hairpins.
The presence of monomeric species containing single spin labels is also visible in lower levels of modulation depth (Figure S21) for all DNA samples, in particular for **22c** and **24c**.

EPR method part

cw-EPR before and after annealing. Continuous wave (cw) EPR spectra were measured at X-band (9.4 GHz) and room temperature on a Bruker EMXnano benchtop spectrometer after irradiation and after additional annealing. The experimental parameters were: 1 mW microwave power, 1.5 G modulation amplitude, 100 kHz modulation frequency, 20.48 ms time constant and 80.74 ms conversion time. The cw-EPR spectra of the DNA and RNA oligonucleotides **22c**–**27c** after irradiation and after additional annealing are shown in Figure S20. Sole irradiation leads to a mixture of EPR-inactive hemiacetals **22b**–**27b** and EPR-active nitroxides **22c**–**27c**. The spin concentration is

increased after the following step of annealing, which leads to mean spin labeling efficiencies around 96%.

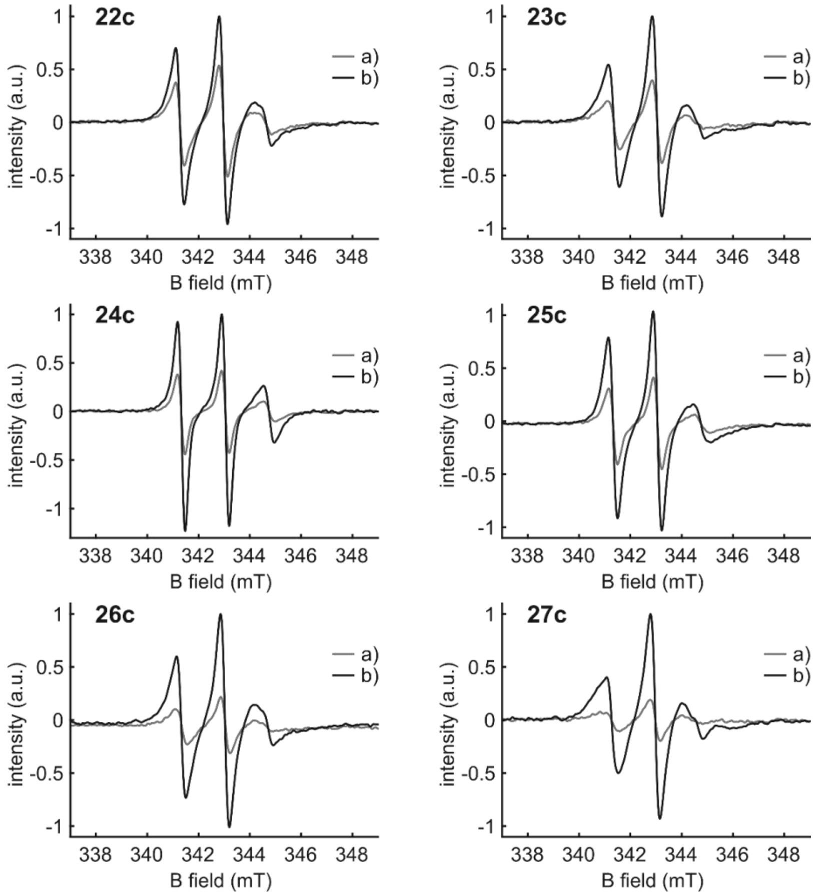

Figure S20: cw-EPR spectra of **22c-27c** a) directly after irradiation (grey) and b) after additional annealing (black).

PELDOR distance measurements. 10 μL of the samples mixed with 20% (v/v) deuterated glycerol as a cryoprotectant were transferred into 1.6 mm outer diameter EPR tubes (Suprasil, Wilmad LabGlass) and frozen in liquid nitrogen. PELDOR experiments were conducted at Q-band (33.8 GHz) and 50 K on a Bruker ELEXSYS E580 spectrometer equipped with a continuous-flow helium cryostat (CF935, Oxford Instruments), a temperature control system (ITC502, Oxford Instruments) and a 150 W TWT (Applied Systems Engineering Inc.) amplifier with a Bruker EN5107D2 cavity resonator. For all experiments the dead-time free four pulse PELDOR sequence [7] was applied with pulse lengths of 22 ns for the detection pulses (π/2 and π) and 12 ns for the pump pulse (π). The pump pulse frequency was set to the maximum of the echo detected field sweep spectrum and the frequency of the detection pulses 70 MHz lower. The first interpulse delay was increased by 16 ns for eight steps

to avoid deuterium modulation. Figure S21 shows the results of the PELDOR measurements for the samples **22c–27c** and Table S2 compares the experimentally obtained distances with the simulations.

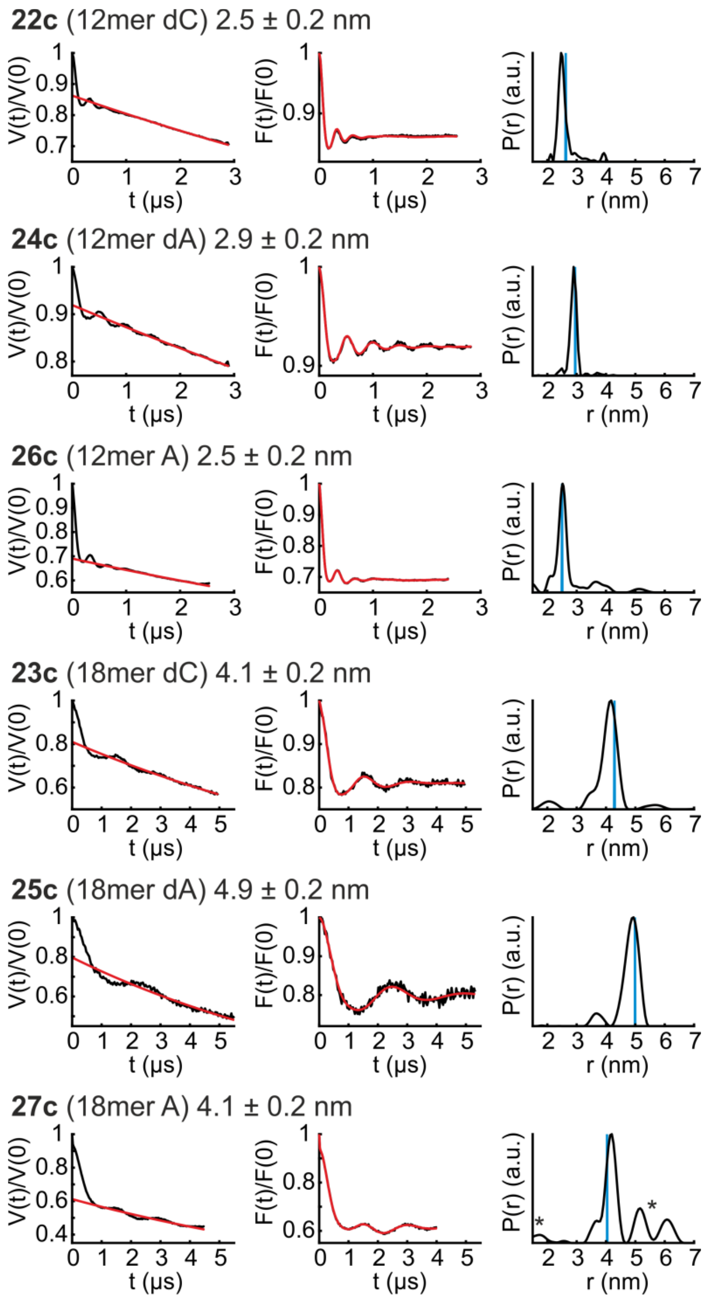

Figure S21: PELDOR measurements of **22c**–**27c**. After correction of the intermolecular exponential background (red) from the time traces V(t)/V(0) the form factors F(t)/F(0) were obtained. They were fitted with a model-free Tikhonov regularization (DeerAnalysis15) [8]. On the form factors the fits are superimposed (red). The distance distributions P(r) show distinct values and the asterisks indicate additional distances for the RNA sample **27c**, probably due to stacking of the RNA. DNA samples show reduced levels of modulation depth caused by the presence of monomeric strands (Figure S19). For example, the modulation depths λ of the palindromic 12mers can be compared with λ of an ideal 2-spin model system (λ = 0.31) to estimate the amount of the duplex structure. Taking the spin labeling efficiencies into account, the modulation depths suggest 100% duplex structure for sample **26c** (λ = 0.31) and roughly 45% and 25% duplex structure for samples **22c** (λ = 0.13) and **24c** (λ = 0.08), respectively. The distances predicted by molecular modeling are shown in blue.

Spin-spin-distances in palindromic duplexes

Sample	distance [Å]	PELDOR [nm]	Sample	distance [Å]	PELDOR [nm]
**22c**	25.4 (N-N)		**23c**	42.3 (N-N))	
	26.3 (N-O)			43.0 (N-O)	
	26.3 (O-N)			42.9 (O-N)	
	27.3 (O-O)			43.6 (O-O)	
**average**	26.3	2.5 ± 0.2	**average**	42.9	4.1 ± 0.2
**24c**	28.5 (N-N)		**25c**	49.0 (N-N)	
	29.4 (N-O)			50.0 (N-O)	
	29.7 (O-N)			49.8 (O-N)	
	30.6 (O-O)			50.8 (O-O)	
**average**	29.5	2.9 ± 0.2	**average**	49.9	4.9 ± 0.2
**26c**	24.0 (N-N)		**27c**	39.5 (N-N)	
	25.0 (N-O)			40.3 (N-O)	
	25.0 (O-N)			40.5 (O-N)	
	26.0 (O-O)			41.3 (O-O)	
**average**	25.0	2.5 ± 0.2	**average**	40.4	4.1 ± 0.2

Table S2: Comparison of the spin-spin distances in oligonucleotides **22c–27c** determined by PELDOR and by molecular modelling. The predicted distance is an average of N-N, N-O, O-N, and O-O distances.

Simulation of the spin-spin distances. All deoxyribonucleic acids (**22c-25c**) were generated as a B-form duplex using SPARTAN [9]. Ribonucleic acids (**26c, 27c**) were built as an A-form duplex. The attachment of the spin label was done with SPARTAN as well. After that, a local optimization, based on the force field MMFF94, was carried out applying AVOGADRO [10]. Optimization was executed twice for each duplex.

References

[1] Heckel, A.; Buff, M. C. R.; Raddatz, M.-S. L.; Müller, J.; Pötzsch, B.; Mayer, G. *Angew. Chem.* **2006**, 118, 6900-6902; *Angew. Chem. Int. Ed.* **2006**, *45*, 6748–6750.

[2] Weinrich, T.; Jaumann, E. A.; Scheffer, U.; Prisner, T. F.; Göbel, M. W. *Chem. Eur. J.* **2018**, *24*, 6202–6207.

[3] Gophane, D. P.; Sigurdsson, S. T. *Beilstein J. Org. Chem.* **2015**, *11*, 219–227.

[4] Bhattarai, S.; Freundlieb, M.; Pippel, J.; Meyer, A.; Abdelrahman, A.; Fiene, A.; Lee, S.-Y.; Zimmermann, H.; Yegutkin, G. G.; Sträter, N.; El-Tayeb, A.; Müller, C. E. *J. Med. Chem.* **2015**, *58*, 6248–6263 .

[5] Nanodrop: Gray, D. M.; Hung, S. H.; Johnson, K.H.; Absorption and Circular-Dichroism Spectroscopy of Nucleic-Acid Duplexes and Triplexes. *Methods Enzymol.* **1995**, *246*, 19–34.

[6] Seven, I.; Weinrich, T.; Gränz, M.; Grünewald, C.; Brüß, S.; Krstić, I.; Prisner, T. F.; Heckel, A.; Göbel, M. W. *Eur. J. Org. Chem.* **2014**, 4037–4043.

[7] Pannier, M.; Veit, S.; Godt, A.; Jeschke, G.; Spiess, H. W. *J. Magn. Reson.* **2000**, *142*, 331.

[8] Jeschke, G.; Chechik, V.; Ionita, P.; Godt, A.; Zimmermann, H.; Banham, J.; Timmel, C. R.; Hilger, D.; Jung, H. *Appl. Magn. Reson.* **2006**, *30*, 473.

[9] Spartan ´08 Version 1.2.0, Build 132, Jun 24 2009, Wavefunction Inc.

[10] Avogadro: an open-source molecular builder and visualization tool. Version 1.2.0. http://avogadro.cc/; Hanwell, M. D.; Curtis, D. E.; Lonie, D. C.; Vandermeersch, T.; Zurek, E.; Hutchison, G. R., *J. Cheminformatics* **2012**, *4*:17.

This report was created by ACD/NMR Processor Academic Edition. For more information go to www.acdlabs.com/nmrproc/

1-(3'-O-Acetyl-5'-O-DMT-2'-deoxyribofuranosyl)-4-(2,2,6,6-tetramethyl-1-((2-nitrobenzyl-oxy)methoxy)piperidin-4-ylamino)pyrimidin-2(1H)-one (11)

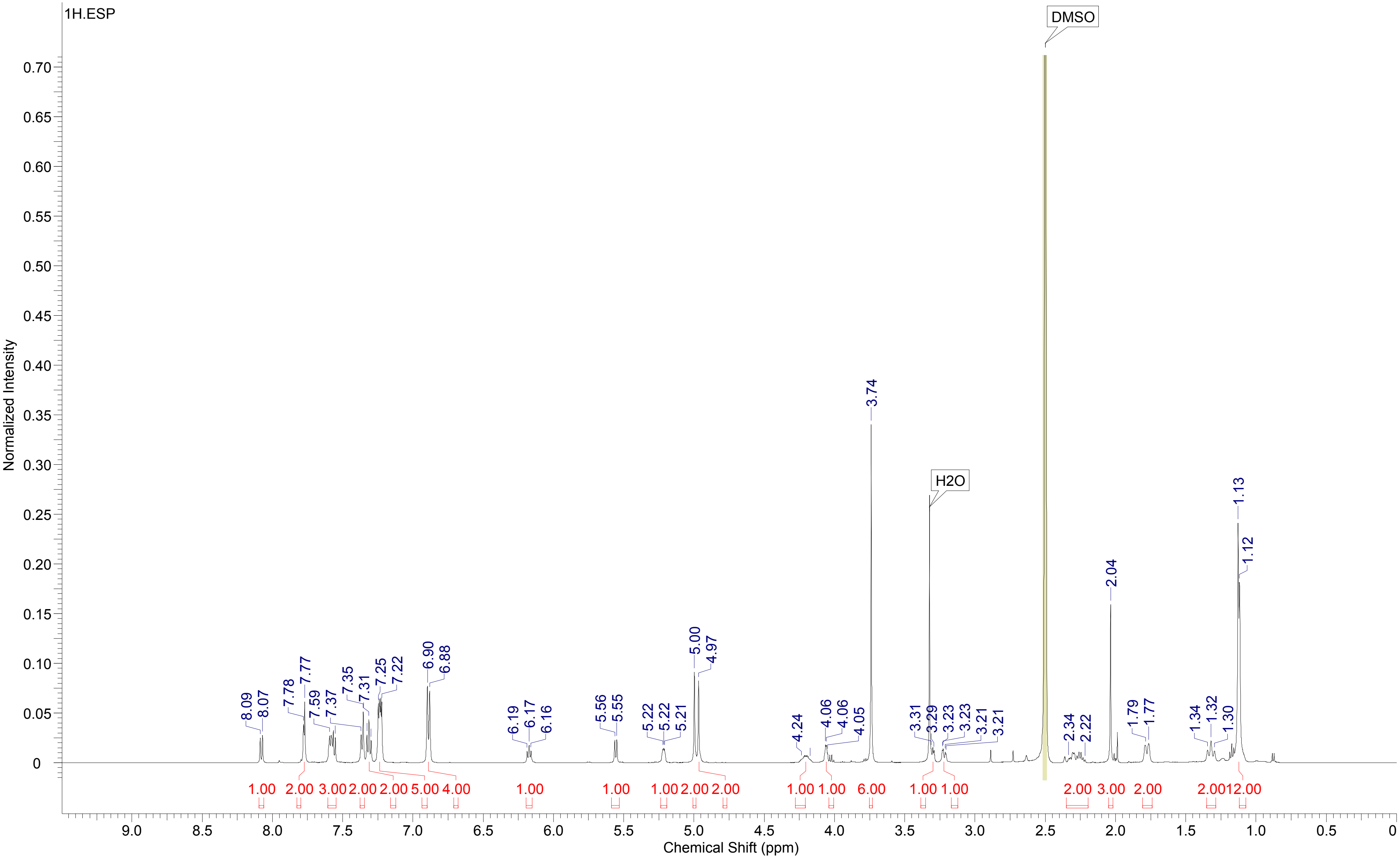

This report was created by ACD/NMR Processor Academic Edition. For more information go to www.acdlabs.com/nmrproc/

1-(3'-O-Acetyl-5'-O-DMT-2'-deoxyribofuranosyl)-4-(2,2,6,6-tetramethyl-1-((2-nitrobenzyl-oxy)methoxy) piperidin-4-ylamino)pyrimidin-2(1H)-one (11)

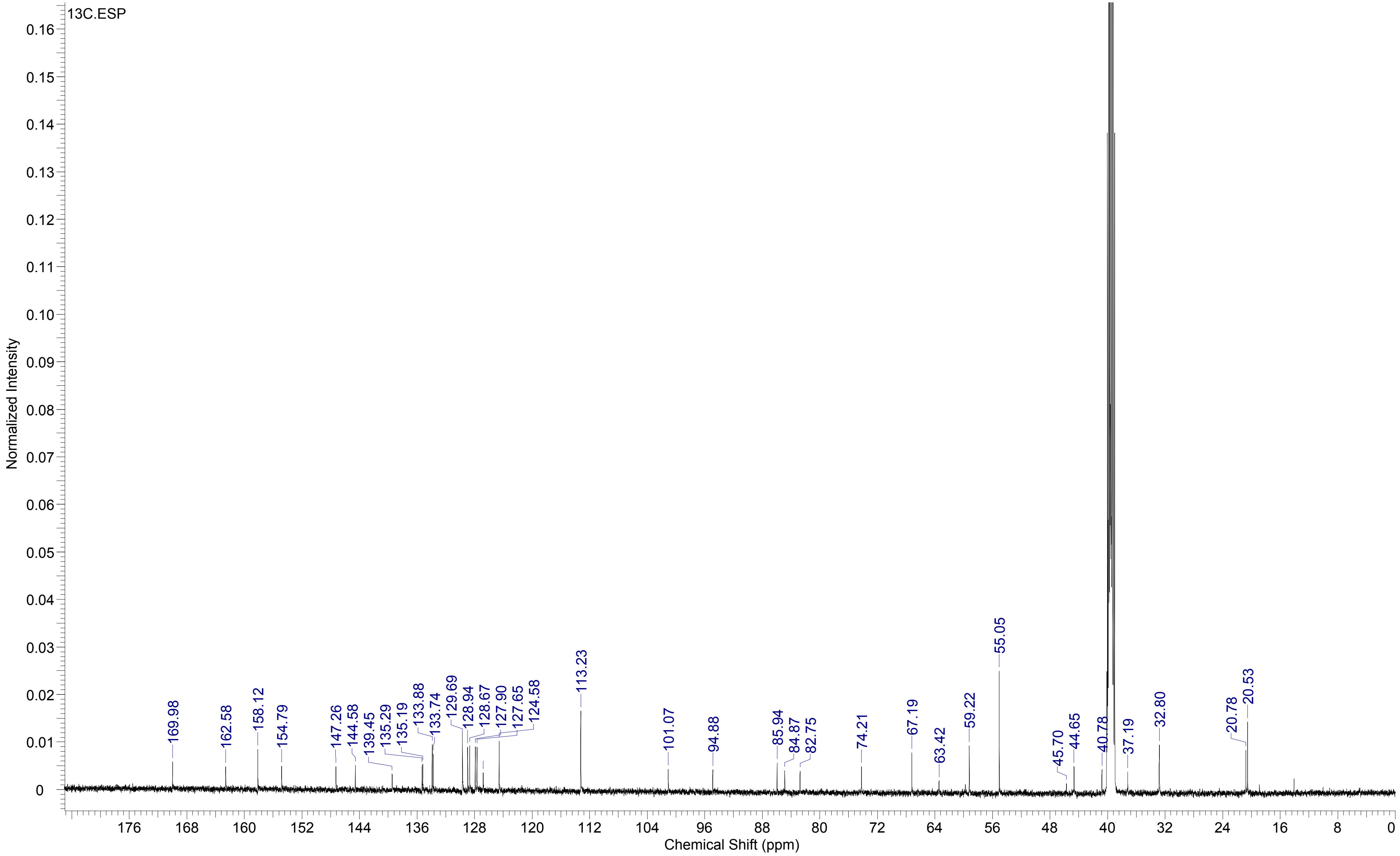

This report was created by ACD/NMR Processor Academic Edition. For more information go to www.acdlabs.com/nmrproc/

1-(5'-O-DMT-2'-Deoxyribofuranosyl)-4-(2,2,6,6-tetramethyl-1-((2-nitrobenzyloxy)methoxy)-piperidin-4-ylamino)pyrimidin-2(1H)-one (12)

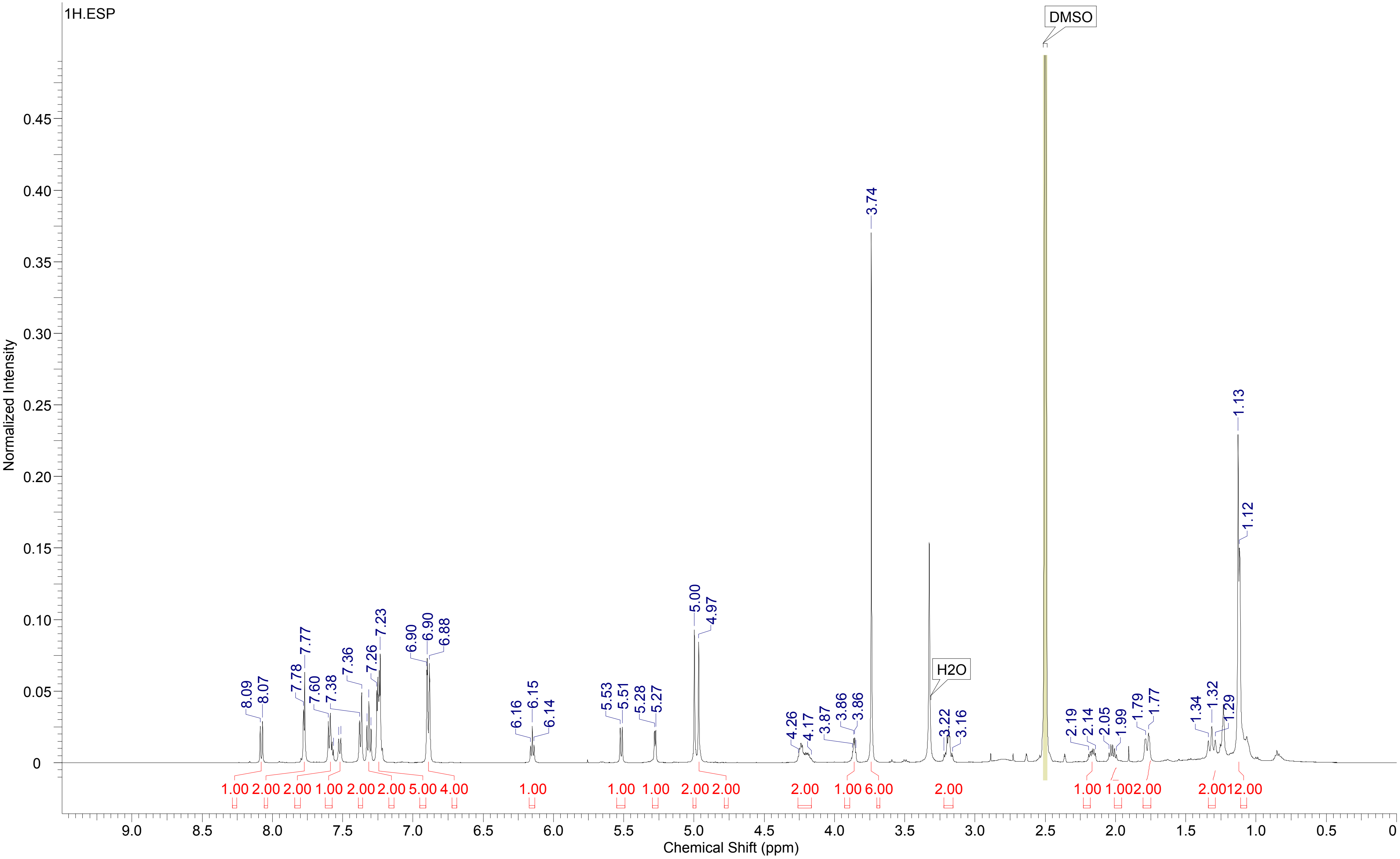

This report was created by ACD/NMR Processor Academic Edition. For more information go to www.acdlabs.com/nmrproc/

1-(5'-O-DMT-2'-Deoxyribofuranosyl)-4-(2,2,6,6-tetramethyl-1-((2-nitrobenzyloxy)methoxy)-piperidin-4-ylamino)pyrimidin-2(1H)-one (12)

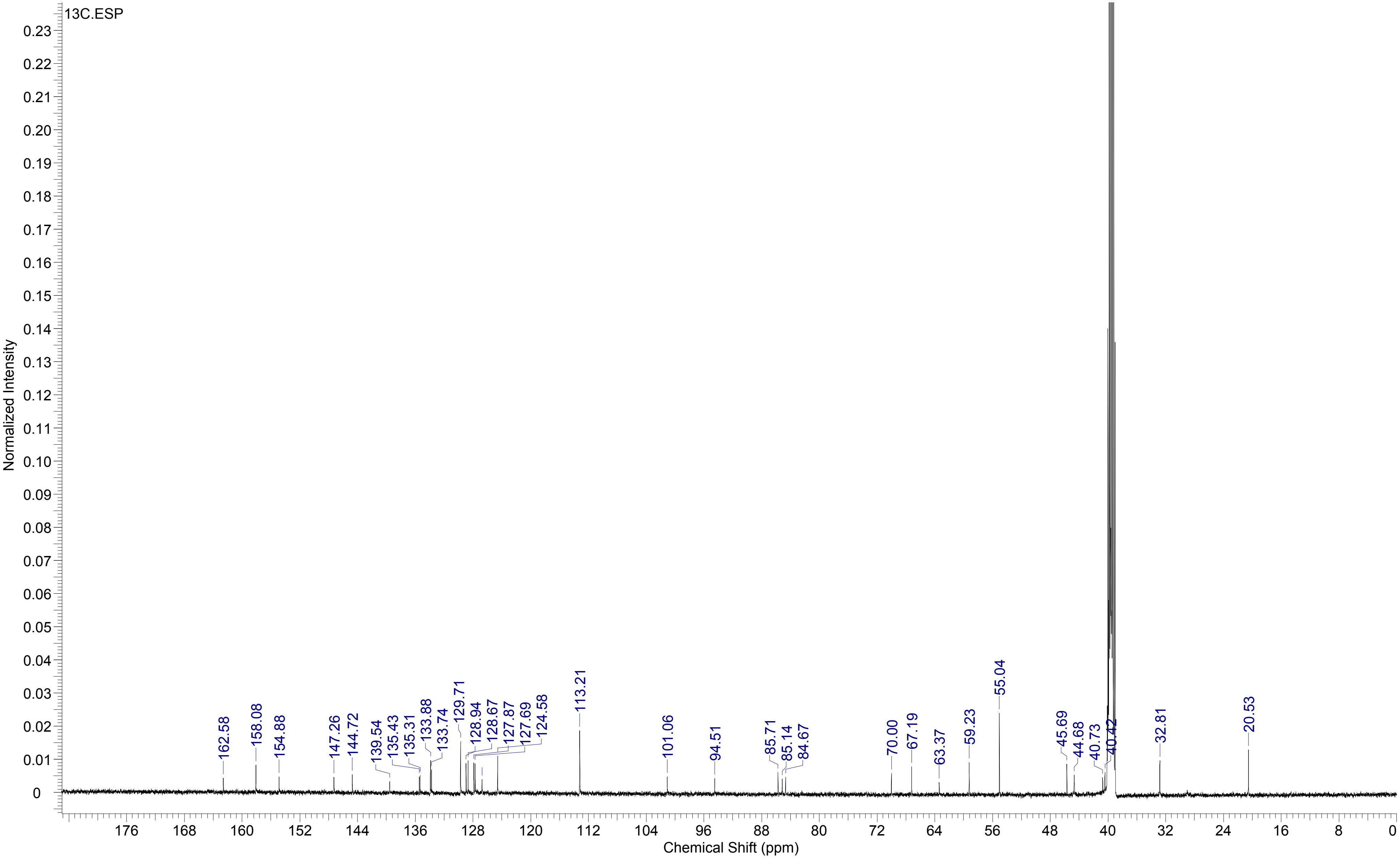

This report was created by ACD/NMR Processor Academic Edition. For more information go to www.acdlabs.com/nmrproc/

Deoxycytidine phosphoramidite with protected spin label (5)

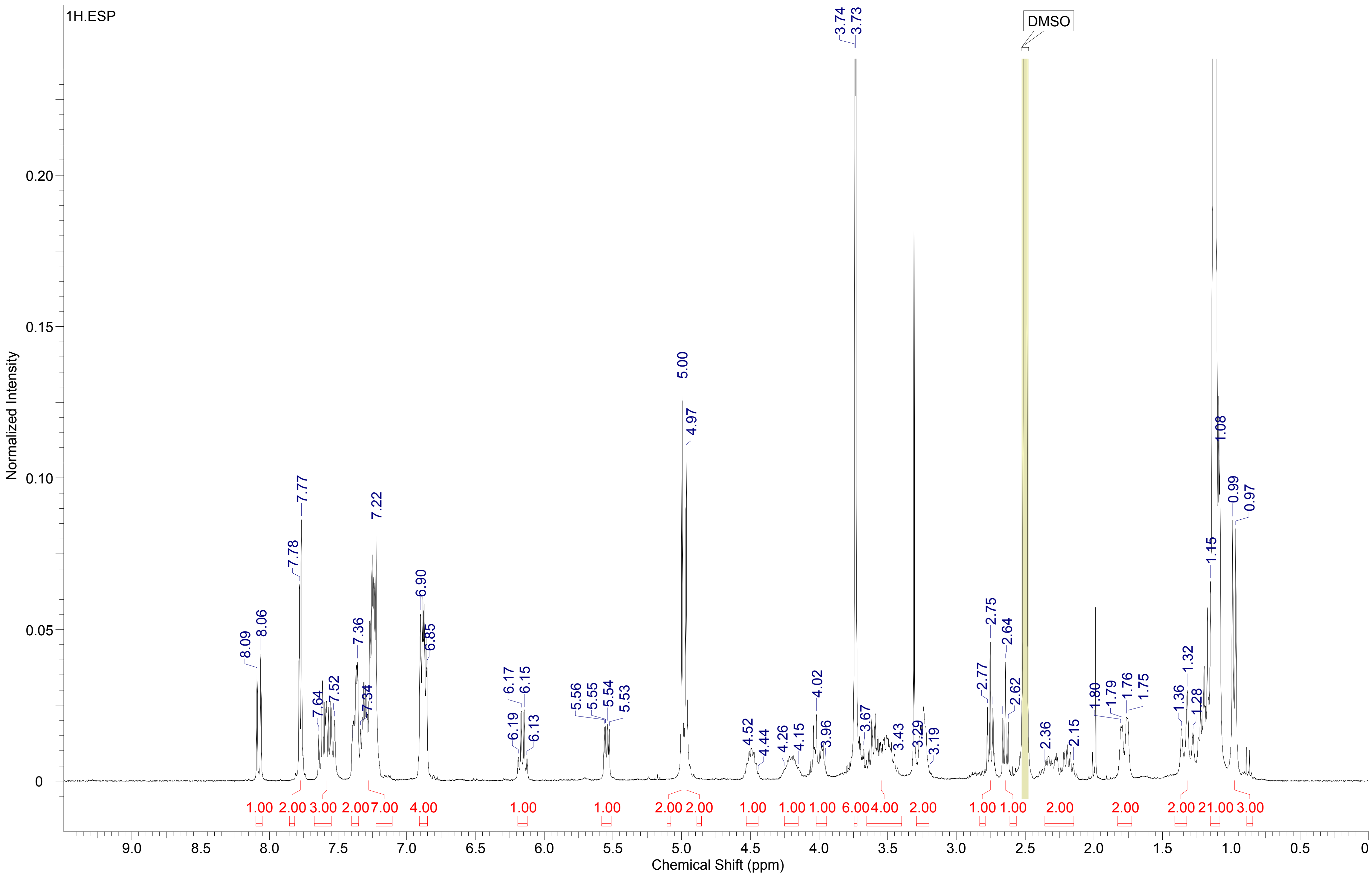

This report was created by ACD/NMR Processor Academic Edition. For more information go to www.acdlabs.com/nmrproc/

Deoxycytidine phosphoramidite with protected spin label (5)

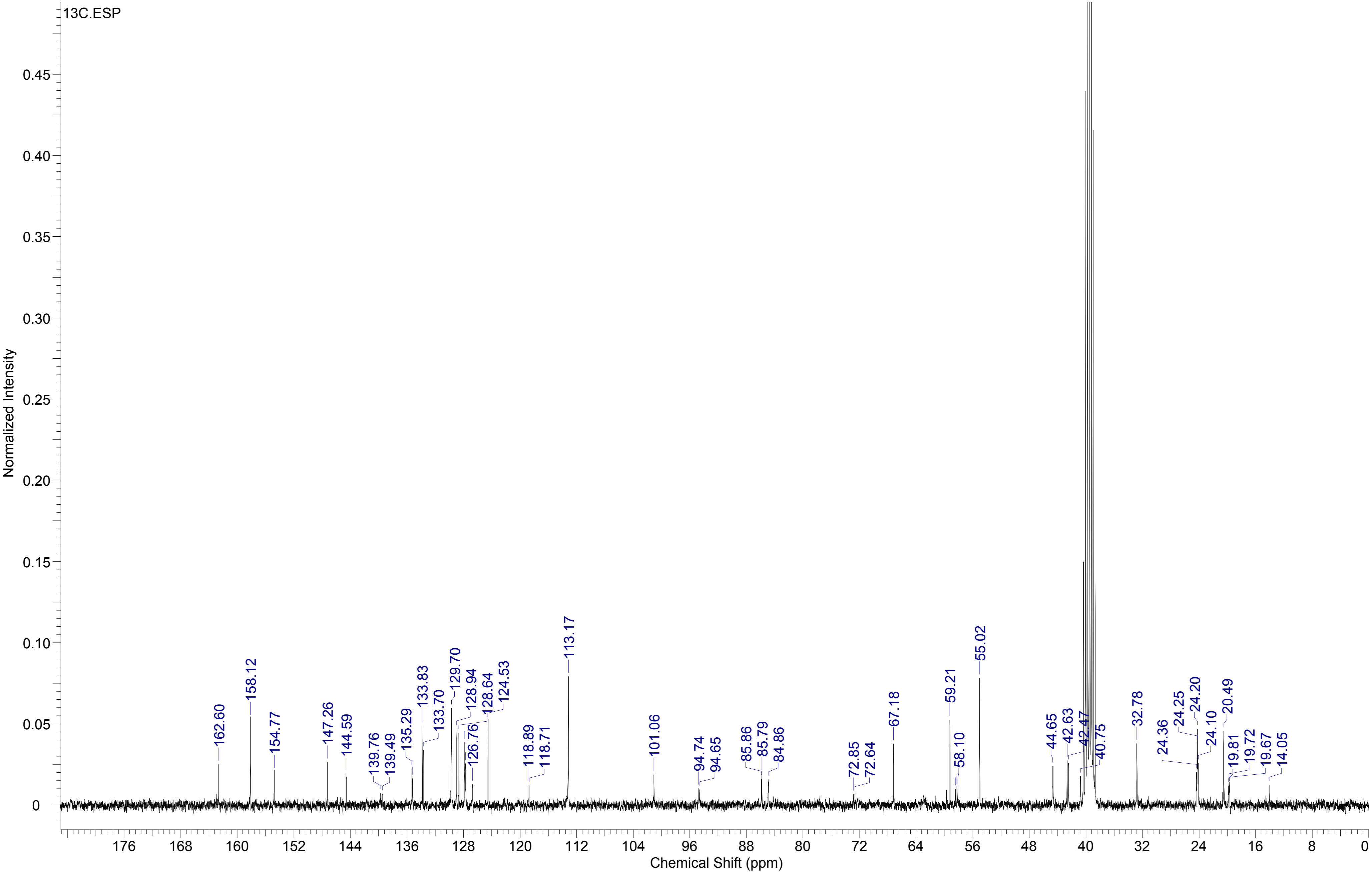

This report was created by ACD/NMR Processor Academic Edition. For more information go to www.acdlabs.com/nmrproc/

Deoxycytidine phosphoramidite with protected spin label (5)

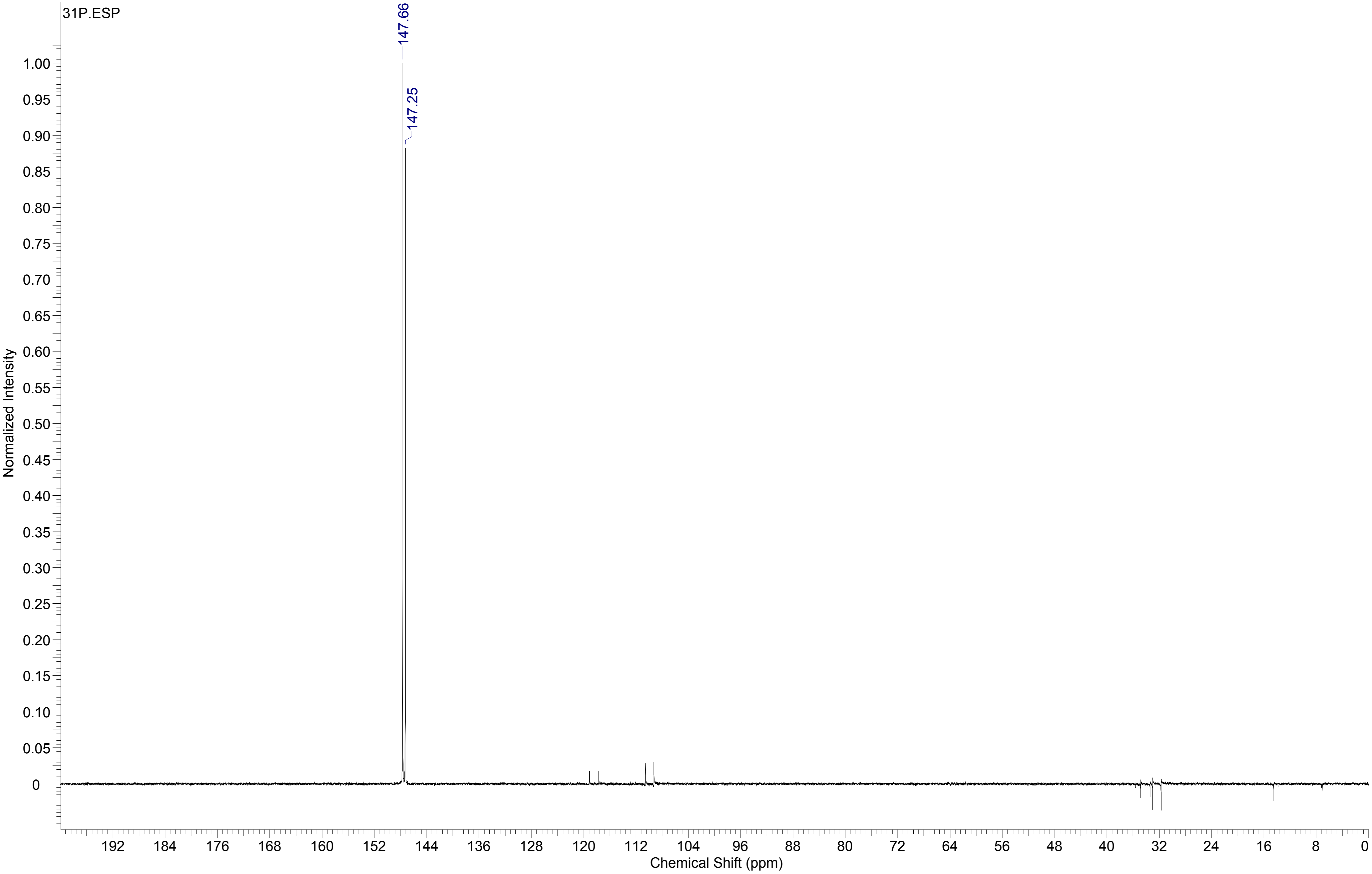

This report was created by ACD/NMR Processor Academic Edition. For more information go to www.acdlabs.com/nmrproc/

9-(3',5'-Tri-O-acetyl-2'-deoxyribofuranosyl)-6-chloro-purine (13)

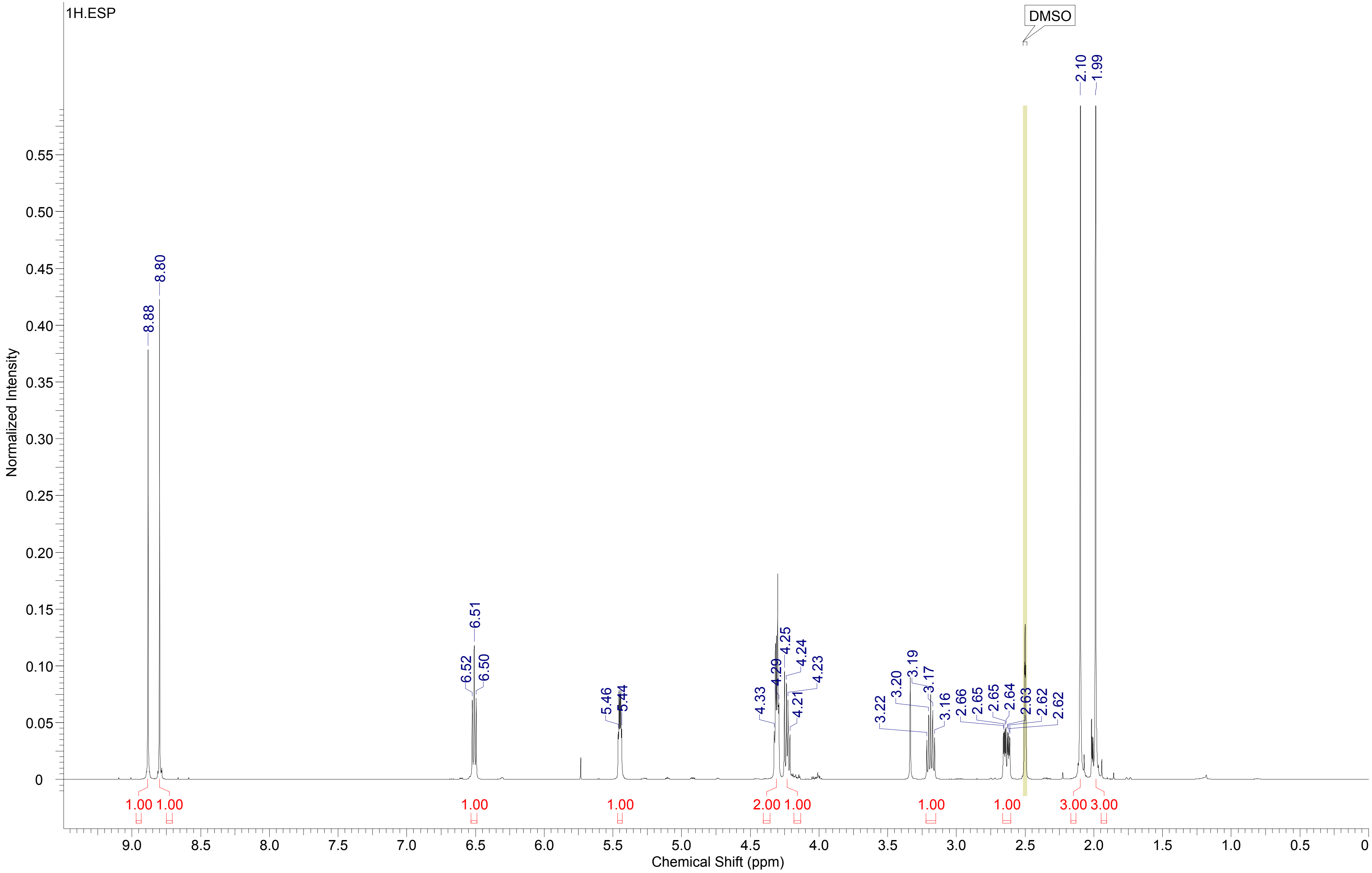

This report was created by ACD/NMR Processor Academic Edition. For more information go to www.acdlabs.com/nmrproc/

9-(3',5'-Tri-O-acetyl-2'-deoxyribofuranosyl)-6-chloro-purine (13)

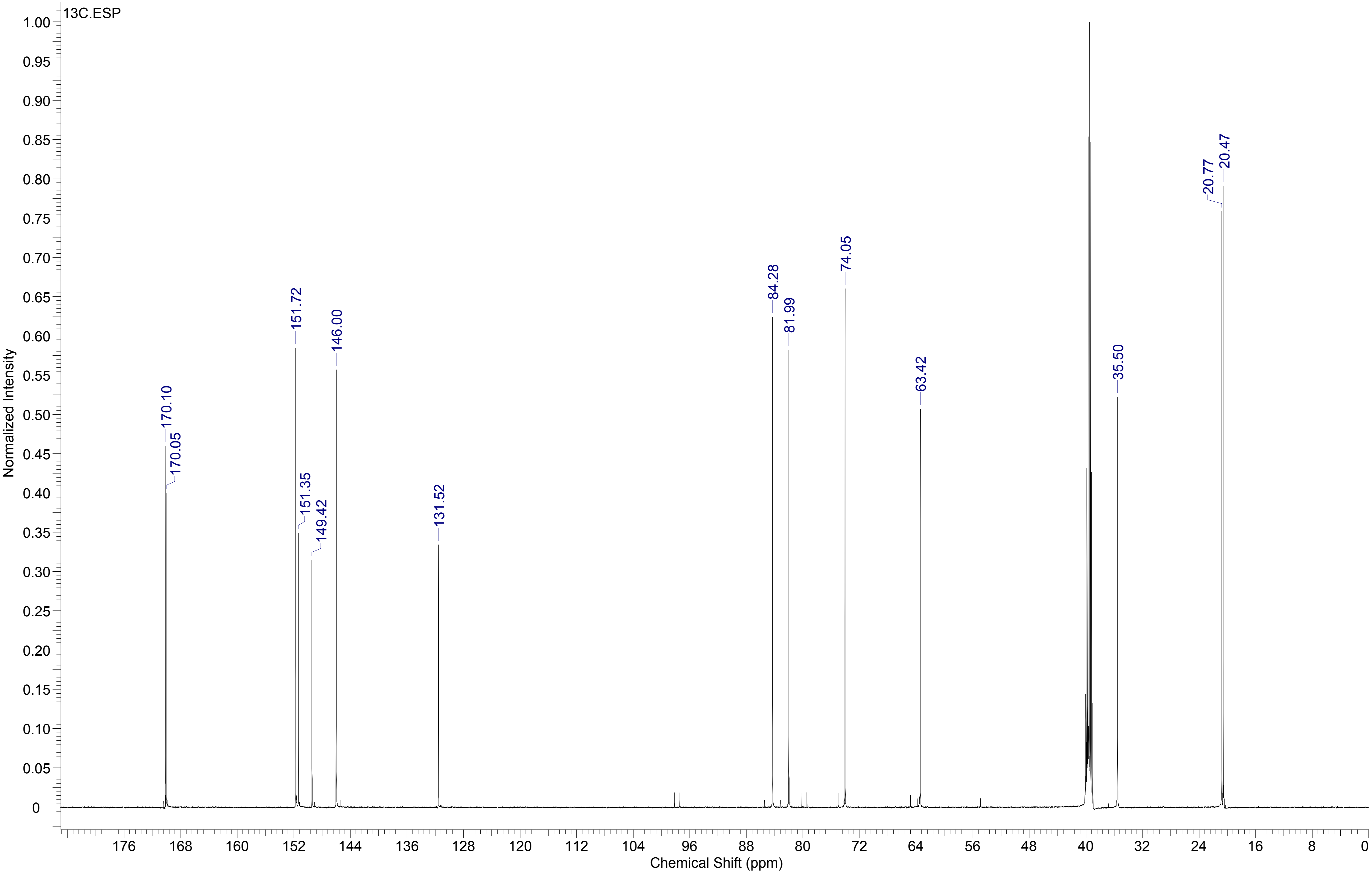

This report was created by ACD/NMR Processor Academic Edition. For more information go to www.acdlabs.com/nmrproc/

9-(3',5'-Di-O-acetyl-2'-deoxyribofuranosyl)-6-(2,2,6,6-tetramethyl-1-((2-nitrobenzyloxy)-methoxy) piperidin-4-ylamino)purine (14)

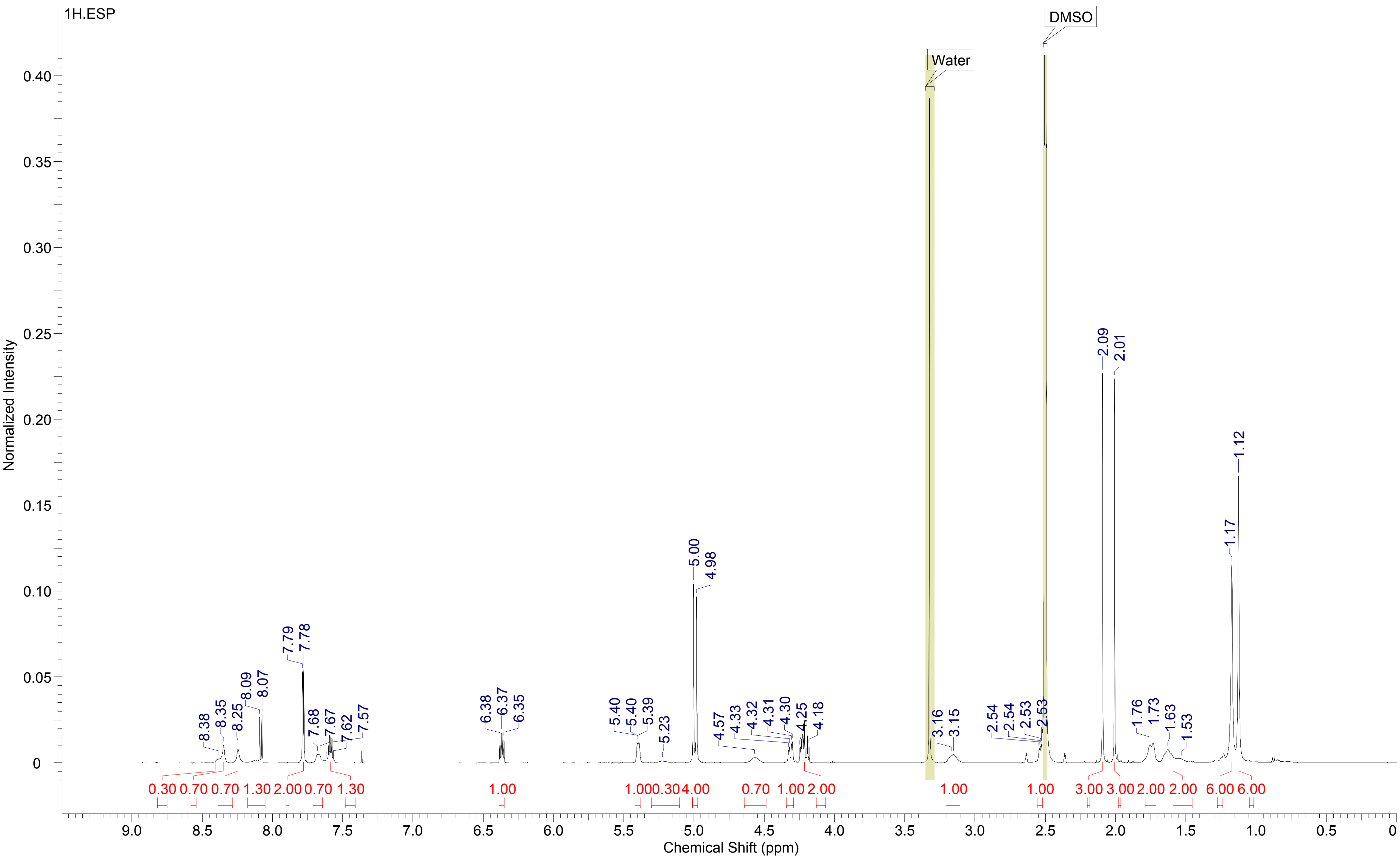

This report was created by ACD/NMR Processor Academic Edition. For more information go to www.acdlabs.com/nmrproc/

9-(3',5'-Di-O-acetyl-2'-deoxyribofuranosyl)-6-(2,2,6,6-tetramethyl-1-((2-nitrobenzyloxy)-methoxy) piperidin-4-ylamino)purine (14)

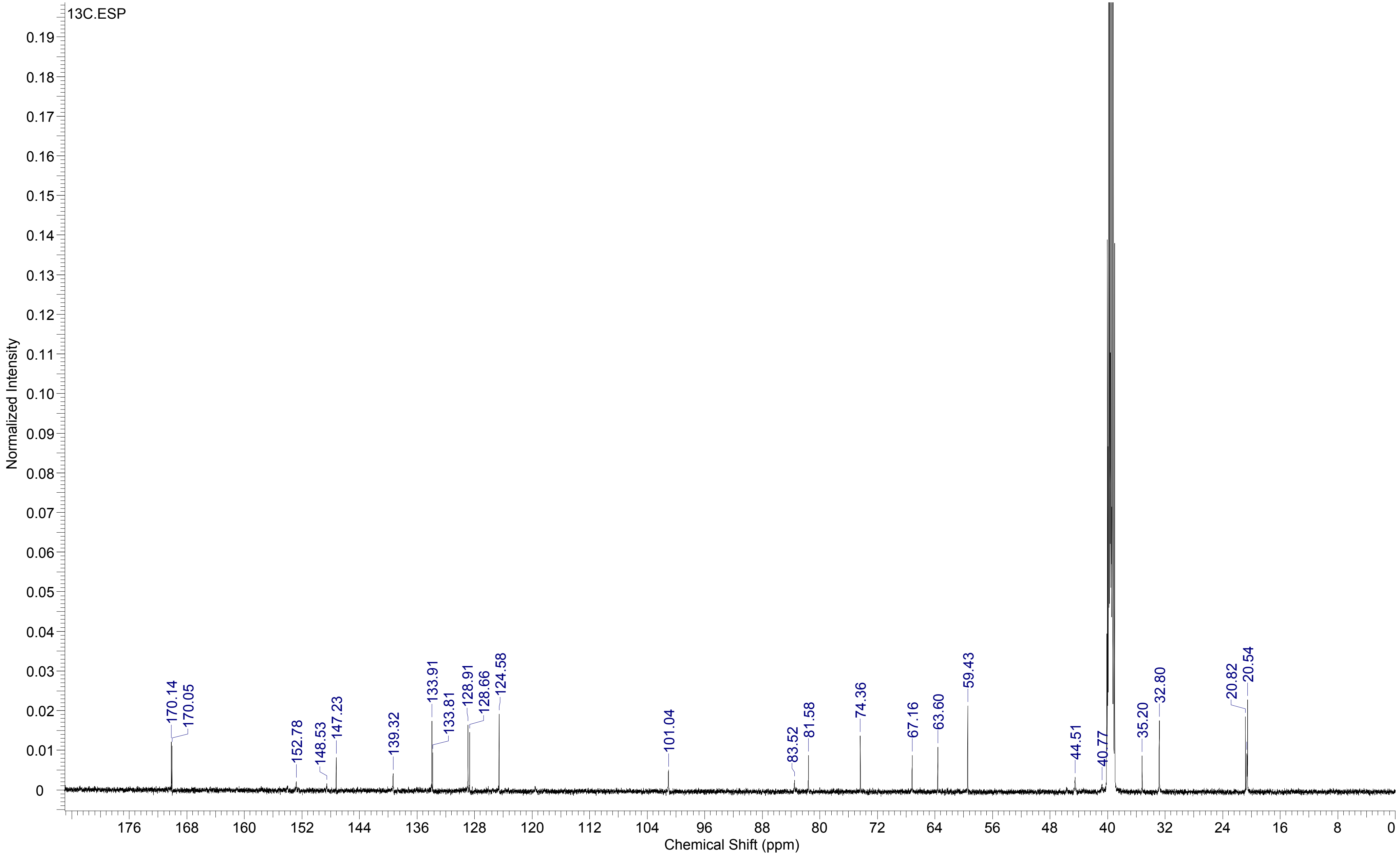

This report was created by ACD/NMR Processor Academic Edition. For more information go to www.acdlabs.com/nmrproc/

9-(2'-Deoxyribofuranosyl)-6-(2,2,6,6-tetramethyl-1-((2-nitrobenzyloxy)-methoxy)piperidin-4-ylamino) purine (15)

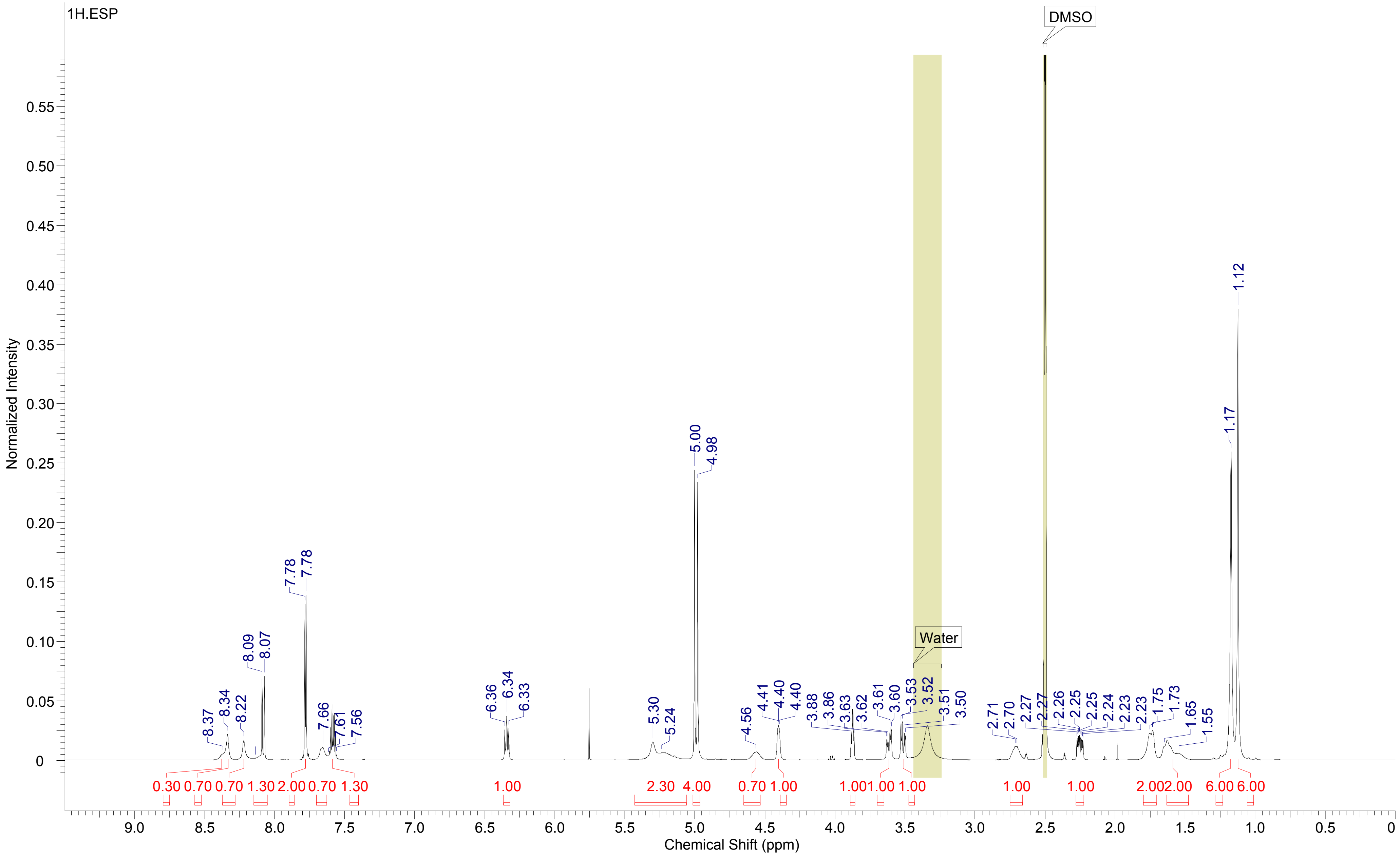

This report was created by ACD/NMR Processor Academic Edition. For more information go to www.acdlabs.com/nmrproc/

9-(2'-Deoxyribofuranosyl)-6-(2,2,6,6-tetramethyl-1-((2-nitrobenzyloxy)-methoxy)piperidin-4-ylamino) purine (15)

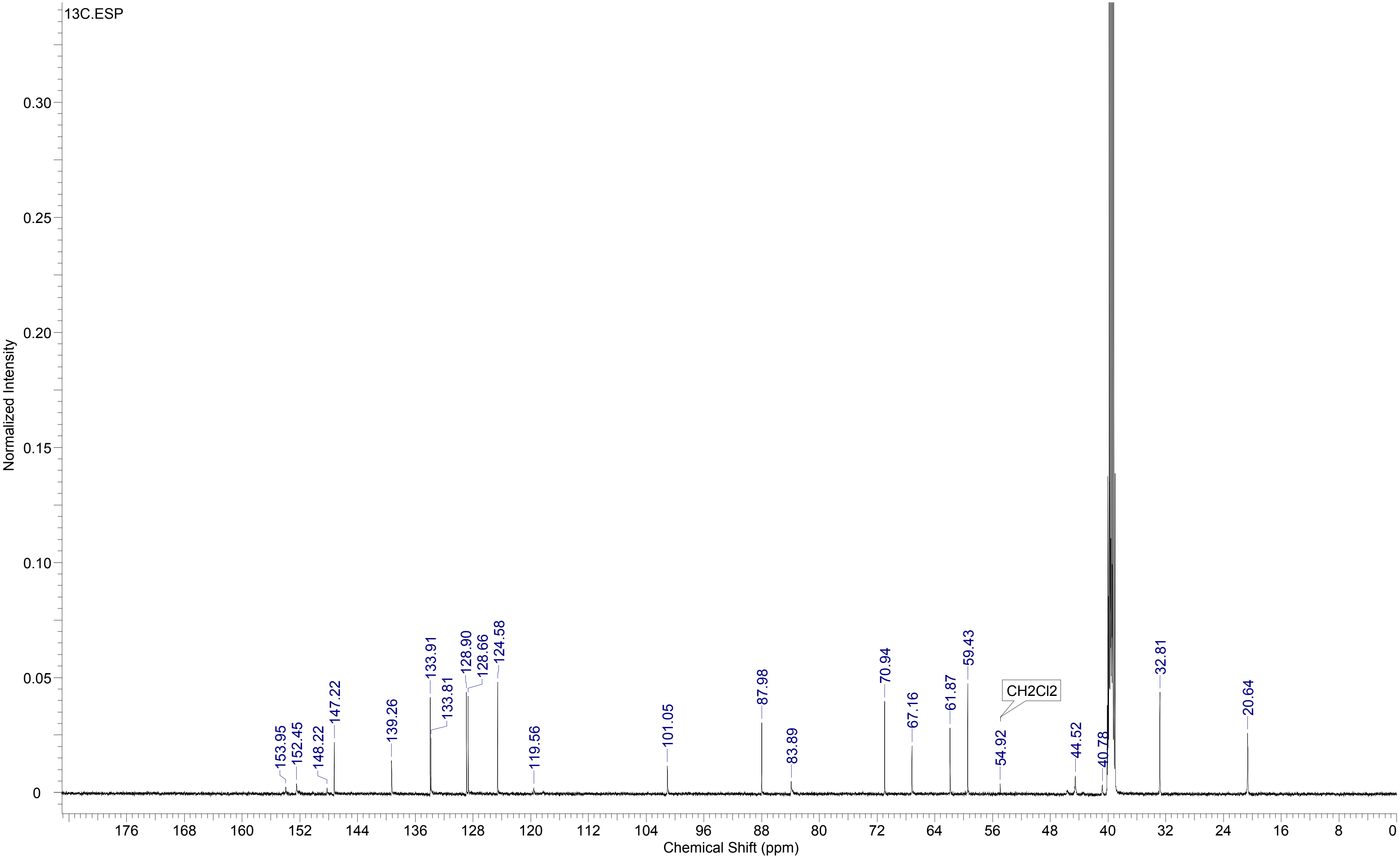

This report was created by ACD/NMR Processor Academic Edition. For more information go to www.acdlabs.com/nmrproc/

9-(5'-O-DMT-2'-Deoxyribofuranosyl)-6-(2,2,6,6-tetramethyl-1-((2-nitrobenzyloxy)-methoxy)piperidin-4-ylamino)purine (16)

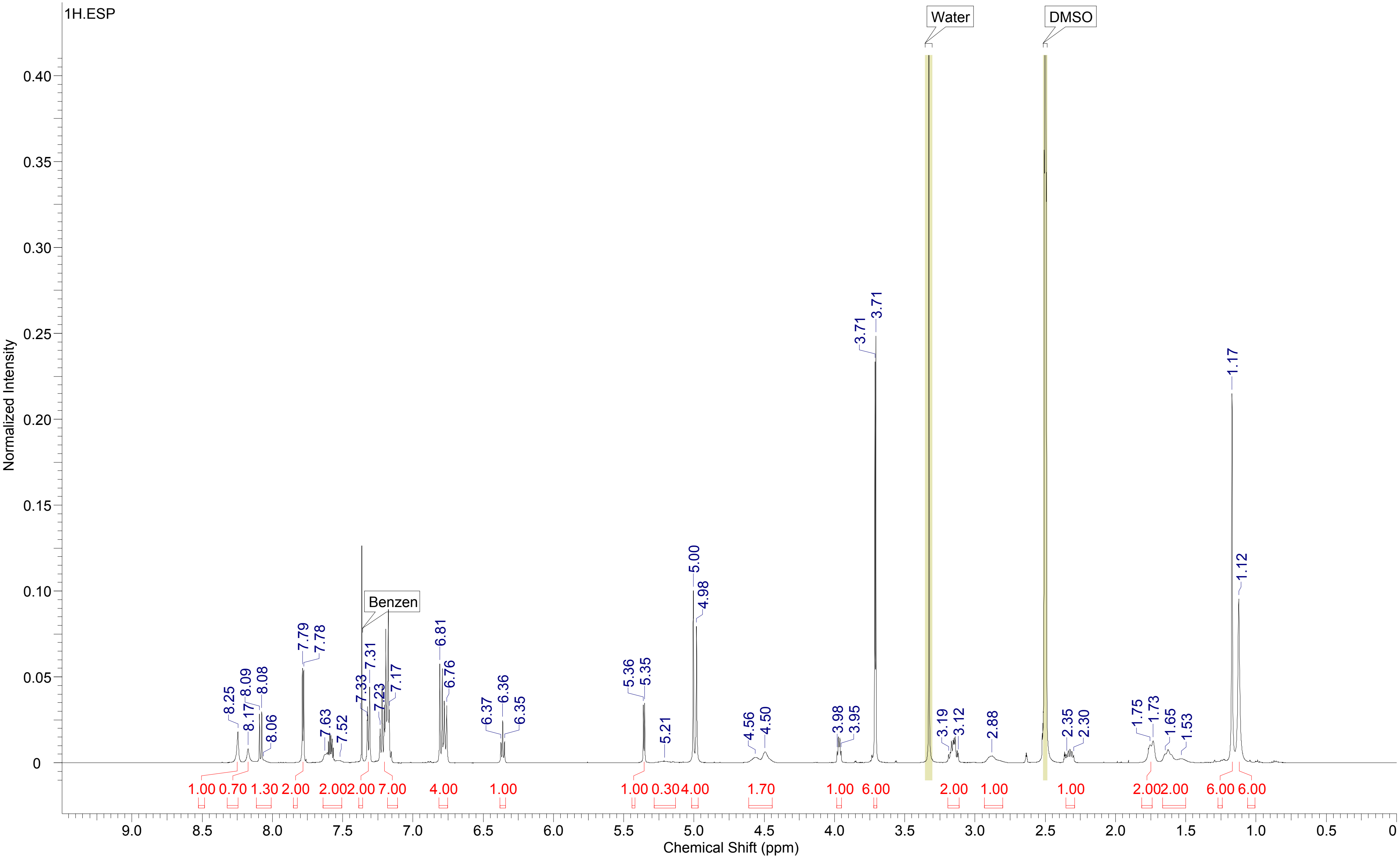

This report was created by ACD/NMR Processor Academic Edition. For more information go to www.acdlabs.com/nmrproc/

9-(5'-O-DMT-2'-Deoxyribofuranosyl)-6-(2,2,6,6-tetramethyl-1-((2-nitrobenzyloxy)-methoxy)piperidin-4-ylamino)purine (16)

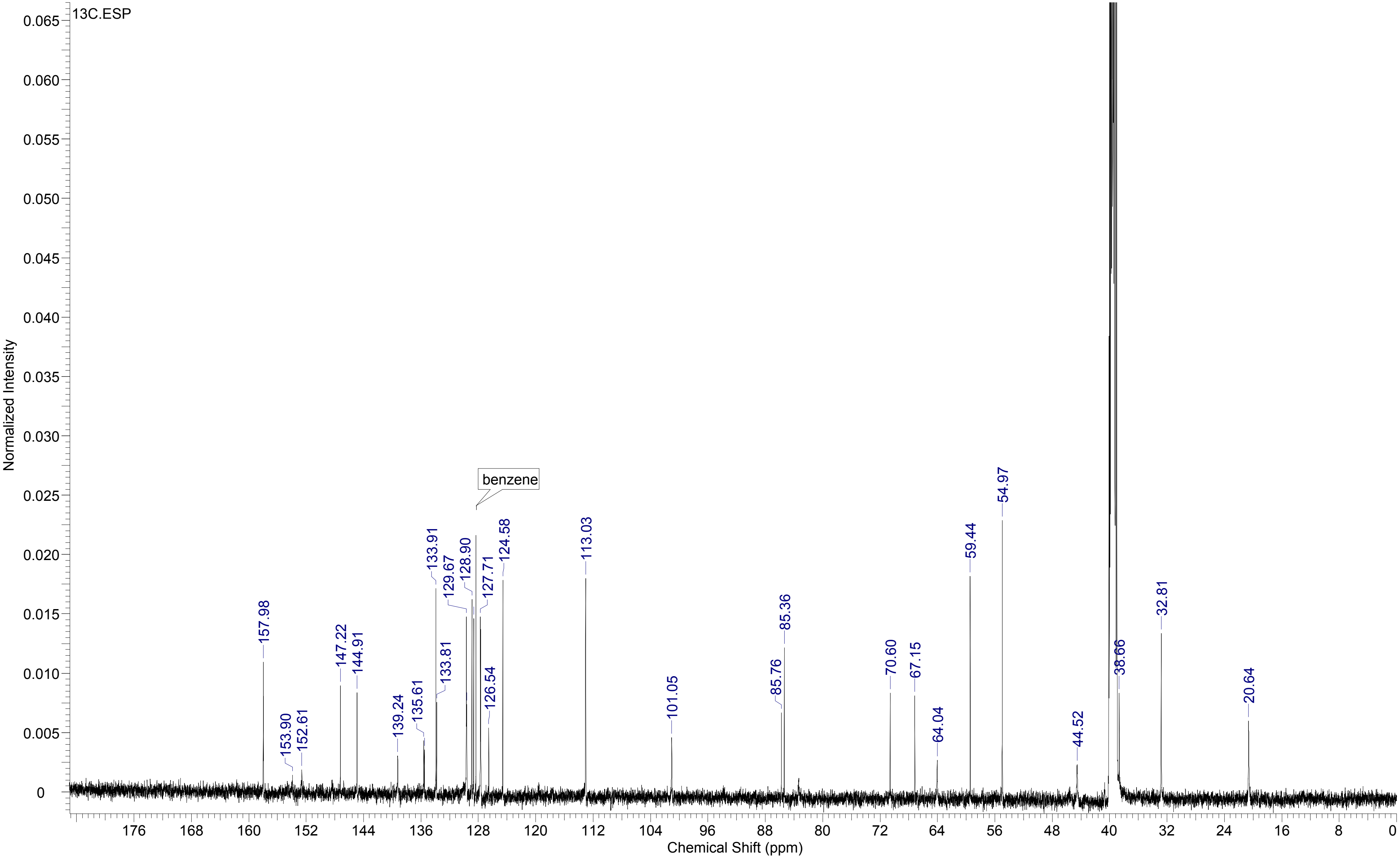

This report was created by ACD/NMR Processor Academic Edition. For more information go to www.acdlabs.com/nmrproc/

Deoxyadenosine phosphoramidite with protected spin label (7)

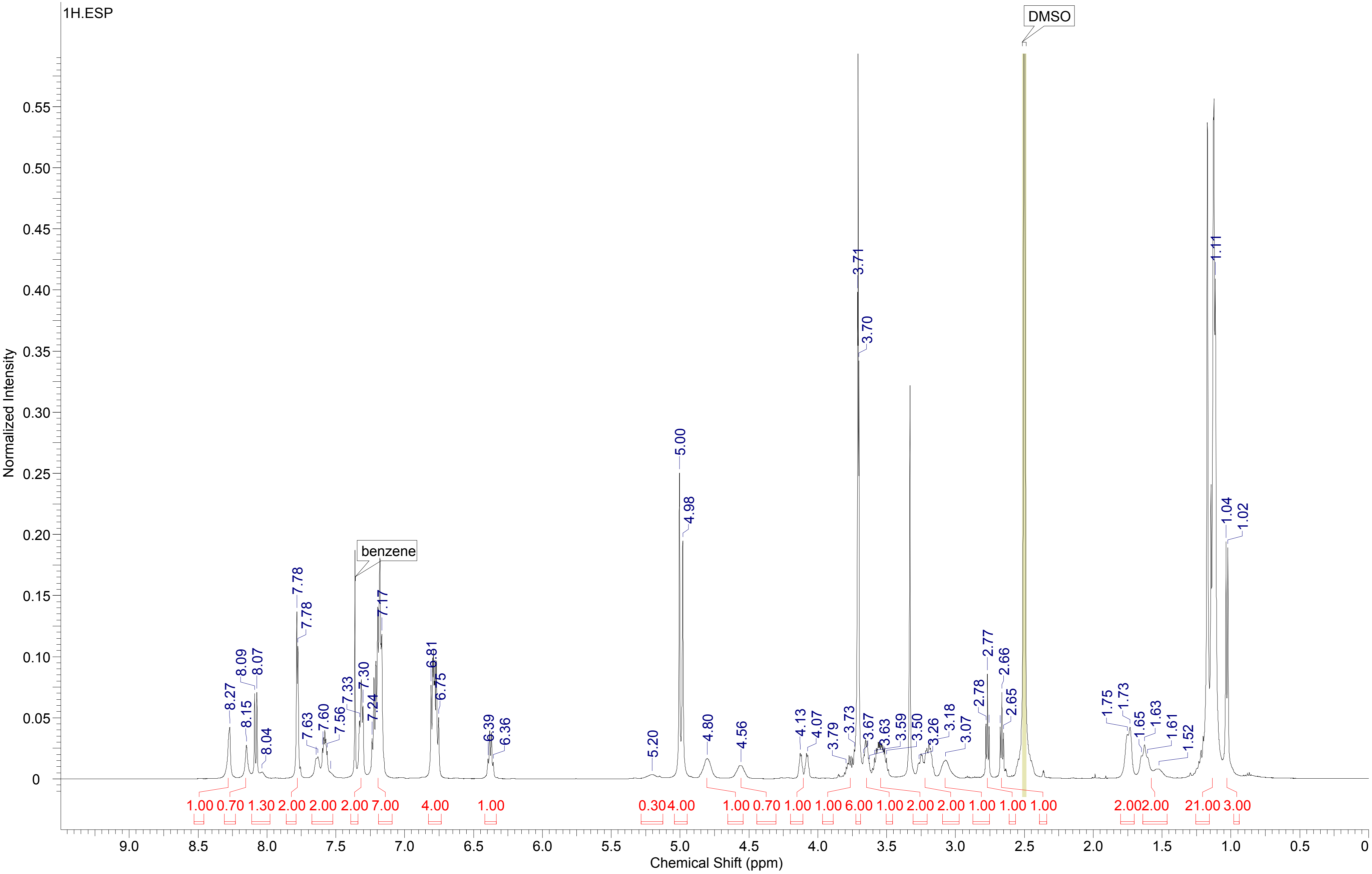

This report was created by ACD/NMR Processor Academic Edition. For more information go to www.acdlabs.com/nmrproc/

Deoxyadenosine phosphoramidite with protected spin label (7)

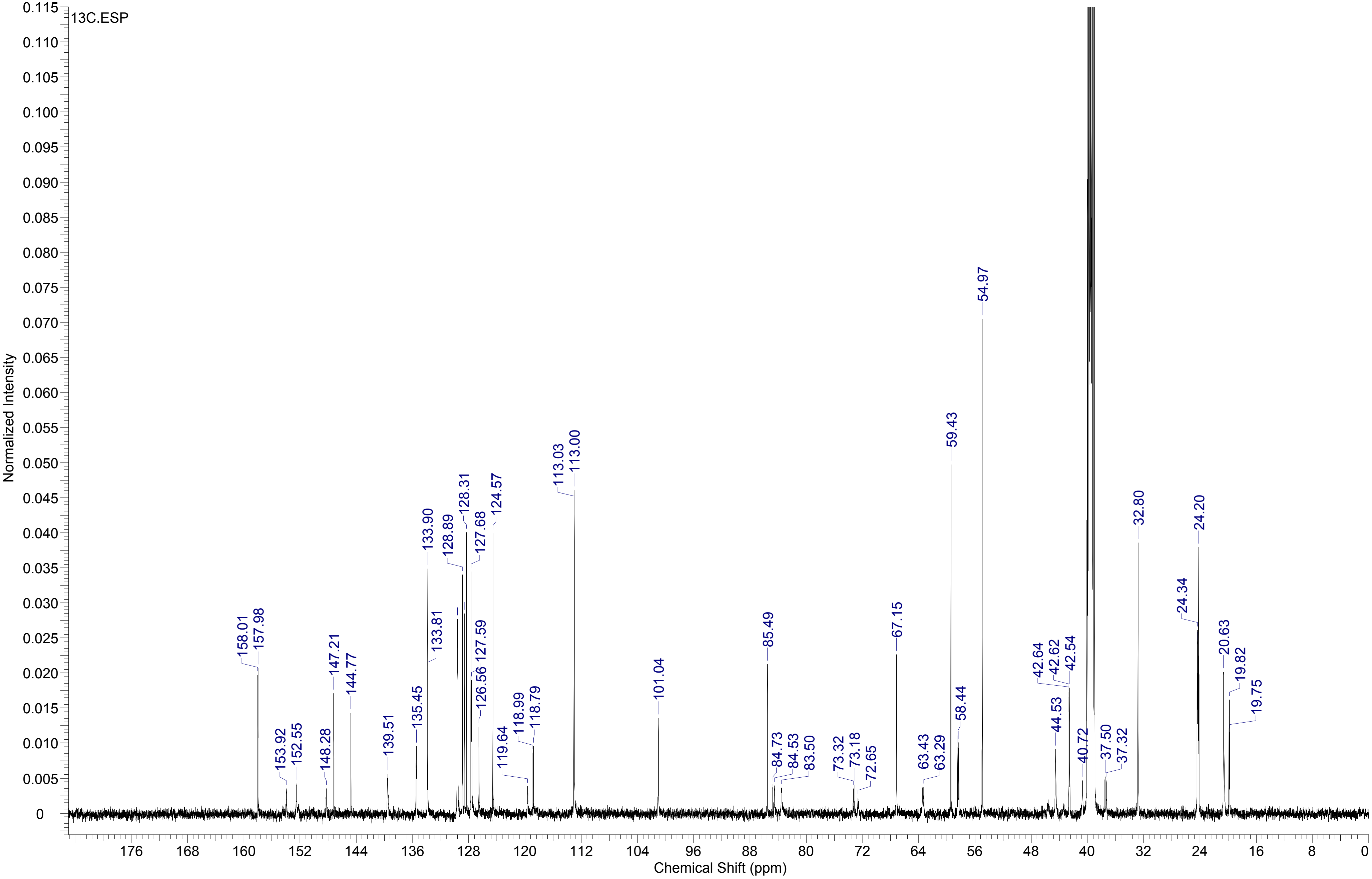

This report was created by ACD/NMR Processor Academic Edition. For more information go to www.acdlabs.com/nmrproc/

Deoxyadenosine phosphoramidite with protected spin label (7)

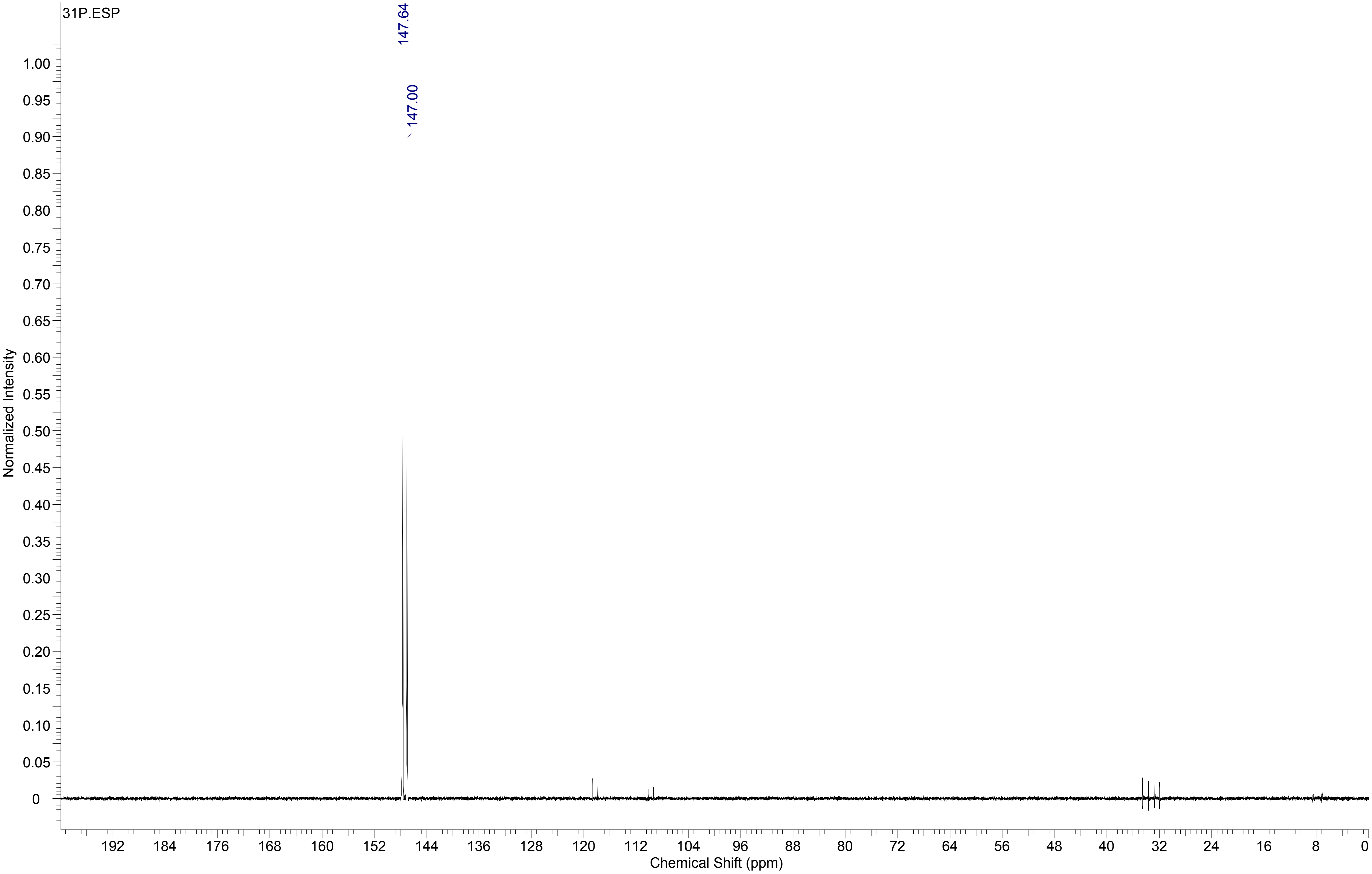

This report was created by ACD/NMR Processor Academic Edition. For more information go to www.acdlabs.com/nmrproc/

9-(2',3',5'-Tri-O-acetyl-ribofuranosyl)-6-chloro-purine (17)

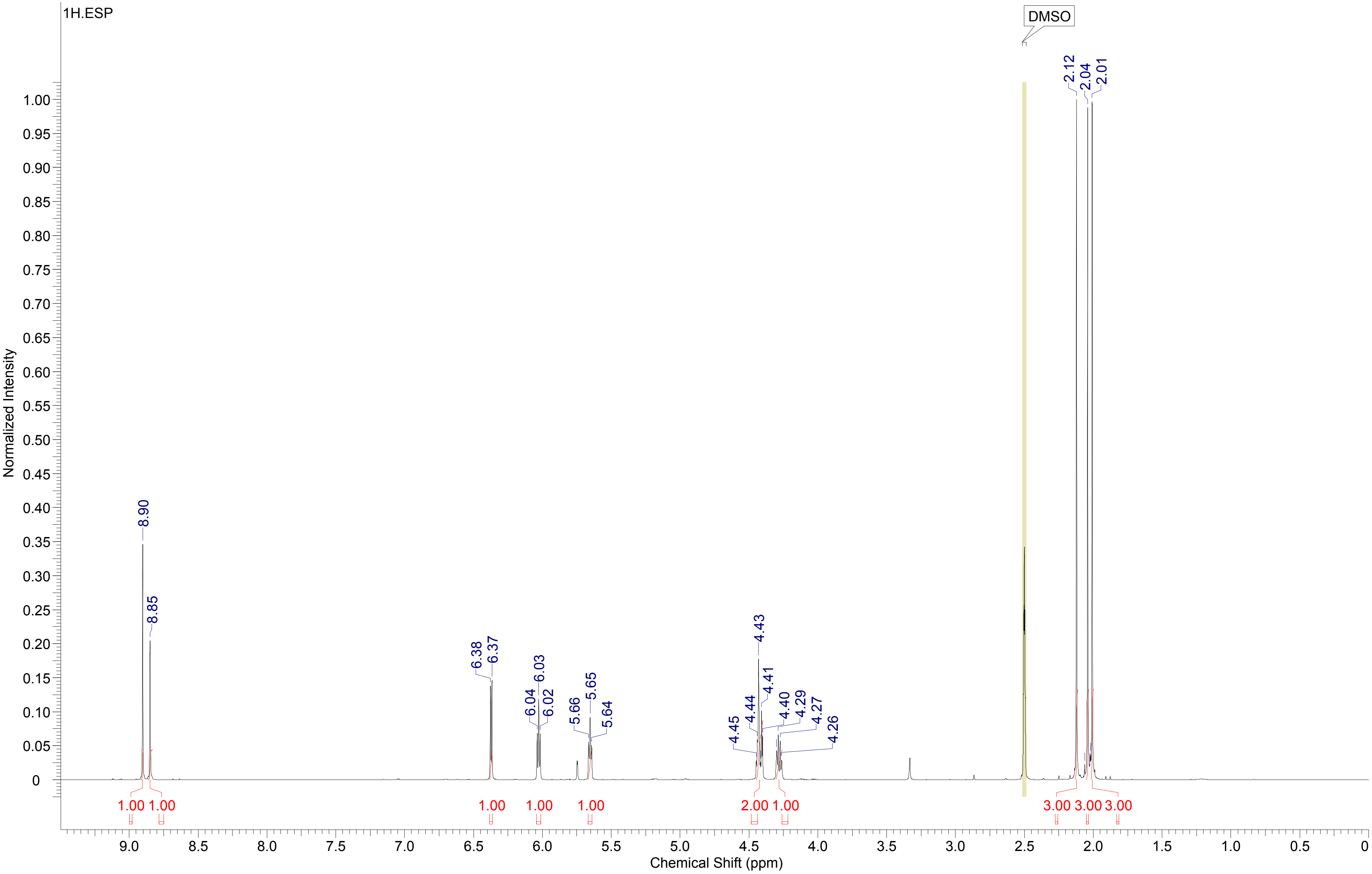

This report was created by ACD/NMR Processor Academic Edition. For more information go to www.acdlabs.com/nmrproc/

9-(2',3',5'-Tri-O-acetyl-ribofuranosyl)-6-chloro-purine (17)

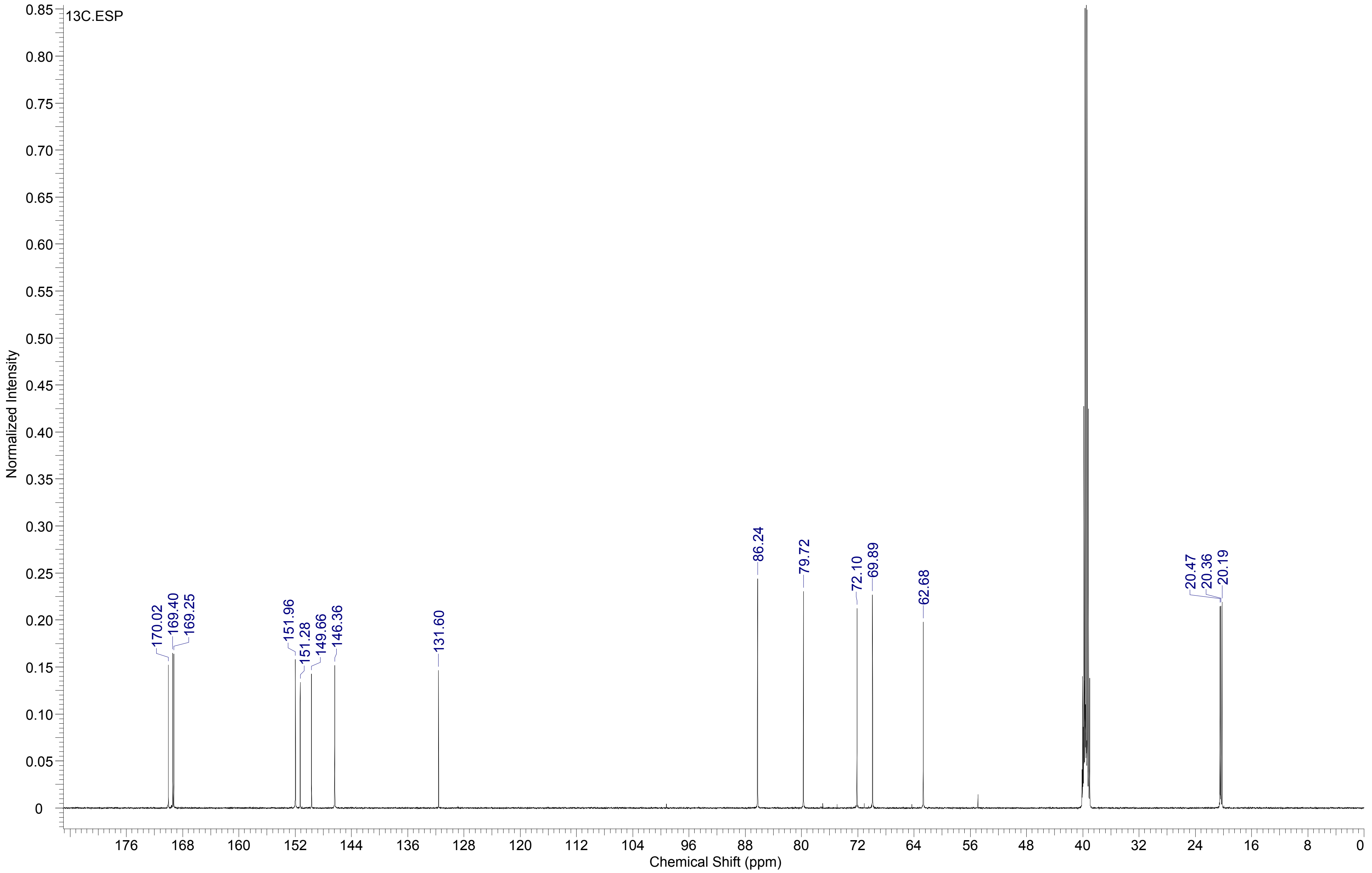

This report was created by ACD/NMR Processor Academic Edition. For more information go to www.acdlabs.com/nmrproc/

9-(2',3',5'-Tri-O-acetyl-ribofuranosyl)-6-(2,2,6,6-tetramethyl-1-((2-nitrobenzyloxy)methoxy)-piperidin-4-ylamino)purine (18)

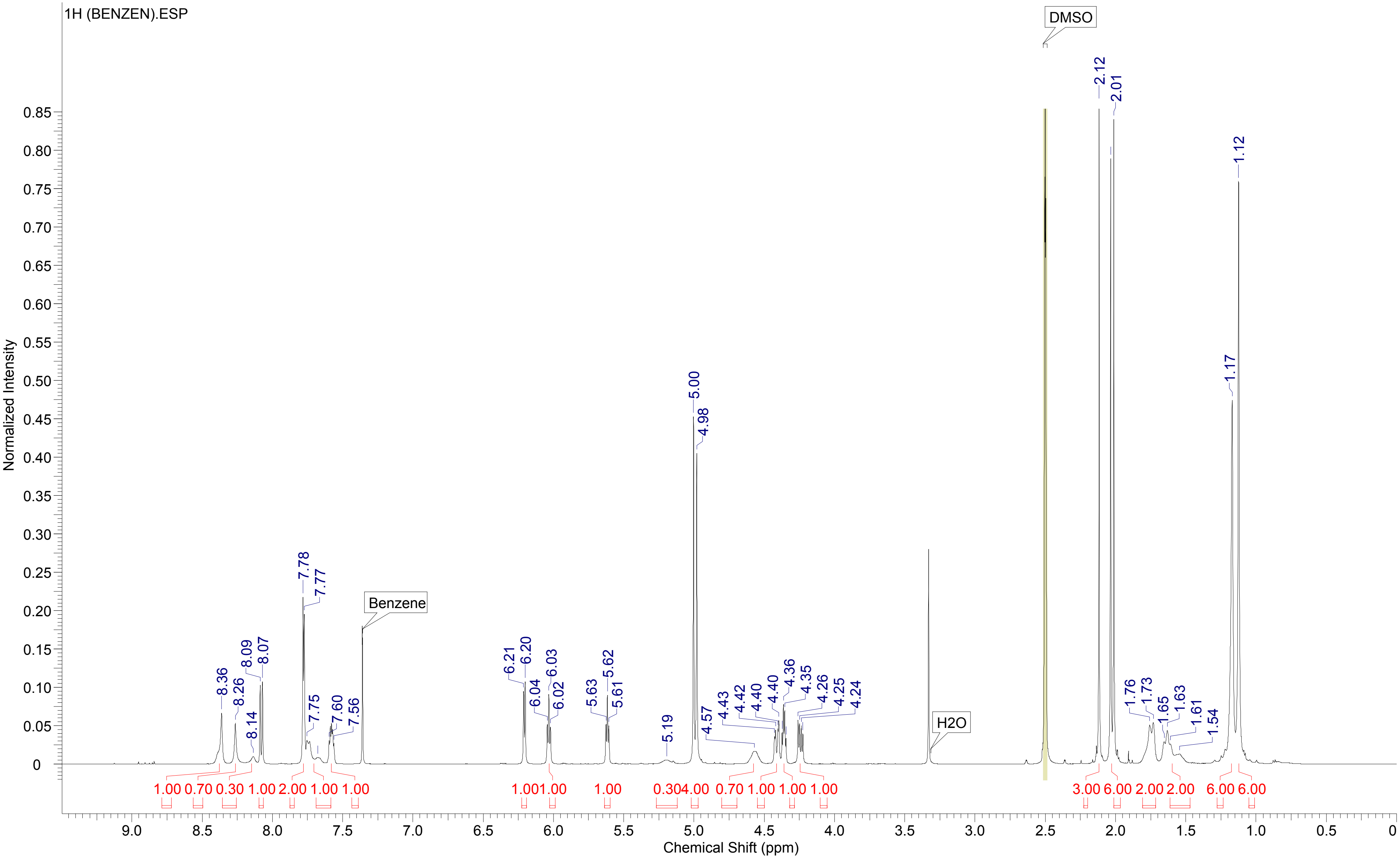

This report was created by ACD/NMR Processor Academic Edition. For more information go to www.acdlabs.com/nmrproc/

9-(2',3',5'-Tri-O-acetyl-ribofuranosyl)-6-(2,2,6,6-tetramethyl-1-((2-nitrobenzyloxy)methoxy)-piperidin-4-ylamino)purine (18)

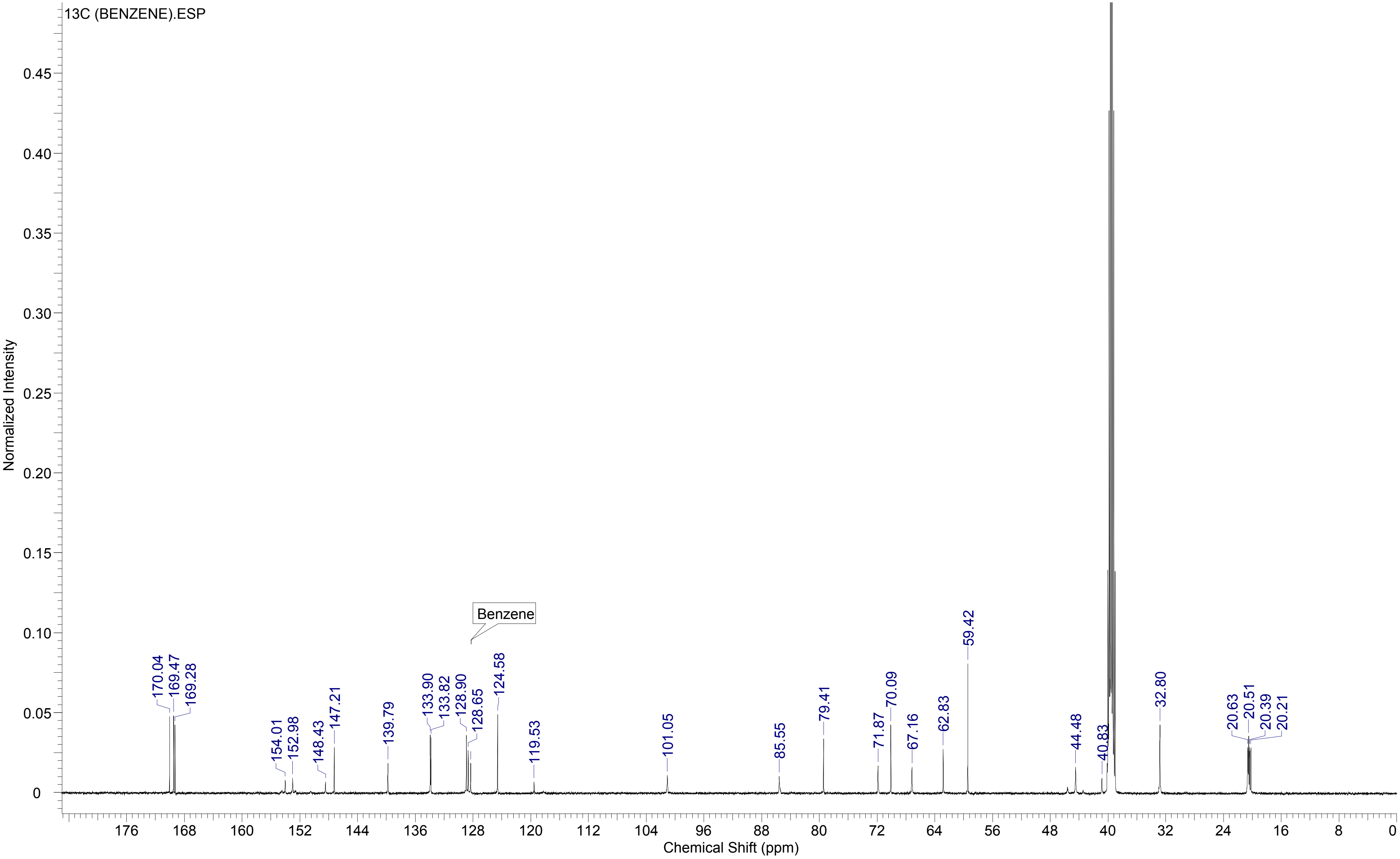

This report was created by ACD/NMR Processor Academic Edition. For more information go to www.acdlabs.com/nmrproc/

9-(Ribofuranosyl)-6-(2,2,6,6-tetramethyl-1-((2-nitrobenzyloxy)-methoxy)piperidin-4-ylamino)-purine (19)

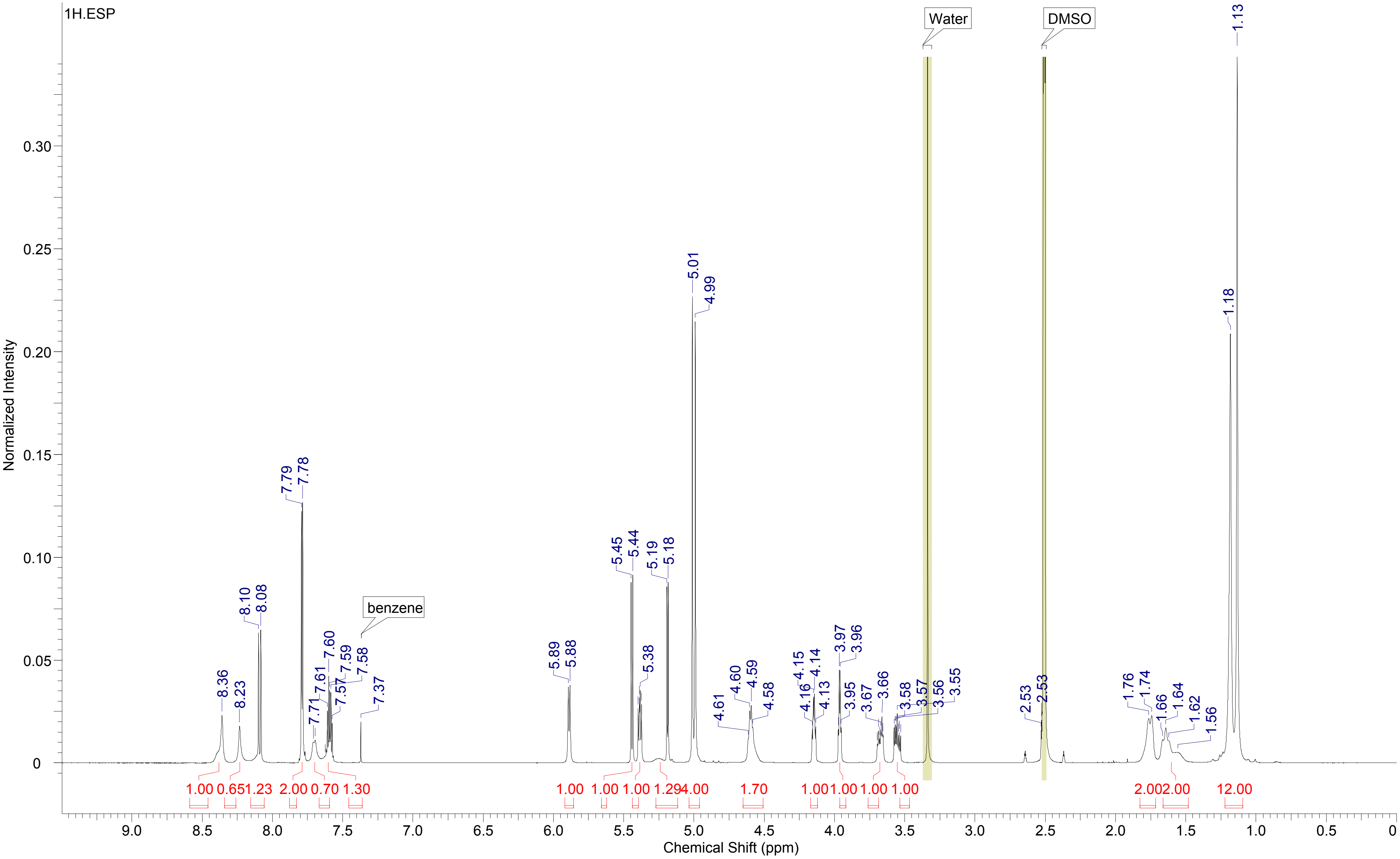

This report was created by ACD/NMR Processor Academic Edition. For more information go to www.acdlabs.com/nmrproc/

9-(Ribofuranosyl)-6-(2,2,6,6-tetramethyl-1-((2-nitrobenzyloxy)-methoxy)piperidin-4-ylamino)-purine (19)

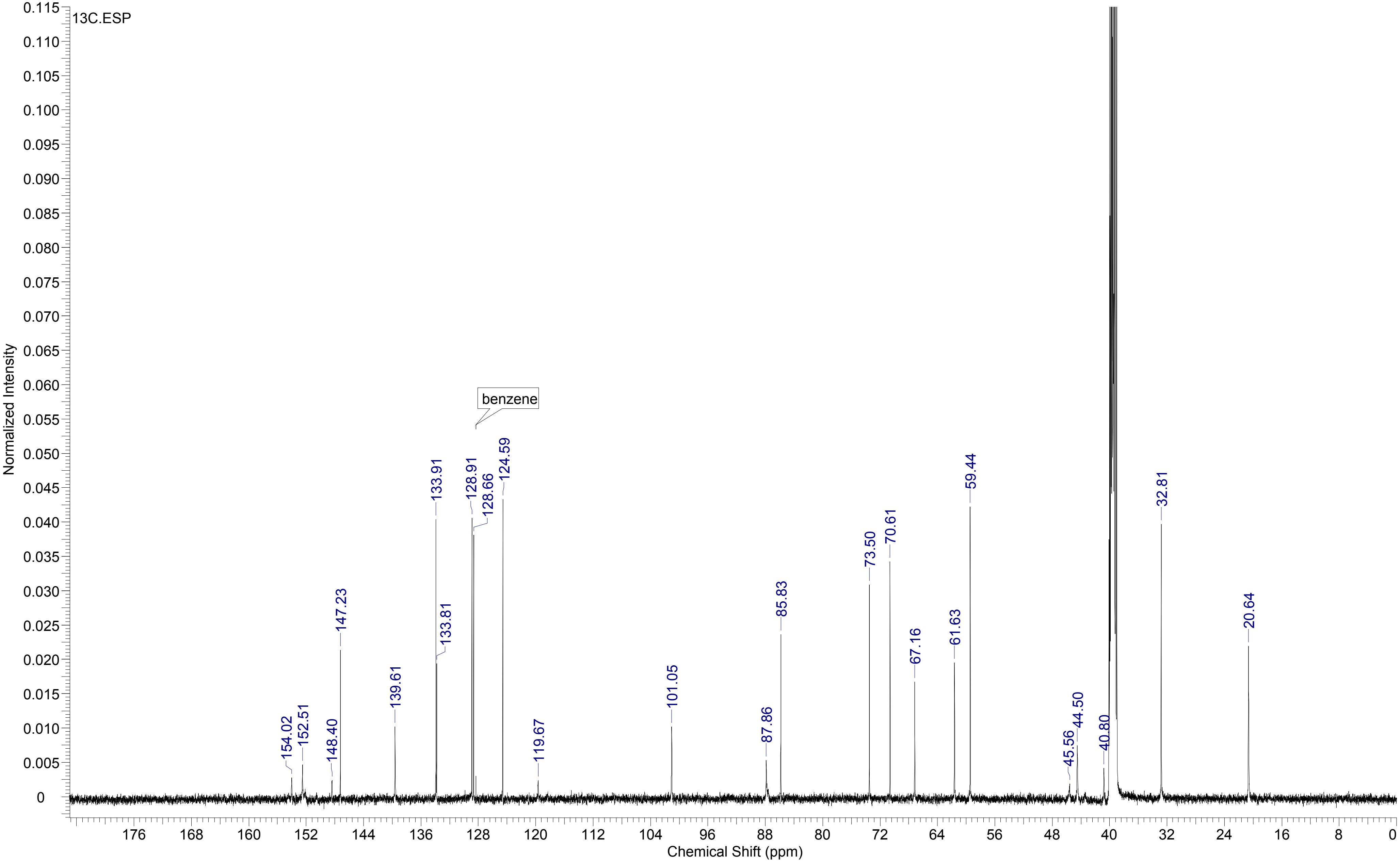

This report was created by ACD/NMR Processor Academic Edition. For more information go to www.acdlabs.com/nmrproc/

9-(5'-O-DMT-Ribofuranosyl)-6-(2,2,6,6-tetramethyl-1-((2-nitrobenzyloxy)-methoxy)piperidin-4-ylamino) purine (20)

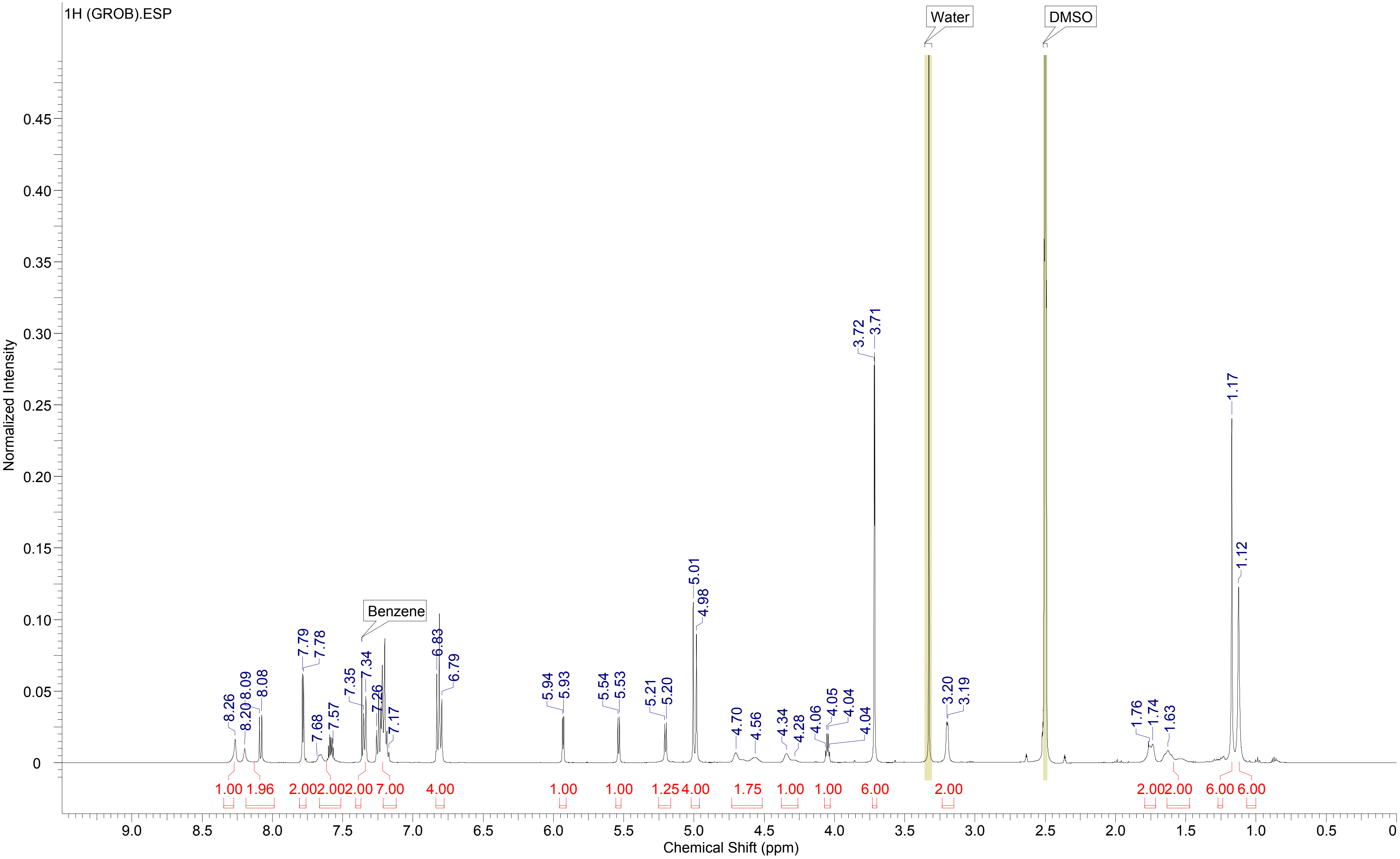

This report was created by ACD/NMR Processor Academic Edition. For more information go to www.acdlabs.com/nmrproc/

9-(5'-O-DMT-Ribofuranosyl)-6-(2,2,6,6-tetramethyl-1-((2-nitrobenzyloxy)-methoxy)piperidin-4-ylamino) purine (20)

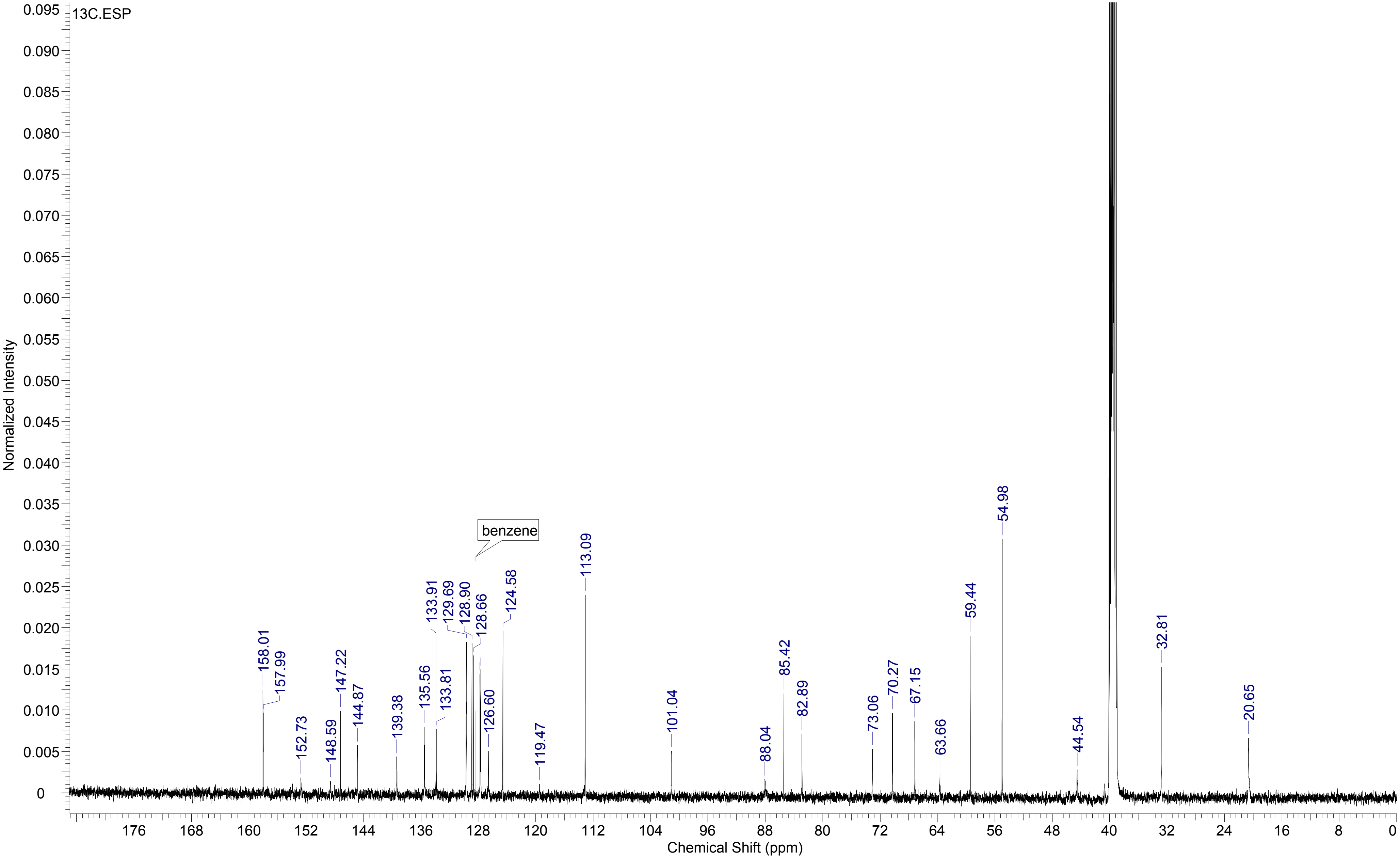

This report was created by ACD/NMR Processor Academic Edition. For more information go to www.acdlabs.com/nmrproc/

9-(2'-O-TBS-5'-O-DMT-Ribofuranosyl)-6-(2,2,6,6-tetramethyl-1-((2-nitrobenzyloxy)-methoxy)piperidin-4 ylamino)purine (21)

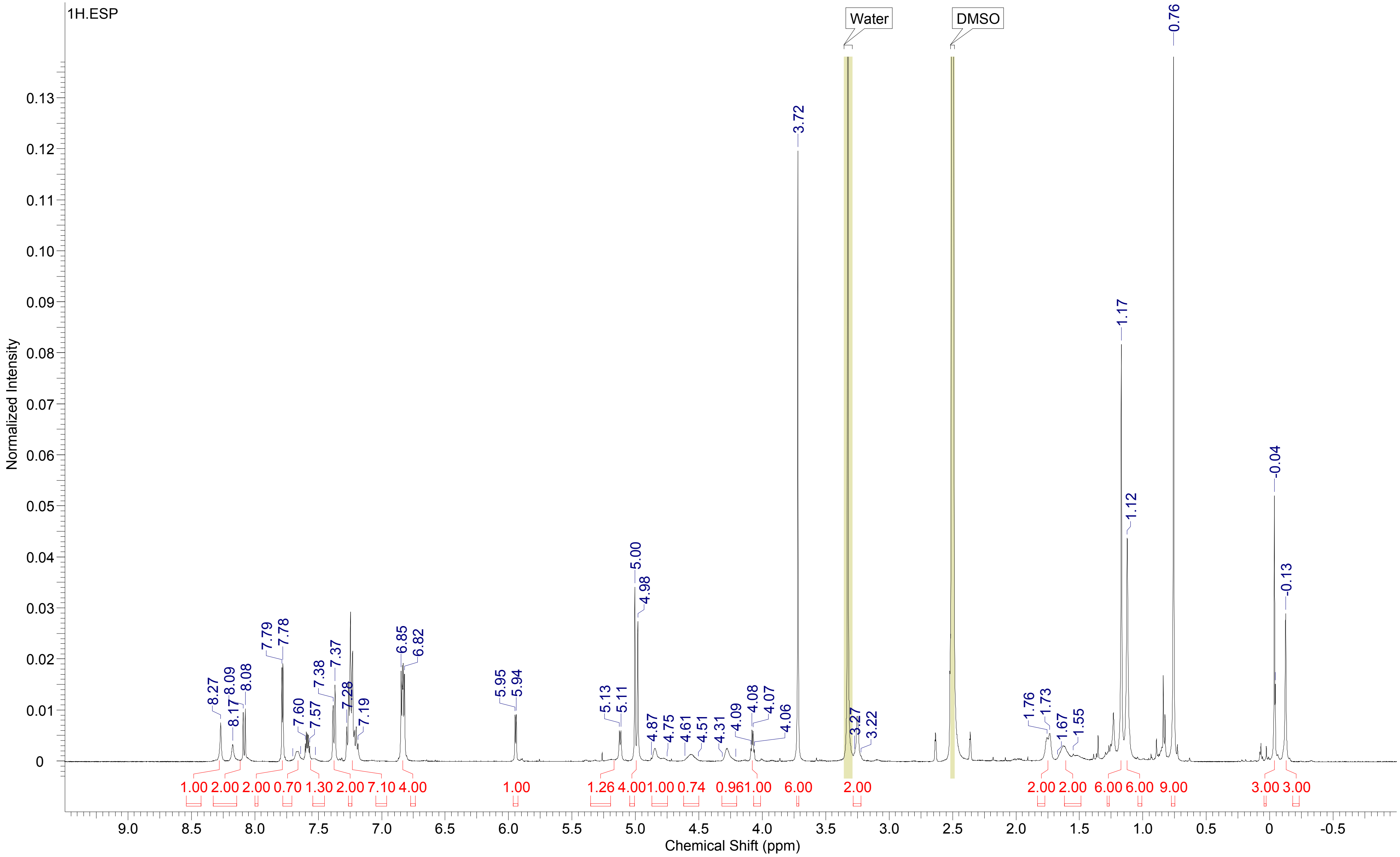

This report was created by ACD/NMR Processor Academic Edition. For more information go to www.acdlabs.com/nmrproc/

9-(2'-O-TBS-5'-O-DMT-Ribofuranosyl)-6-(2,2,6,6-tetramethyl-1-((2-nitrobenzyloxy)-methoxy)piperidin-4 ylamino)purine (21)

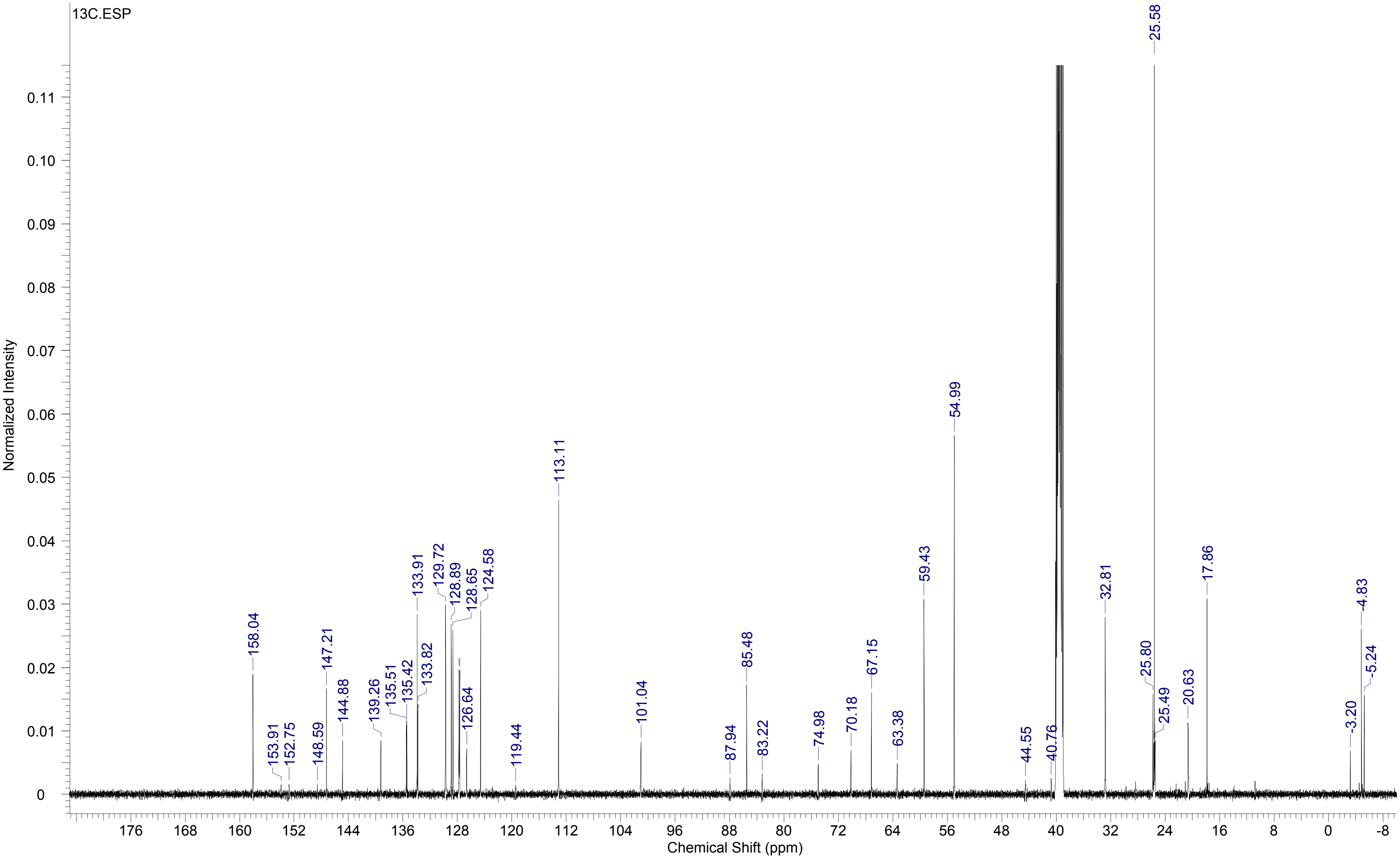

This report was created by ACD/NMR Processor Academic Edition. For more information go to www.acdlabs.com/nmrproc/

Adenosine phosphoramidite with protected spin label (8)

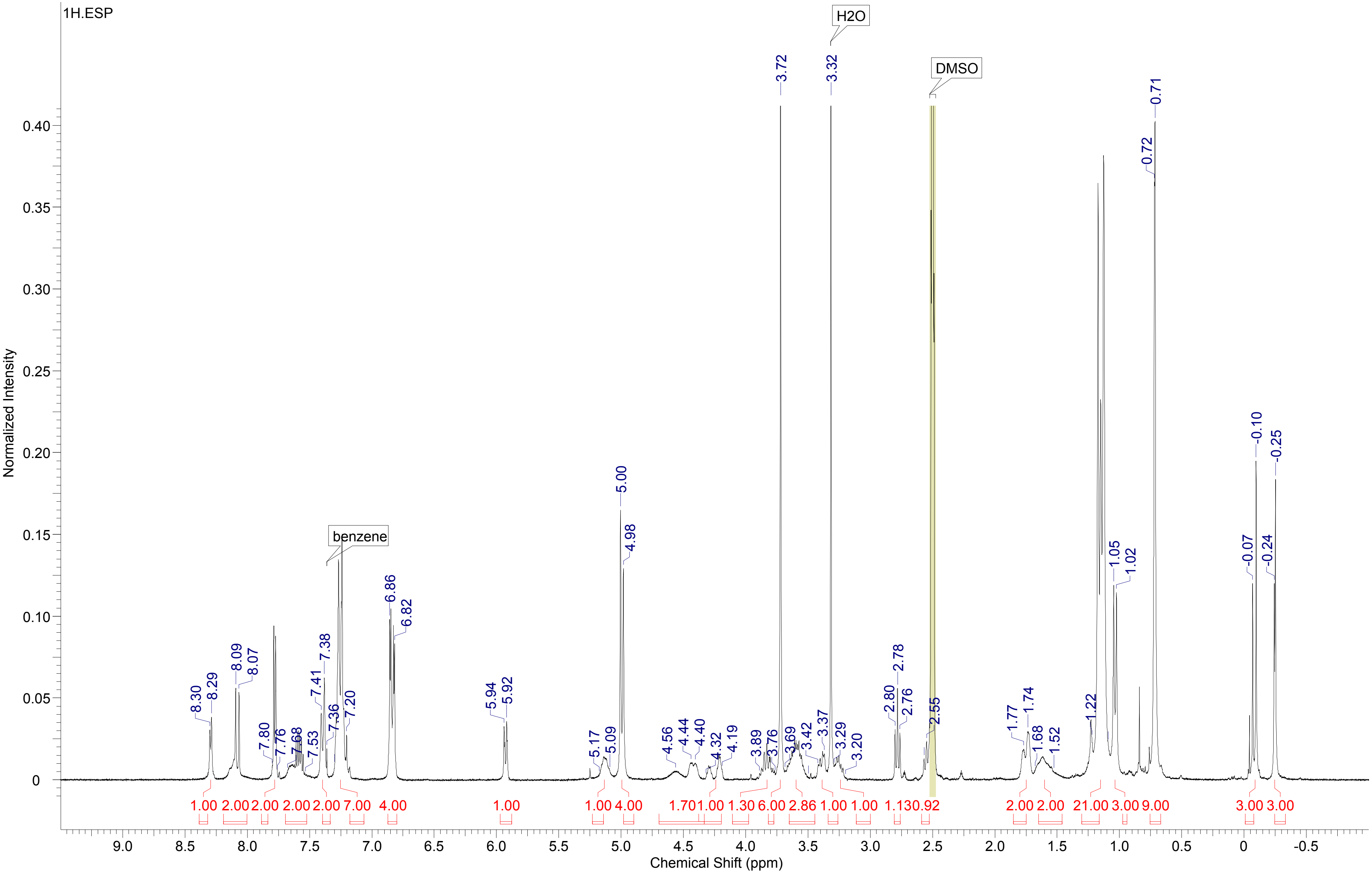

This report was created by ACD/NMR Processor Academic Edition. For more information go to www.acdlabs.com/nmrproc/

Adenosine phosphoramidite with protected spin label (8)

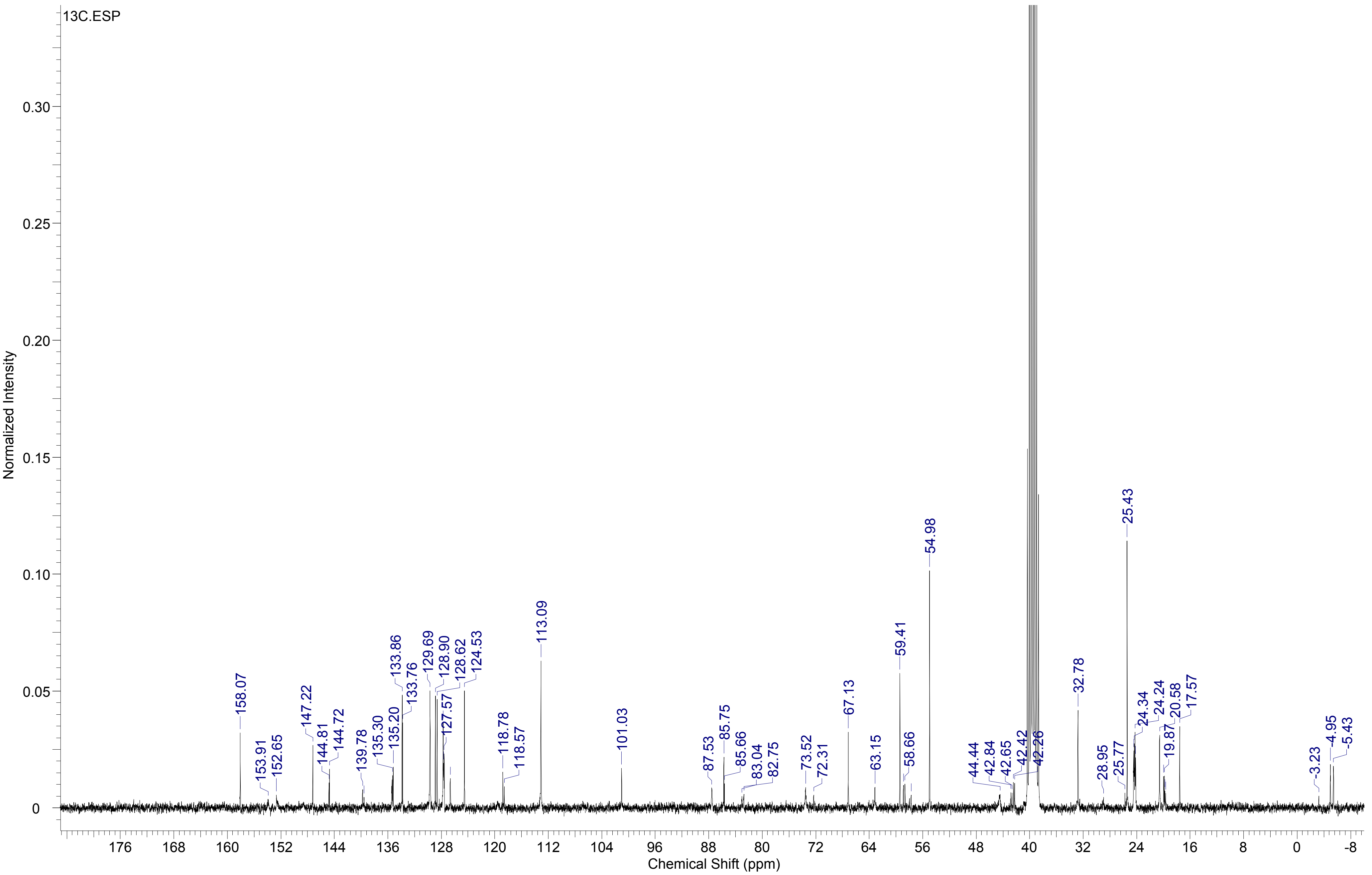

This report was created by ACD/NMR Processor Academic Edition. For more information go to www.acdlabs.com/nmrproc/

Adenosine phosphoramidite with protected spin label (8)

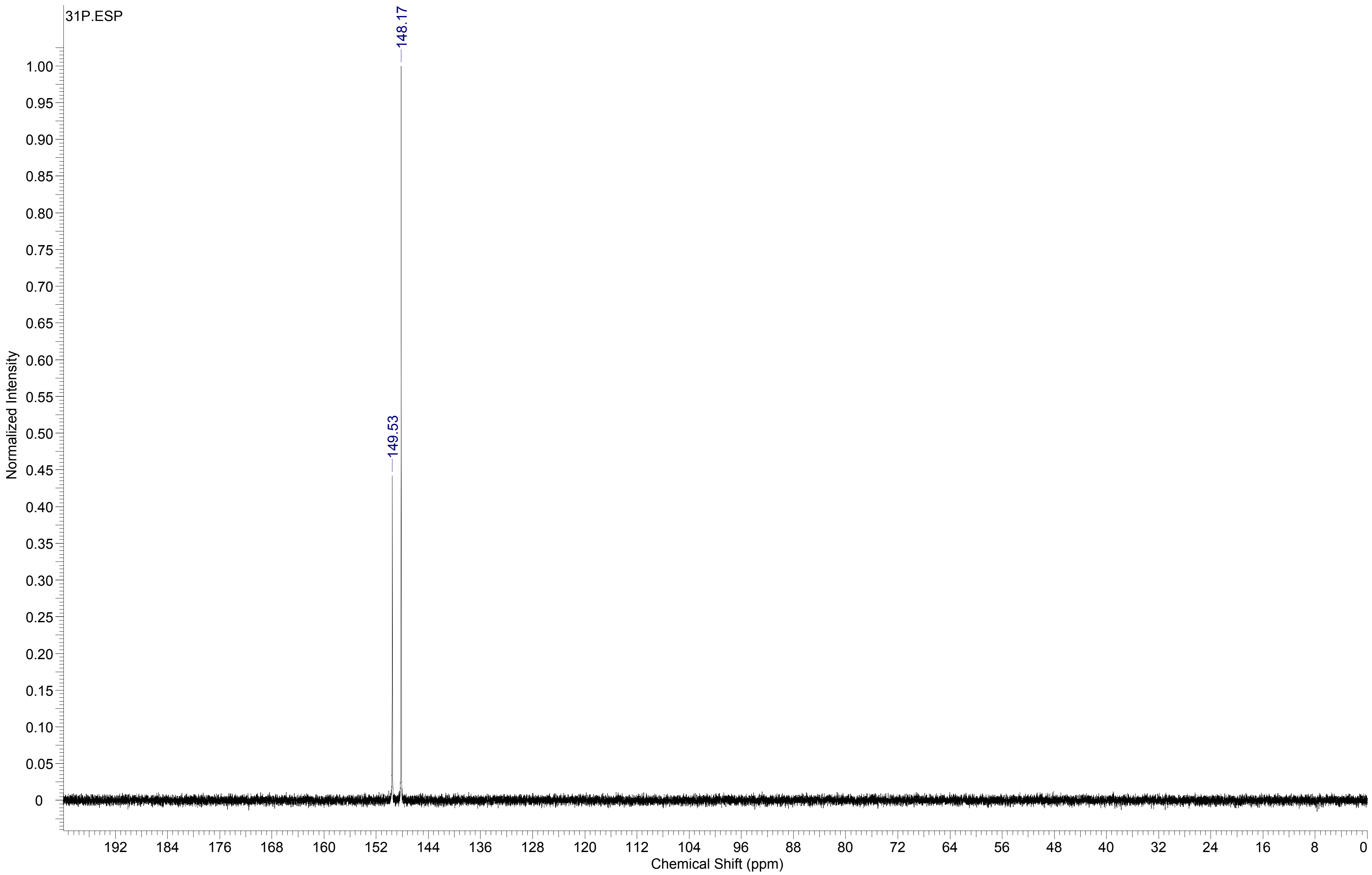

Supplement: File 1 — Synthesis, purification and photochemical deprotection of oligonucleotides, mass spectra and HPLC plots. 1H and 13C NMR spectra. [file Beilstein_J_Org_Chem-14-1563-s001.pdf]
